# Relativistic Quantum Chemical Investigation of Actinide Covalency Measured by Electron Paramagnetic Resonance Spectroscopy

**DOI:** 10.1021/jacs.4c01930

**Published:** 2024-05-16

**Authors:** Letitia Birnoschi, Meagan S. Oakley, Eric J. L. McInnes, Nicholas F. Chilton

**Affiliations:** † Department of Chemistry, The University of Manchester Oxford Road, Manchester M13 9PL, U.K.; ‡ Research School of Chemistry, The Australian National University, Sullivans Creek Road, Canberra, Acton 2601, Australia

## Abstract

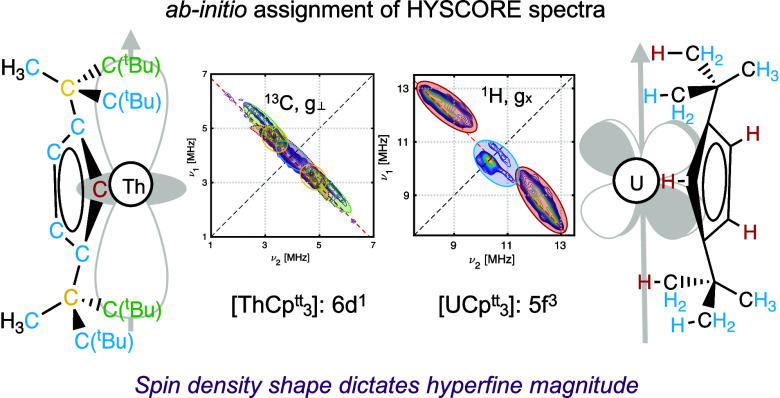

We investigate actinide covalency effects in two [AnCp^tt^_3_] (An = Th, U) complexes recently studied with pulsed electron paramagnetic resonance spectroscopy, using the Hyperion package to obtain relativistic hyperfine coupling constants from relativistic multiconfigurational wave functions. ^1^H and ^13^C HYSCORE simulations using the computed parameters show excellent agreement with the experimental data, highlighting the accuracy of modern relativistic ab initio methods. The extent of covalency indicated from the calculations on [ThCp^tt^_3_] is in agreement with the original report based on traditional spectral fitting methods, while the covalency in [UCp^tt^_3_] is found to be previously overestimated. The latter is due to the paramagnetic spin–orbit effect that arises naturally in a relativistic theory of hyperfine coupling and yet was not accounted for in the original study, thus highlighting the necessity of relativistic approaches for the interpretation of magnetic resonance data pertaining to actinides.

## 1 Introduction

The actinide (An) elements present a key area of interest within chemical science, attracting research efforts from experimentalists and theorists alike.^1−4^ Despite this interest, the inherent radiological hazards of working with An elements and the need for specialist equipment have made progress in this area slow. To this day, our understanding of An bonding and properties lags behind those of other regions of the periodic table. Of particular interest is the enhanced covalency in An-ligand bonding compared to lanthanides, believed to arise due to the more expanded nature of 5f orbitals relative to 4f orbitals^5^ in the predominantly ionic lanthanide (Ln) elements. Such effects are believed to be important in An/Ln separation for more robust treatment of nuclear wastewater.^6^

An chemistry is influenced by strong relativistic effects, as well as nontrivial electronic structures, where 5f, 6d, and 7s valence orbitals are all involved in bonding. This combination is rarely observed in the rest of the periodic table; hence, insights into An properties based on periodic trends is severely limited. As such, hybrid approaches involving both experimental and theoretical methods are indispensable for modern An chemistry. Indeed, X-ray absorption^7,8^ and magnetic resonance techniques^9−11^ are now popular choices for studies of An covalency, often complemented by computational electronic structure calculations.

In 2017, Formanuik et al. published the first pulsed electron paramagnetic resonance (EPR) study of molecular An complexes,^9^ with a focus on correlating ligand hyperfine coupling (HFC) measurements with spin density at the ligand nuclei and hence covalency in [AnCp^tt^_3_] [An = Th(III), U(III); Cp^tt^ = 1,3–^*t*^Bu_2_–C_5_H_3_] complexes. The strength of HFC, quantified by hyperfine coupling constants (HFCCs), depends on the unpaired electron (spin) density distribution and varies as *r*_N_^–3^, where *r*_N_ is the distance between magnetic nucleus N and the spin density. Under traditional nonrelativistic interpretations, isotropic HFCCs arise solely due to spin density at the nucleus,^12^ which can only arise from the unpaired electron spins residing in orbitals with *s*-character, as these are the only atomic orbitals which do not have nodes at the nucleus; hence, isotropic HFCCs report directly on spin delocalization. The picture is not so clear in relativistic theory,^13^ but still, small variations in the spin density distribution to metal and/or ligand nuclei lead to noticeable changes in HFCCs, and hence, measurement of HFC is a particularly good probe for covalency.^14^ Formanuik et al. employed hyperfine sublevel correlation spectroscopy (HYSCORE) to study HFC of ^1^H and ^13^C ligand nuclei in [AnCp^tt^_3_] complexes. They found that the experimental hyperfine interactions could not be explained by a classical point-dipole model based on an effective electron spin-half approximation using the experimentally determined *g*-values and the assumption that electron spins were confined to the metal ions. This allowed them to estimate atomic spin densities for the ligand atoms to best represent the experimental HYSCORE spectra.

However, it is not known how reliable it is to interpret An HFCCs using a nonrelativistic model, even if spin–orbit coupling (SOC) effects are accounted for implicitly by using experimentally observed effective *g*-values of the ground Kramers doublet. Instead, the most appropriate approach would be to use a relativistic quantum chemical model based on the Dirac equation or a variant thereof.^15^ Aside from the ability to account for relativistic effects at the appropriate level of theory, quantum chemical methods can provide direct estimates for spin populations independent of HFCCs. Here, the calculation of HFCCs serves as a precise benchmark for electronic structure calculations.

Recently, we have developed the Hyperion code for computing relativistic HFCCs from ab initio multi configurational electronic structure calculations.^16^ We provide a brief sketch of the theory in the Supporting Information (see Section S1) for the interested reader, but the basic thrust of our approach is to use complete active space and restricted active space self-consistent field methods (CASSCF/RASSCF)^17,18^ to account for spin polarization/electron correlation in a flexible manner and, where necessary, additionally included SOC via a state-interaction approach (CASSCF-SO/RASSCF-SO),^19^ all built on top of a scalar-relativistic eXact-2-Component (SR-X2C) decoupled Hamiltonian.^20−33^ Our implementation defines spin-dependent and spin-independent contributions, which in the nonrelativistic picture correspond to the sum of the Fermi-coupling (FC) and spin-dipolar (SD) (former contribution), and the paramagnetic spin–orbit (PSO) term (latter contribution) which accounts for the interaction between the electronic orbital angular momentum and the nuclear spin. For systems where *S* is not a good quantum number, we use the pseudospin  parametrization, where the model space (pseudospin multiplet) encompasses the lowest-energy  states in the SO-coupled spectrum. We note that the  quantum number could represent a true spin *S* subject to zero-field splitting, a total spin–orbit coupled angular momentum *J* subject to a crystal field splitting, or the ground Kramers doublet of a crystal-field-split *J* multiplet, for example. We have previously shown that our method yields very good results for a wide selection of atomic systems, and note that it is similar to methods developed by Autschbach and co-workers.^34,35^

**Figure 1:**
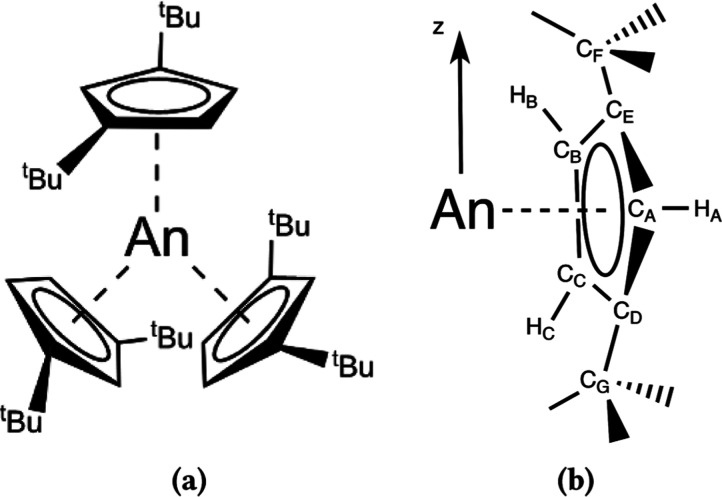
(a) Schematic of the [AnCp^tt^_3_] structure; (b) pseudo-C_3_*z*-axis and numbering convention used for identifying C(Cp) atoms and H(Cp) atoms.

Herein, we employ Hyperion to calculate HFCCs for the two [AnCp^tt^_3_] complexes studied by Formanuik et al. (Figure 1), with the aim of assessing the performance of a fully ab initio model for HFCC calculations applied to nontrivial systems and to compare the implications of using a (non)relativistic treatment for assessing covalency in actinide compounds via spin density. We find excellent agreement between ab initio-based and experimental HYSCORE spectra for both Th and U complexes and show that while the nonrelativistic interpretation for the  Th complex is in agreement with our ab initio spin densities, the same is not true for the 5f^3^ U complex, where the original nonrelativistic interpretation significantly overestimated spin density on the ligands. Thus, the present work highlights both the accuracy of modern relativistic ab initio methods and their necessity in the interpretation of spectra and electronic properties for An compounds.

## 2 Experimental Section

Computations were performed on two different geometries: the crystal structure (XRD, reported in [9] without any adjustment), and since the experiments were performed on a solution, where the geometries will relax and hence HFCCs and *g*-values may change, we have also used density functional theory (DFT) to optimize the molecular geometries. Geometry optimization was performed using the PBE^36^ functional and Grimme’s D3 dispersion correction^37^ in Gaussian 16 Rev C.01.^38^ The Stuttgart effective *f*-in-core pseudopotentials^39^ were used for the An ions, and the all-electron cc-pVDZ basis set^40^ was used for the C and H atoms. All electronic structure calculations use the CASSCF-SO/RASSCF-SO implementation in OpenMolcas 20.10,^41^ and basis sets for all atoms are taken from the ANO-RCC library:^42−46^ TZP quality for An, DZP for C(Cp), H(Cp) and tertiary C(^*t*^Bu), and MB for all other atoms. HFCCs, *g*-values, and corresponding principal axes are determined from electronic structure data using Hyperion.^16^ Hyperfine integrals were calculated with a point nucleus model unless specified. The hyperfine integrals calculated with a Gaussian nucleus model were performed by specifying the zeta flag in Hyperion with the values of 6.8077502929 × 10^8^ for ^13^C and 2.1248239717 × 10^9^ for ^1^H.^47^ We note that our method provides eigenvalues of the symmetrized HFC tensor that corresponds to the squared principal values of the original HFC tensor, so as a result, we obtain unsigned HFCCs using the pseudospin parametrization. While Griffith proposed an effective Hamiltonian approach for obtaining the signs of components of the HFC tensor,^48^ we cannot split the spin-dependent FC and SD terms in our ab initio approach, and thus we use an approximation to determine the signs (see S2 in the SI). The *hyperion2easyspin* utility within Hyperion is then employed to generate EasySpin^49^ input files containing the computed EPR parameters, followed by HYSCORE simulations via *saffron*.^50^ Experimental and simulated HYSCORE parameters are reported in the Supporting Information. We have also performed some DFT calculations of the HFCCs for [ThCp^tt^_3_]. Here, we employed ORCA 5.0.4^51^ with the PBE density functional,^52^ the ZORA relativistic Hamiltonian (with picture change corrections),^53^ the ZORA-def2-TZVP basis set for all C and H atoms,^54^ the SARC-ZORA-TZVP basis set for Th,^55^ and the resolution of the identity approximation for the two-electron integrals using the SARC/J Coulomb-fitting basis set.^55,56^

## 3 Results and Discussion

### 3.1 Electronic Structure

Experimental *g*-values for [ThCp^tt^_3_] are *g*_∥_ = 1.974 and *g*_⊥_ = 1.880, indicating the influence of SOC with orbitally degenerate excited states as they deviate from the free electron *g*-value. However, the ground state is well-described by the  configuration and hence the orbital angular momentum of the 6d shell is mainly quenched. A state-averaged (SA) CASSCF(1,12) calculation (all 6d and 5f orbitals and accounting for all 12 doublet states) with SO coupling reveals that the ground Kramers doublet is dominated by the lowest-energy spin-free (SF) doublet state ( with 18% 5f and 5% 7s character), with small contributions from the first two excited SF doublet states, a pseudodegenerate pair with hybrid 5f/6d (35% 5f and 31% 6d character) singly occupied molecular orbitals (SOMOs), Figure S22. All subsequent SA-CASSCF-SO and SA-RASSCF-SO calculations therefore include only the lowest three SF states. The first and second excited Kramers doublets are multiconfigurational, with an approximately equal mixture of the second and third SF states; these SF states have two dominant electron configurations where each one of the hybrid 5f/6d orbitals are singly occupied. We note that the energies of the lowest SO-coupled excited states, 15,199 and 15,373 cm^–1^, are in close agreement with the first strong absorption observed in the solution-phase UV–vis spectrum (15,600 cm^–1^).^9^

To capture electron correlation effects with the cyclopentadienyl ligands, we performed RASSCF-SO calculations, where occupied and unoccupied π-type valence orbitals are included in the RAS1 and RAS3 subspaces, respectively (Table 1). In the RAS1/3 subspaces, a limit of two electron holes (RAS1) and two excitations (RAS3) is enforced to reduce computational cost. Furthermore, only the SOMOs of the 3 optimized states are kept in the RAS2 subspace, while all other unoccupied Th orbitals are assigned to RAS3. The complex chemistry of actinides is a consequence of nontrivial bonding modes involving some or all of the 5f, 6d, 7s and 7p orbitals, and therefore we have designed the RASSCF active spaces to include orbitals with significant contributions from these atomic orbitals; herein, the molecular orbitals (MOs) are labeled with the majority atomic orbital component. As we are interested in modeling HFC, we also investigate the effects of correlating outer core electrons using a larger SA-RASSCF(27,36)-SO wave function (Table 1); we note that this active space gives the most accurate *g*_⊥_, while SA-RASSCF(19,27)-SO gives the most accurate *g*_∥_ (Table 2); nevertheless, all *g*-values calculated from the crystal structure are in good agreement with experiment. The *g*-values computed using the DFT-optimized structure provide similar results as the computations performed on the XRD structure (Table 2). The XRD structure is C_1_ [root-mean-squared deviation (RMSD) of 0.23 Å from C_s_], and the optimized structure is pseudo-C_3h_ (RMSD of 0.004 Å from C_3h_), and we do see slightly more symmetric *g*_⊥_ values for the latter structure.

**Table 1:** [ThCp^tt^_3_] Active Orbitals Included in State-Averaged CASSCF-SO/RASSCF-SO with 3 Spin Doublet Roots and Single-State RASSCF Calculations^a^

active space	RAS1	RAS2	RAS3
SA-CASSCF(1,12)-SO		5 × 6d, 7 × 5f	
SA-RASSCF(19,27)-SO	9 × π_Cp_	3 × 6d	6 × π_Cp*_, 2 × 6d, 4 × 5f, 7s, 7p_*x*,*y*_
SA-RASSCF(27,36)-SO	6s, 3 × 6p, 9 × π_Cp_	3 × 6d	6 × π_Cp*_, 2 × 6d, 7 × 5f, 7s, 3 × 7p, 8s
SS-RASSCF(19,26)	9 × π_Cp_	6d_*z*_^2^	6 × π_Cp*_, 4 × 6d, 2 × 5f, 7s, 3 × 7p
SS-RASSCF(27,36)	6s,3 × 6p, 9 × π_Cp_	6d_*z*_^2^	6 × π_Cp*_, 4 × 6d, 7 × 5f, 7s, 3 × 7p, 8s
SS-RASSCF(39,38)	6 × σ_Cp_, 6s, 3 × 6p, 9 × π_Cp_	6d_*z*_^2^	6 × π_Cp*_, 4 × 6d, 7s, 7pz, 6 × σ_Cp*_

a Active orbital figures can be found in the Supporting Information.

**Table 2:** [ThCp^tt^_3_] *g*-Values Calculated with Computational Methods, Compared to Experimental Values Obtained from Continuous Wave EPR Spectroscopy^a^

	XRD			optimized		
active space	*g*_∥_	*g*_⊥_	*g*_⊥_	*g*_∥_	*g*_⊥_	*g*_⊥_
SA-CASSCF(1,12)-SO	1.975	1.827	1.821	1.969	1.743	1.742
SA-RASSCF(19,27)-SO	1.973	1.831	1.826	1.973	1.828	1.827
SA-RASSCF(27,36)-SO	1.983	1.870	1.866	1.982	1.867	1.867
DFT	2.124	1.981	1.838	2.030	1.888	1.874
experiment	1.974	1.880	1.880			

a Note: Owing to the imperfect symmetries of the structures (XRD - C_1_, optimized - pseudo-C_3h_), the two *g*_⊥_ values are not coincident.

The accuracy of theoretical HFCCs can be significantly impacted by the quality of optimized MOs.^16^ State-averaged calculations of [ThCp^tt^_3_] include a ground state with an axial spin density distribution ( SOMO) with 2 highly excited states at ca. 15,300 cm^–1^ having in-plane spin density distributions (Figure S22). Hence, it is possible that the state-averaged MOs for ground-state wave function obtained with this state-average calculation do not provide an adequate description of ligand HFC in the ground state. In order to control for this possibility, we also carry out state-specific (SS) RASSCF optimizations of the ground doublet for [ThCp^tt^_3_] (Table 1) and determine ground-state HFCCs using a spin-only parametrization. [Note that because there is only one doublet root, the SO coupling would have no effect, and the results are identical with and without SO coupling.] Taking advantage of the larger RAS1 and RAS3 selections available due to the one-orbital RAS2 subspace, we additionally correlate σ_Cp_ and σ_Cp*_ type orbitals in SS-RASSCF(39,38), therefore allowing for spin density delocalization over C(Cp)-C(Cp), C(Cp)-C(^*t*^Bu), and C(Cp)-H(Cp) σ bonds. Furthermore, given the large energy gap to the lowest-energy excitations and the fact that the ground state of [ThCp^tt^_3_] is well-described as a single determinant, we have also performed DFT calculations of the *g*-values and HFCCs, which will be discussed in comparison to the multiconfigurational results below. However, we can already observe that while the sense of the magnetic anisotropy in [ThCp^tt^_3_] is captured by the DFT calculations (i.e., *g*_∥_ > *g*_⊥_), the *g*_∥_ value is larger than the free-electron *g*-value of 2.0023..., which is not in agreement with the experiment or the multiconfigurational results.

U(III) has an idealized 5f^3^ configuration associated with a ^4^I_9/2_ Russel-Saunders ground term. In [UCp^tt^_3_], the ^4^I_9/2_ term is split by the ligand field giving a Kramers doublet ground state which can be modeled as an effective . CASSCF and RASSCF calculations of [UCp^tt^_3_] must include the seven 5f orbitals in the RAS2 space, and herein we consider the lowest 13 quartets and 11 doublets corresponding to the lowest lying terms for each multiplicity (^4^I and ^2^H; Table 3). It is immediately apparent (Table 4) that the minimal SA-CASSCF(3,7)-SO using the XRD structure does not capture the magnitude and rhombicity of the experimental *g*-values (*g*_*x*_ = 3.645, *g*_*y*_ = 2.563, *g*_*z*_ < 0.5),^9^ and increasing the number of optimized states has no effect on the accuracy of these results. Significant improvement is obtained upon correlating π_Cp_-type orbitals with a SA-RASSCF(21,30)-SO calculation.

**Table 3:** [UCp^tt^_3_] Active Orbitals Included in State-Averaged CASSCF-SO/RASSCF-SO with 13 Spin Quartet Roots and 11 Spin Doublet Roots^a^

active space	RAS1	RAS2	RAS3
SA-CASSCF(3,7)		7 × 5f	
SA-RASSCF(21,30)	9 × π_Cp_	7 × 5f	6 × π_Cp*_, 4 × 6d, 7s, 3 × 7p
SA-RASCI(29,34)	6s, 3 × 6p, 9 × π_Cp_	7 × 5f	6 × π_Cp*_, 4 × 6d, 7s, 3 × 7p
SA-RASCI(29,35)	6s, 3 × 6p, 9 × π_Cp_	7 × 5f	6 × π_Cp*_, 4 × 6d, 7s, 3 × 7p, 8s

a Active orbital figures can be found in the Supporting Information

**Table 4:** [UCp^tt^_3_] *g*-Values Calculated with Multiconfigurational Methods, Compared to Experimental Values

	XRD			optimized		
active space	*g*_*x*_	*g*_*y*_	*g*_*z*_	*g*_*x*_	*g*_*y*_	*g*_*z*_
SA-CASSCF(3,7)-SO	2.524	0.301	0.031	3.038	2.708	0.061
SA-RASSCF(21,30)-SO	4.117	1.860	0.357	3.135	2.952	0.356
RASCI(29,34)-SO	4.020	2.241	0.403	3.099	2.940	0.336
RASCI(29,35)-SO	4.014	2.192	0.388	3.089	2.930	0.321
experiment	3.645^a^	2.563^a^	<0.5^a^	3.050^b^	1.650^b^	<0.5^b^

a Crystal structure.

b Frozen solution.

The cost of RASSCF calculations scales more steeply for [UCp^tt^_3_] compared with [ThCp^tt^_3_], owing to the larger RAS2 subspace. As a result, larger active spaces become unfeasible and, in order to correlate more electrons, we resort to a RASCI-SO approach (where the orbitals are not optimized) using orbitals obtained from SA-RASSCF(21,30) calculations. Upon augmenting the SA-RASSCF(21,30)-SO space with U 6s and 6p [RASCI(29,34)-SO] and U 8*s* [RASCI(29,35)-SO], the *g*_*y*_ value is significantly improved using the XRD geometry (Table 4), indicating that the U outer core region influences in-plane magnetization, just as observed for [ThCp^tt^_3_]. Using the optimized geometry of [UCp^tt^_3_], even calculations with a minimal active space produces *g*_*x*_ and *g*_*y*_ values similar to the frozen solution experimental values, but they do not improve upon increasing the active space. The structural differences between the XRD and DFT-optimized geometry for [UCp^tt^_3_] are much larger than what was seen with [ThCp^tt^_3_]. For the XRD structure of [UCp^tt^_3_], it has an RMSD of 0.34 Å from C_s_ (and 0.41 Å from C_3h_), meaning it is the least-symmetric geometry studied here. The optimized structure has an RMSD of only 0.04 Å from C_3_, which may be more reflective of the average structure in solution, which may be why there is less variation in *g*-values for different wave functions for this geometry. However, we note that for subsequent HYSCORE simulations, we use the experimental *g*-values in order to compare with the experimental spectra.

### 3.2 Hyperfine Coupling

Accurate HFC calculations require an appropriate treatment of core spin polarization, of which can be approximated by computing increasingly large active space wave functions that include many electron configurations and excitations. The largest active spaces for the systems of interest are SS-RASSCF(39,38) for [ThCp^tt^_3_] and RASCI(29,35)-SO for [UCp^tt^_3_]. It is of interest to determine where approximations can be made, such as using smaller active spaces, without sacrificing accuracy. Using the largest active space calculations as a reference, we report the RMSD of the C and H hyperfine tensors in Table 5 for all calculations discussed in Section 3.1; raw HFCCs can be found in the Supporting Information, Tables S4–S18.

**Table 5:** RMSD in MHz of Calculated Hyperfine Tensors with Respect to the Largest Active Spaces Used to Compute the Electronic Structures of [ThCp^tt^_3_] [SS-RASSCF-(39,38)] and for [UCp^tt^_3_] [RASCI(29,35)-SO]

[ThCp^tt^_3_]	H	C	all
SA-CASSCF(1,12)-SO	0.035	0.213	0.137
SS-RASSCF(19,26)	0.031	0.238	0.115
SA-RASSCF(19,27)-SO	0.182	0.284	0.220
SS-RASSCF(27,36)	0.018	0.127	0.061
SA-RASSCF(27,36)-SO	0.162	0.241	0.191

[UCp^tt^_3_]	H		
SA-CASSCF(3,7)-SO	1.141		
SA-RASSCF(21,30)-SO	0.533		
RASCI(29,34)-SO	0.056		

For both systems, the RMSD generally decreases as the active space size increases. In the case of [ThCp^tt^_3_], where both state-averaged and single-state type wave functions were used, the RMSD tends to be slightly higher for the state-averaged wave functions (likely due to using a single-state wave function as a reference), but the RMSD is small overall for [ThCp^tt^_3_]. For [UCp^tt^_3_], on the other hand, there is clearly a significant decrease in accuracy when using only the minimal active space, while the other active spaces are good approximations to the largest calculation. A reasonable compromise between computational cost and accuracy must be met, and the RMSD results suggest that the accuracy gained from using very large active spaces to calculate HFCCs is small for the [AnCp^tt^_3_] complexes studied here.

Given that [ThCp^tt^_3_] is well-approximated as a single-configuration ground state, we have also performed DFT calculations of the HFCCs (see Experimental) to compare them with our multiconfigurational calculations with Hyperion. Comparing the DFT-calculated HFCCs to the SA-RASSCF(19,27)-SO results, we find a good correlation between the two methods (*R*^2^ between 0.88 and 0.94) for the ^13^C HFCCs; however, there is a systematic overprediction of the DFT-calculated values by about a factor of 2.2–2.5 (Figures S28, S27, S24, S23). For ^1^H, the H_A_ protons on the Cp ring and the Me protons that sit near the *C*_*3*_ axis are outliers in the correlation of *A*_iso_ between DFT and CASSCF for the optimized structure (all other protons show a good correlation with linear regression slope of 2.0 with average *R*^2^ = 0.97; Figure S26), and for the experimental crystal structure, the Me protons that sit near the *C*_3_ axis have *A*_iso_ values that correlate but are underpredicted by DFT (linear regression slope of 0.76 with average *R*^2^ = 0.98; Figure S30). Interestingly, for all protons and in both structures, the Euclidean norm of the HFCCs ( where *A*_*i*,*n*_ are the eigenvalues of ***A***_*i*_) is in very good agreement between DFT and CASSCF (Figures S25 and S29; average linear regression slope of 1.1 with average *R*^2^ = 0.98), bringing these H_A_ and C_3_-proximal Me protons back into line. The different behavior of the C_3_-proximal Me protons is due to the overestimated *g*_∥_ value, which exaggerates the anisotropy (Table S23), and the fact that these protons are very sensitive due to their near on-axis position. The different behavior of the HFCCs for the H_A_ protons is not due to the anisotropy in the HFCCs, but rather simply due to a different *A*_iso_ component: DFT predicts *A*_iso_ ≈ – 1 MHz, while CASSCF predicts *A*_iso_ ≈ 0 MHz (Table S24). Given the excellent agreement between experimental and CASSCF *g*-values and HYSCORE plots (see below), we propose that the PBE-ZORA DFT calculations are not as accurate as those of CASSCF in this case.

All of the calculations in this manuscript so far have been performed with a point nucleus model; however, it is known that it may be important to use a finite nucleus model for contributions to the FC term.^57^ A set of comparison calculations was performed for [ThCp^tt^_3_] and for [UCp^tt^_3_] with the SA(19,27)-SO and SA(21,30)-SO wave functions, respectively, by using the Gaussian nucleus model^47,57^ option in Hyperion. The RMSD of the Gaussian nuclei calculations in comparison to the point charge model is < 0.00 MHz for [ThCp^tt^_3_] and 1.80 MHz for [UCp^tt^_3_] (Tables S19 and S20). These are similar to the RMSDs found when changing the active space size: a small difference for [ThCp^tt^_3_] and a larger difference for [UCp^tt^_3_], but overall, they are very small, suggesting a minor influence of a Gaussian nucleus model.

### 3.3 HYSCORE Simulations

The interpretation of HYSCORE spectra is complex, hence the need for simulation and reference calculations, such as in this work. In general terms, the upper right quadrant (+,+; which we exclusively discuss herein) is the weak-coupling region where the diagonal position of the feature corresponds to the Larmor frequency of the nuclear spin, and the separation of cross-peaks corresponds to the HFCC. Anisotropy of the hyperfine interaction results in ridges, rather than islands, when multiple orientations are excited. We recommend the review by van Doorslaer for an introduction to the practicalities of the technique.^58^ Here, we focus on simulations of ^1^H and ^13^C HYSCORE spectra from SS-RASSCF(19,26) and SA-RASSCF(19,27)-SO ([ThCp^tt^_3_]) and SA-RASSCF(21,30)-SO ([UCp^tt^_3_]) calculations; HYSCORE simulations from other active spaces can be found in the Supporting Information. Due to the steep scaling of pulsed EPR simulations,^50^ it is necessary to limit the number of magnetic nuclei included in the HYSCORE simulations. We choose sets of representative nuclei based on the magnitude (Euclidean norm) of the HFC tensors and distance to the An^3+^ atom, along with considering symmetry arguments (axiality of [ThCp^tt^_3_] *g*-values, pseudomirror plane bisecting the cyclopentadienyl plane). Furthermore, as hyperfine values are extremely sensitive to any structural changes (HFCC relates to the cubed inverse of the distance between the nuclear and electron spins; see Section S1 in the SI), we also observe slightly different HFCCs and hence simulated spectra for different geometries (see Supporting Information).

#### 3.3.1 [ThCp^tt^_3_]

**Figure 2:**
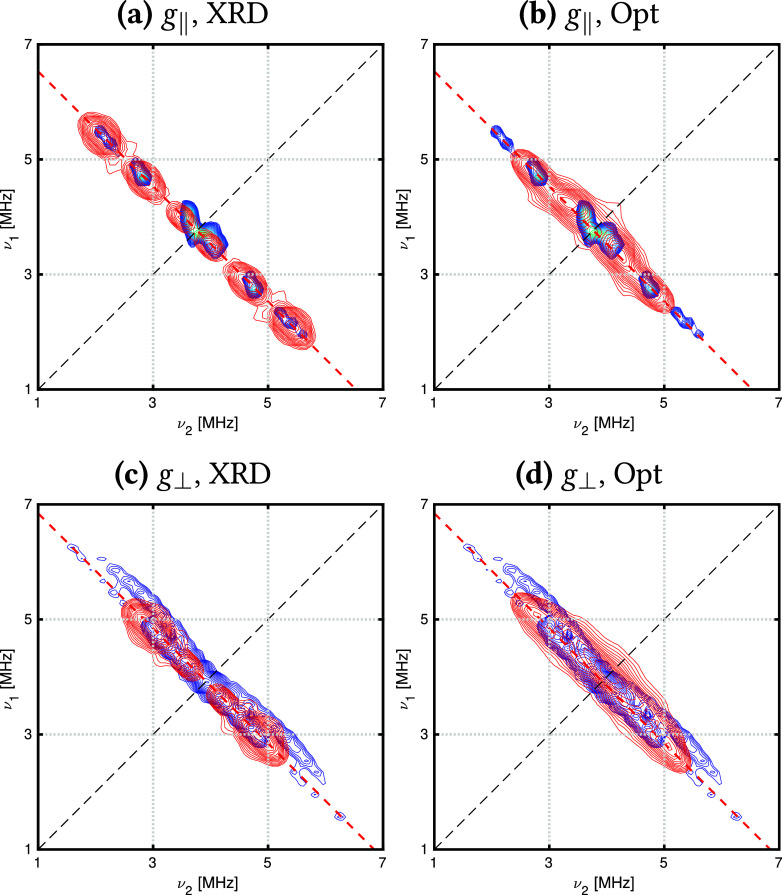
Experimental (blue) and simulated (red) HYSCORE spectra for *B*_0_ = 366.3 mT (*g*_∥_, left) and *B*_0_ = 351.6 mT (*g*_⊥_, right) in the ^13^C region for [ThCp^tt^_3_]. Simulations use parameters calculated from SA-RASSCF(19,27)-SO. All simulations include five C(Cp) atoms from one ligand, two 1° C(^*t*^Bu), and two 3° C(^*t*^Bu) nuclei; these latter four nuclei are from the same ligand in the optimized geometry and the closest ones to Th for the XRD geometry.

Based on the magnitude of the calculated ^13^C HFCCs, we use a representative set of nine ^13^C nuclei, comprising five C(Cp) nuclei, two 3° C(^*t*^Bu), and two 1° C(^*t*^Bu) nuclei [all from the same ligand for the optimized structure, and the closest 3° and 1° C(^*t*^Bu) nuclei to Th from the XRD structure] to simulate the HYSCORE spectra for *g*_∥_ and *g*_⊥_ orientations (Figure 2).

State-averaged calculations tend to give the best simulations of the ^13^C spectra, in particular SA-RASSCF(19,27)-SO, which somewhat reproduces the anisotropy–encoded in the curvature of the ridges–of the *g*_⊥_ data set (Figure 2d) when using the optimized geometry, and the separated signals in the *g*_∥_ spectrum when using the XRD structure (Figure 2a); we note that these separated features were not reproduced in the original work by Formanuik et al. using a simpler model based only on nuclei of the Cp rings, demonstrating the power of an ab initio approach. Larger state-averaged calculations, for instance, SA-RASSCF(27,36)-SO, yield HFCCs of the correct magnitude–encoded in the length of the ridges–however the simulated ridges are less curved (Figure S32). Different signals are seen for the optimized and XRD structures, indicating how sensitive HFC is to the molecular structure; however, between the two simulations, they capture most features of the ^13^C spectra. It is important to note that the experimental data are collected for a frozen solution and hence will contain many different molecular conformations, of which the XRD and optimized geometry are likely members, but still our calculations are only snapshots of an ensemble.

**Figure 3:**
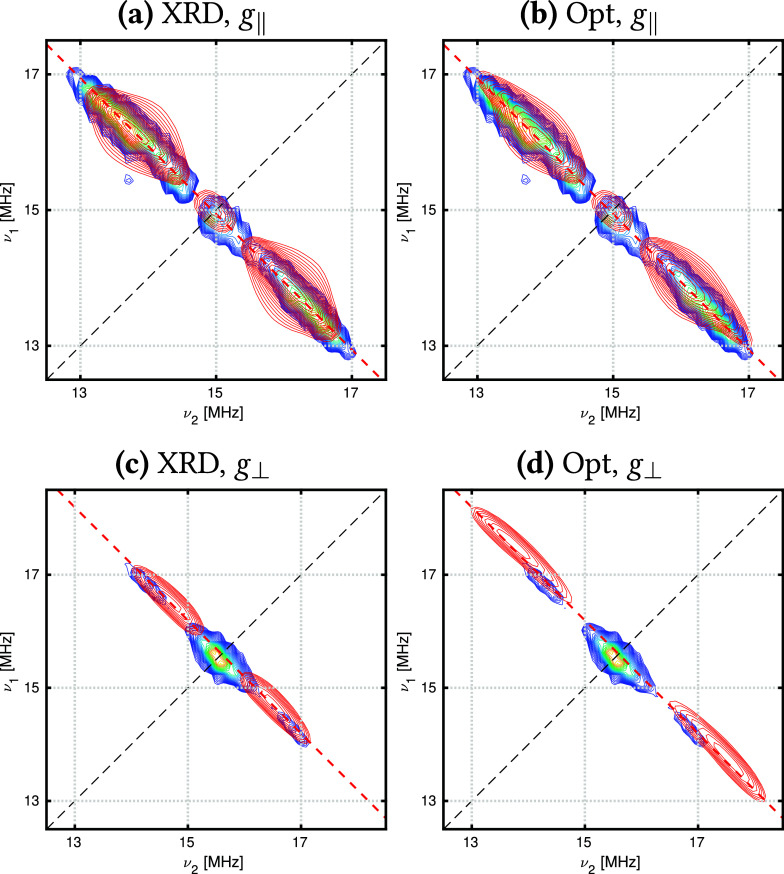
Experimental (blue) and simulated (red) HYSCORE spectra for *B*_0_ = 366.3 mT (*g*_∥_, left) and *B*_0_ = 351.6 mT (*g*_⊥_, right) in the ^1^H region for [ThCp^tt^_3_]. Simulations for both *g*_∥_ and *g*_⊥_ use EPR parameters calculated from SS-RASSCF(19,26). All simulations of the optimized structure include all nine H(Cp) nuclei and three H(^*t*^Bu) nuclei; XRD simulations include four nuclei: three H(Cp) from one ligand and one H(^*t*^Bu) nuclei, labeled H8 in the XRD structure.

^1^H HYSCORE simulations using the optimized structure of [ThCp^tt^_3_] employ 12 ^1^H nuclei, of which nine are H(Cp) atoms from the cyclopentadienyl rings and the remaining are representative H(^*t*^Bu) nuclei, one from each ligand. The best simulation using the XRD structure contained only four H nuclei: three H(Cp) from one ligand and one representative H(^*t*^Bu) nucleus. We note that including the closest H(^*t*^Bu) from the XRD structure in the simulation can produce extremely intense features (Figure S31), but it still produces signals in the correct positions. Using nuclei (labeled H8 in the XRD structure) that have spin population closer to average H(^*t*^Bu) values give less intense signals; these nuclei are selected in the simulations within the main manuscript (Figure 3).

The simulated *g*_∥_ spectra show that using the XRD structure produces a more intense signal in the regions corresponding to larger HFCCs (out to 17 MHz), which can be attributed to the slight difference in geometry compared to the optimized structure. The central feature at *g*_⊥_, corresponding to near-zero HFCCs, is likely caused by weakly coupled nuclei, such as those of solvent molecules; the features arising from more strongly coupled nuclei are correctly represented by our simulations.

**Figure 4:**
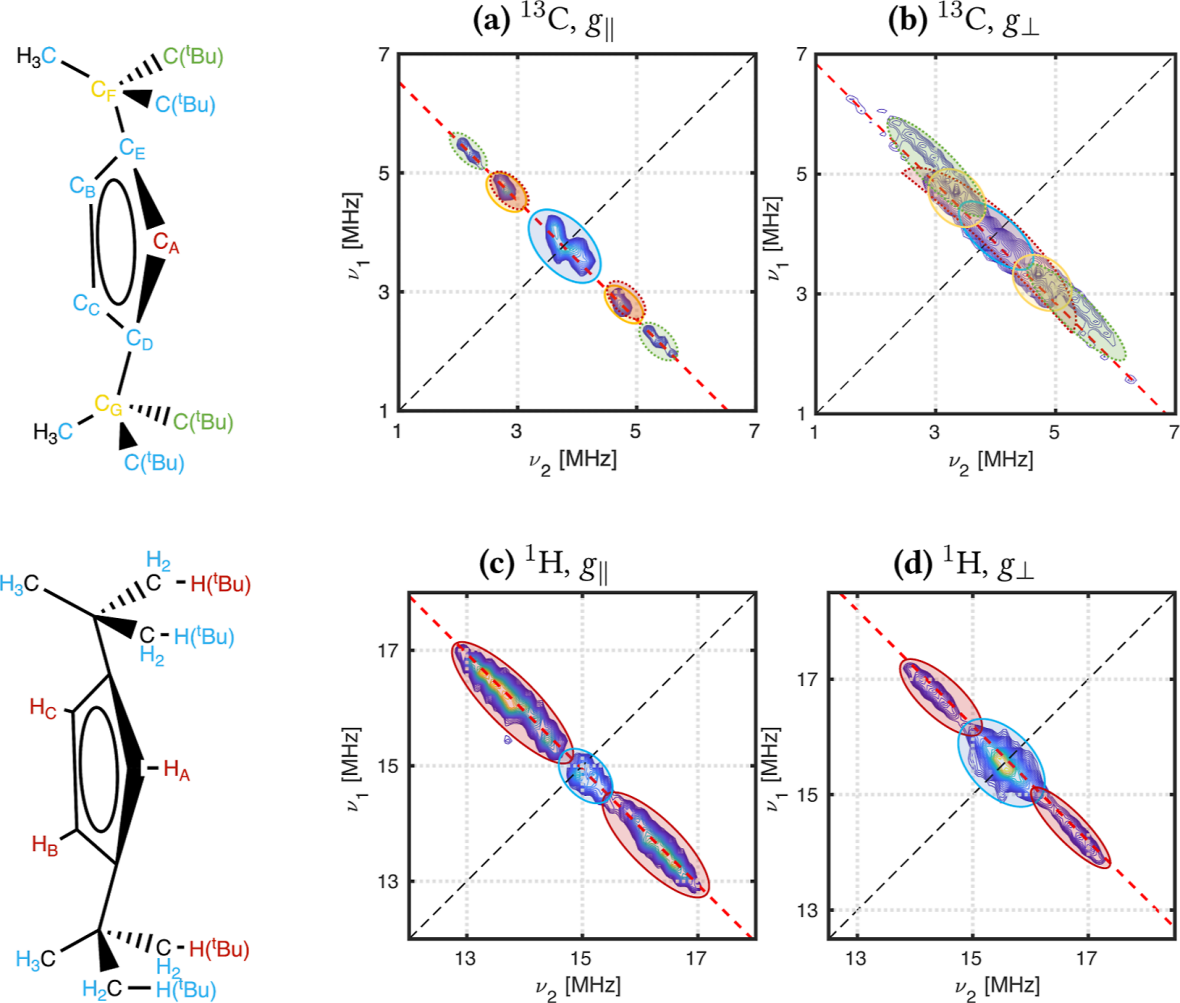
Assignment of ^13^C and ^1^H HYSCORE spectra of [ThCp^tt^_3_].

Assignment of the ^13^C and ^1^H HYSCORE spectra of [ThCp^tt^_3_] can then be performed by including different subsets of nuclei during simulation of the spectra. This protocol revealed that the ^13^C ridges observed in the *g*_∥_ spectrum at ν_N_ ± 0.5 MHz, ν_N_ ± 1 MHz, and ν_N_ ± 1.5 MHz (where ν_N_ is the nuclear Larmor frequency) are due to C(Cp), 3° C(^*t*^Bu), and 1° C(^*t*^Bu) nuclei, respectively (Figure 4a).

The original report of Formanuik et al. assigned the two distinct features in the *g*_⊥_^13^C spectrum, one with a spread of ν_N_ ± 1 MHz and the second a longer, arched signal with a spread of ν_N_ ± 2.4 MHz as C_B_/C_C_ and C_A_, respectively. Our methods provide a more complete assignment of these features (Figure 4): the most strongly coupled nuclei are those that sit above and below the Th(III) ion [C(^*t*^Bu), closest to the *C*_*3*_ axis], followed by C_A_ and C_F_/C_G_. This ordering occurs because the ground-state spin density takes the shape of the 6d_z_^2^ orbital (Figure S21), where the lobes more closely interact with these nuclei. Spectra simulated using individual sets of nuclei show diffuse and anisotropic signals in similar regions in the *g*_⊥_ direction for both 1° C(^*t*^Bu) and 1° C_A_, making it difficult to discern which signal is responsible for the longer, arched signal in the experimental spectrum (Figure S37). Additionally, the simulated ^13^C spectrum contains overlapping signals for C_A_ and the 3° C(^*t*^Bu) groups (C_F_ and C_G_), demonstrating the importance of including these nuclei in the simulation but also the benefit of using ab initio methods to aid in the assignment of spectra, where the delocalized spin density distribution is obtained directly from our calculations.

#### 3.3.2 [UCp^tt^_3_]

**Figure 5:**
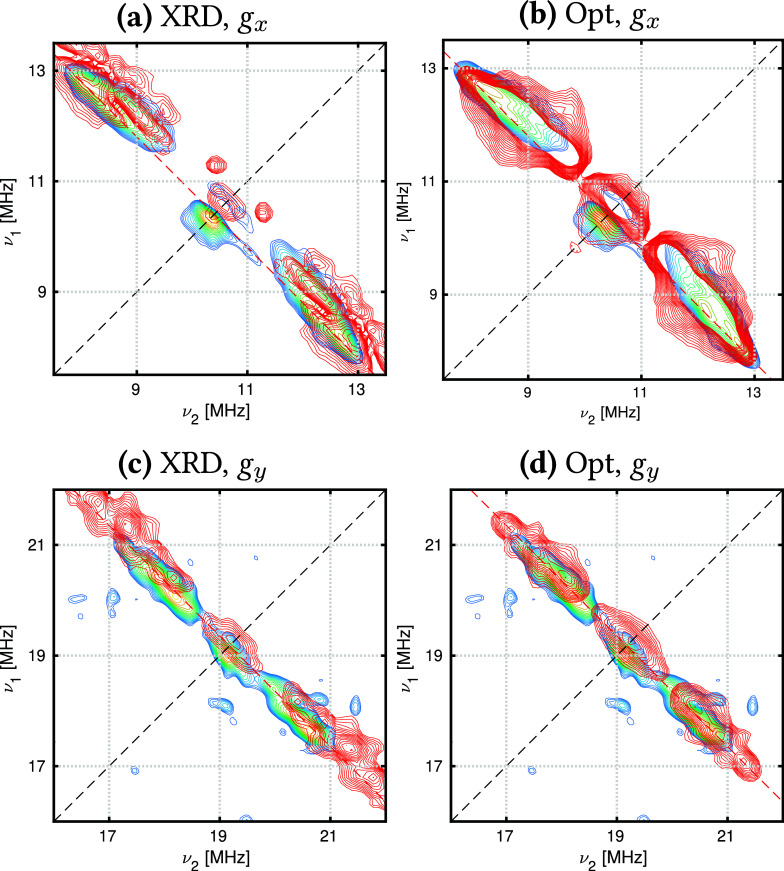
Experimental (blue) and simulated (red) HYSCORE spectra for *B*_0_ = 244.3 mT (*g*_*x*_, left) and *B*_0_ = 450.4 mT (*g*_*y*_, right) in the ^1^H region for [UCp^tt^_3_]. Simulations for the XRD structures (a,c) include 12 nuclei: nine H(Cp) and the three closest H(^*t*^Bu), and the Opt structures (b,d) include nine H(Cp) nuclei. Simulations used HFCCs computed with SA-RASSCF(21,30)-SO.

The rhombicity of the [UCp^tt^_3_] *g*-values requires nuclei from all Cp^tt^ groups to be included in ^1^H HYSCORE simulations. This is apparent in simulations using the XRD structure, as including nuclei from only one ligand gives different results compared with simulations using a different ligand (Figures S39 and S40). Hence, all nine H(Cp) nuclei are used to simulate the ^1^H HYSCORE spectra for both the XRD and optimized structures of [UCp^tt^_3_] (Figure 5).

**Figure 6:**
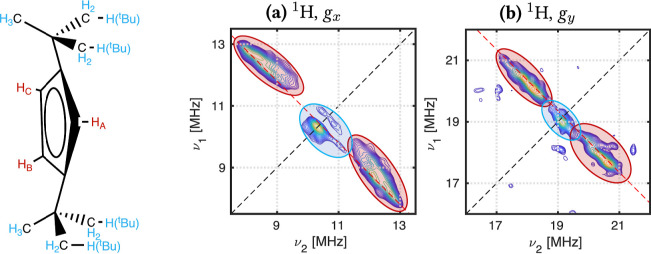
Assignment of ^1^H HYSCORE spectra of [UCp^tt^_3_].

Simulations computed with HFCCs from the XRD structure capture all features present in the experimental spectra; however, the largest HFCCs in the *g*_*y*_ spectrum are slightly overestimated. Simulations using HFCCs obtained from the optimized structure give more uniform signals that more closely match the experimental spectra, and good simulations are even produced with a minimal active space (Figure S41, right). For the XRD structure, larger active spaces more accurately model all regions of the spectrum in comparison to minimal active space simulations (Figure S41, left). Assignment of the [UCp^tt^_3_] ^1^H HYSCORE spectra (Figure 6) shows that the most strongly coupled nuclei are H(Cp). The ground-state spin density of [UCp^tt^_3_] is roughly spherical (Figure S21), and therefore the H(^*t*^Bu) nuclei only contribute to the weakly coupled region in the center as they are much further from the unpaired spin density; this assignment agrees with the previous study.

### 3.4 Quantifying [AnCp^tt^_3_] Covalency

We use Mulliken spin population analysis (Section S10) to quantify spin delocalization across ligand atoms and thus the degree of covalency in the [AnCp^tt^_3_] complexes. For the XRD structure of [ThCp^tt^_3_], a SS-RASSCF(39,38) wave function results in Mulliken spin populations of 0.0044, 0.0089, 0.0040, −0.0003, and −0.0005 for nuclei C_E_, C_A_, C_D_, C_C_, and C_B_, respectively, and 0.0011, −0.0014, and −0.0020 for H_A_, H_B_, and H_C_, respectively. Note that all electronic structure calculations for [ThCp^tt^_3_], other than the minimal CASSCF(1,12)-SO, yield C(Cp) spin populations with a clear C_A_ > C_D_/C_E_ > C_B_/C_C_ trend. The spin population is extremely small on both the 1° and 3 °C(^*t*^Bu) nuclei, which are all within −0.0005 to 0.0004, and yet, these nuclei show the largest ^13^C HFCs; this implies that the HFC is dominated by the dipolar interaction. This conclusion becomes clear once you consider the 6d_z_^2^ shape of the ground-state spin density (Figure S21), which brings the unpaired spin density close to that of the ^*t*^Bu nuclei. This is a considerable departure from the original point-dipole approximation employed by Formanuik et al., but despite the differences between the two models, the calculated Mulliken spin populations are close to the originally estimated values: 0.010 for C_A_ and 0.002 for C_D_/C_E_, while the C_A_ spin population of 0.014, determined from ^1^H data, was slightly overestimated.

Spin population analysis of [UCp^tt^_3_] is complicated by extensive mixing between SF states due to strong SOC (Table S25). Herein, we focus on Mulliken spin populations derived for the fourth quartet SF state, which has the largest contribution to the ground Kramers doublet. The spin populations for H(Cp) range between −0.0001 and −0.0004, regardless of active space or state; this is 2 orders of magnitude lower than the value of 0.019 reported by Formanuik et al.^9^ Given the good reproduction of the experimental HYSCORE spectra based on our calculations, this discrepancy highlights the shortcomings of using nonrelativistic HFC models in the case of strongly SO-coupled systems: large HFCCs had to be modeled by increasing spin delocalization when the relativistic PSO term was not included in the model. This is especially pronounced in [UCp^tt^_3_] where the average contribution of the PSO term to the overall HFC is 68%, whereas for [ThCp^tt^_3_], this contribution lies between 5 and 11% for the different active spaces used here (Table S29). Indeed similar conclusions were drawn by Sergentu et al. in their study of the [U(C_7_H_7_)_2_]^1–^ complex, where the PSO term inverts the sign of *A*_iso_ expected based on the ^1^H spin densities.^59^

Although no HYSCORE signal could be measured in the ^13^C region for [UCp^tt^_3_],^9^ C(Cp) spin populations from electronic structure calculations provide a useful point of comparison with [ThCp^tt^_3_]. The C(Cp) spin populations derived from RASCI(29,35)-SO fall between −0.0023 and −0.0038 for C_A_, C_D_, and C_E_; these values are in stark contrast with the positive populations of the equivalent atoms in [ThCp^tt^_3_] and indeed are consistent with the DFT spin-density iso-surface for [UCp^tt^_3_] reported by Formanuik et al. and that obtained here (Figure S21b). Smaller spin populations, ranging from −0.0004 to −0.0014, are observed for C_B_- and C_C_-type nuclei, in line with the Th complex. It appears that, relative to a purely ionic picture, spin density transfer between Cp^tt^ and An occurs primarily via atoms at the C_A_, C_D_, and C_E_ positions for both complexes. Considering also the Mulliken spin populations at An, 0.96 for Th and 3.03 for U, we deduce that the Cp^tt^-An interaction leads to spin density transfer away from Th(III) in [ThCp^tt^_3_] and to spin density transfer toward U(III) in [UCp^tt^_3_].

Orbital decomposition analysis of the HFC matrices (Section S12) reveals similar patterns of pairwise orbital contributions (which are proportional to the probability of spin density transfer between orbitals), consistent with our observations from Mulliken spin population analysis. For [ThCp^tt^_3_], C(Cp) and H(Cp) HFC matrices appear to be dominated by Cp π_2_ → Cp π_3_^*^ and Cp π_2_ → Th 6d excitations. Herein, we have used π_1_, π_2_, and π_3_^*^ to label the valence π-type MOs of the cyclopentadienyl groups based on the number of nodal planes, which, according to MO theory, determines the MO energy; although our electronic structure approach is more complex than basic MO theory, this is a useful strategy to classify the 9 bonding π_Cp_ MOs (3 × π_1_ and 6 × π_2_) and the 6 antibonding π_Cp*_ MOs (all π_3_^*^). Some Cp π_1_ → Cp π_3_^*^ and Th 6p_*x*,*y*_ → Cp π_3_^*^ contributions are observed for the in-plane atoms (C_A_, Figure S43, and H_A_, Figure S45); however, as our calculations yield strongly hybridized Th 6p_*x*,*y*_–Cp π_1_ MOs, both types of contributions are likely due to interactions between ligand atoms. The inclusion of σ_Cp_ and σ_Cp*_ MOs in SS-RASSCF(39,38) has a significant impact, with σ_Cp_ → σ_Cp*_ excitations manifesting as strong contributions to the HFC matrices of C_D_, C_A_, and H_A_ (Figures S42, S43 and S45, respectively). The orbital decomposition diagrams for C(Cp) and H(Cp) in [UCp^tt^_3_] are overall more sparse than their [ThCp^tt^_3_] equivalents, particularly in the case of C(Cp). Metal–ligand spin density transfer appears to occur mainly via Cp π_2_ → U 6d excitations. Unlike the Th complex, we observe strong U 6p_*x*,*y*_ → 7p_*x*,*y*_ pairwise contributions in the orbital decomposition for C_D_, C_A_, and H_A_, i.e., the positions in the cyclopentadienyl ring associated with the largest spin populations.

## 4 Conclusions

Here, we have employed multiconfigurational active space electronic structure techniques, together with the recently developed Hyperion program, to derive relativistic HFC parameters for two [AnCp^tt^_3_] complexes, which were previously characterized by Formanuik et al. using pulsed EPR techniques. Simulated HYSCORE spectra based on the calculated parameters were then used to benchmark our electronic structure calculations against experiment and to improve upon the original assignment of the spectra and correct misinterpretations based on simpler models. Overall, we have shown that good predictions of ligand HFCCs can be obtained from a fairly minimal relativistic electronic structure model. In particular, HYSCORE simulations of [ThCp^tt^_3_] uncovered the strong influence of the ^*t*^Bu nuclei on both ^13^C and ^1^H spectra. Additionally, a nonrelativistic interpretation of HFC becomes unreliable where the PSO term dominates, such as for [UCp^tt^_3_] in this work.

To gain insights into An-ligand bonding and the extent of metal–ligand covalency, we employed Mulliken spin population analysis, which showed that the two [AnCp^tt^_3_] complexes differ in the direction of spin density transfer between An and the Cp^tt^ ligands. The extent of spin delocalization appears to be diminished for the U complex, with only C(Cp) atoms having spin populations significantly different from zero. By contrast, spin polarization in [ThCp^tt^_3_] extends as far as H(Cp), for which spin populations are the same order of magnitude as C(Cp) spin populations. Although they are not a direct measure of An covalency, the effects on H(Cp) show that spin populations beyond the first coordination sphere can be affected by the nature of the metal. Mulliken spin populations on the An atoms suggest that actinide covalency is slightly stronger in the Th complex, which is the opposite conclusion to the study by Formanuik et al.; while that study included orbital effects in the dipolar contribution, the contribution of the PSO term was not included.

Thus, despite good agreements to experimental spectra obtained in the original study by Formanuik et al. using simple models, our present work indicates that many potential similar models may also provide good agreement to experiment, and hence that high-quality calculations, such as the present fully *ab initio* relativistic approach, are required to unambiguously assign actinide HYSCORE spectra. This is an important milestone as we attempt to enhance our understanding of actinide covalency via experimental techniques.

## Supporting Information Available

Theoretical sketch, method for determining signs of HFCC, figures showing orientations of principal directions of *g*-matrices, spin density plots, computed HFCC values, additional HYSCORE simulations, SO states for [UCp^tt^_3_], Mulliken spin population analysis, HFCC orbital decomposition, and PSO contribution to HFCC (PDF)

## Notes

Research data supporting this publication is available at DOI: 10.48420/25621644.
