# Supplementary material for: Relativistic Quantum Chemical Investigation of Actinide Covalency Measured by Electron Paramagnetic Resonance Spectroscopy
Source: J Am Chem Soc. 2024 May 16;146(21):14660–71. doi: 10.1021/jacs.4c01930 (PMC11140756; doi:10.1021/jacs.4c01930)
Supplement: Supplementary file 1 — ja4c01930_si_001.pdf [file ja4c01930_si_001.pdf]

Supplementary Information for: A Relativistic Quantum Chemical Investigation of Actinide Covalency Measured by EPR Spectroscopy

Letitia Birnoschi[1,†], Meagan S. Oakley[1,†], Eric J. L. McInnes[1], and Nicholas F. Chilton*[1,2]

[1]Department of Chemistry, The University of Manchester, Oxford Road, Manchester, M13 9PL, United Kingdom

[2]Research School of Chemistry, The Australian National University, Sullivans Creek Road, Canberra, ACT 2601, Australia; email: nicholas.chilton@anu.edu.au

[†]These authors contributed equally.

S1 Theoretical sketch

A full account of the theory can be found elsewhere,[1] however here we provide a brief sketch. Starting from the field-free 4-component Dirac equation, relativistic expressions for the Zeeman and the HFC operator can be determined by adding vector potentials arising from external and nuclear magnetic fields, respectively, as first-order perturbations. This 4-component model is then simplified via scalar-relativistic eXact-2-Component (SR-X2C) decoupling,[2–15] wherein a unitary transformation, known as the picture-change transformation, is applied to the zeroth order Hamiltonian and to the perturbation operators. As a result, the relativistic wavefunction and properties can be determined within the 1-component framework underlying most electronic structure packages.

The SF-X2C-transformed perturbation operator for HFC can be expressed as

$$\hat{\mathrm{H}}^{(1)}_{\mathrm{N,SF\text{-}X2C}} = \hat{\mathrm{H}}^{\mathrm{FC+SD}}_{\mathrm{N,SF\text{-}X2C}} + \hat{\mathrm{H}}^{\mathrm{PSO}}_{\mathrm{N,SF\text{-}X2C}} \,, \tag{1}$$

$$\begin{aligned}\hat{\mathrm{H}}^{\mathrm{FC+SD}}_{\mathrm{N,SF\text{-}X2C}} = \mu_{\mathrm{B}} g_{\mathrm{N}} \mu_{\mathrm{N}} \frac{\mu_0}{4\pi} \boldsymbol{\sigma} \cdot \Bigg\{ &\hat{\mathrm{U}}^{\dagger}_{\mathrm{UU}} \left(-\frac{\mathbf{r}_{\mathrm{N}}}{r^3_{\mathrm{N}}} \cdot \boldsymbol{\nabla} + \frac{\mathbf{r}_{\mathrm{N}}}{r^3_{\mathrm{N}}} \boldsymbol{\nabla}^{\top} \right) \hat{\mathrm{U}}_{\mathrm{LU}} \\ &+ \hat{\mathrm{U}}^{\dagger}_{\mathrm{LU}} \left(-\frac{\mathbf{r}_{\mathrm{N}}}{r^3_{\mathrm{N}}} \cdot \boldsymbol{\nabla} + \frac{\mathbf{r}_{\mathrm{N}}}{r^3_{\mathrm{N}}} \boldsymbol{\nabla}^{\top} \right)^{\dagger} \hat{\mathrm{U}}_{\mathrm{UU}} \Bigg\} \cdot \mathbf{I}_{\mathrm{N}} \,, \end{aligned} \tag{2}$$

$$\hat{\mathrm{H}}^{\mathrm{PSO}}_{\mathrm{N,SF\text{-}X2C}} = i\, \mu_{\mathrm{B}} g_{\mathrm{N}} \mu_{\mathrm{N}} \frac{\mu_0}{4\pi} \left\{ -\hat{\mathrm{U}}^{\dagger}_{\mathrm{UU}} \left(\frac{\mathbf{r}_{\mathrm{N}}}{r^3_{\mathrm{N}}} \times \boldsymbol{\nabla} \right) \hat{\mathrm{U}}_{\mathrm{LU}} + \hat{\mathrm{U}}^{\dagger}_{\mathrm{LU}} \left(\frac{\mathbf{r}_{\mathrm{N}}}{r^3_{\mathrm{N}}} \times \boldsymbol{\nabla} \right)^{\dagger} \hat{\mathrm{U}}_{\mathrm{UU}} \right\} \cdot \mathbf{I}_{\mathrm{N}} \,, \tag{3}$$

where $\hat{\mathrm{U}}_{\mathrm{UU}}$ and $\hat{\mathrm{U}}_{\mathrm{LU}}$ represent blocks of the SR-X2C decoupling matrix, $\boldsymbol{\sigma}$ is the 3-vector of Pauli spin matrices, $\mathbf{I}_{\mathrm{N}}$ is the nuclear spin vector and $\mathbf{r}_{\mathrm{N}}$ denotes the electron position vector with respect to nucleus N. μ_{B}, g_{N}, μ_{N} and μ_0 have their usual meanings of Bohr magneton, nuclear g-factor, nuclear magneton and vacuum permeability, respectively, and i denotes the imaginary unit. In (1), we distinguish between a spin-dependent contribution, $\hat{\mathrm{H}}^{\mathrm{FC+SD}}_{\mathrm{N,SF\text{-}X2C}}$, and an imaginary, spin-independent contribution, $\hat{\mathrm{H}}^{\mathrm{PSO}}_{\mathrm{N,SF\text{-}X2C}}$. In a non-relativistic picture, the first term correpsonds to the sum of the Fermi-coupling (FC) and the spin-dipolar (SD) operators and the paramagnetic spin-orbit (PSO) term models the interaction between the electronic orbital angular momentum and the nuclear spin. The Zeeman perturbation operator can be similarly divided into a spin-Zeeman term and an orbital-Zeeman term.

In the limit of quenched orbital angular momentum (spin-only limit), the PSO term is zero, and elements of the HFC tensor $\boldsymbol{a}_{\mathrm{N}}$ can be evaluated with respect to a spin-free (SF) state Ψ^{SS} as

$$a_{\mathrm{N},kl} = \frac{1}{S} \left\langle \Psi^{SS} \middle| \left(\frac{\partial^2\, \hat{\mathrm{H}}^{\mathrm{FC+SD}}_{\mathrm{N,SF\text{-}X2C}}}{\partial \mathbf{I}_{\mathrm{N}}\, \partial \hat{\mathbf{S}}} \right)_{kl} \middle| \Psi^{SS} \right\rangle , \quad k,l = x,y,z \,. \tag{4}$$

This is equivalent to mapping the *ab initio* HFC operator onto an effective spin Hamiltonian, equation (5), whose model space is the $2S+1$ spin multiplet represented by Ψ^{SS}.

$$\hat{\mathrm{H}}_{\mathrm{spin}} = \mathbf{I}_{\mathrm{N}} \cdot \boldsymbol{a}_{\mathrm{N}} \cdot \mathbf{S} \,, \tag{5}$$

For systems where S is not a good quantum number, we use the pseudospin ($\mathcal{S}$) parame-

terisation, where the model space (pseudospin multiplet) encompasses the lowest-energy $2\mathcal{S}+1$ states in the SO-coupled spectrum. We note that the $\mathcal{S}$ quantum number could represent a true spin S subject to zero-field splitting, a total spin-orbit coupled angular momentum J subject to a crystal field splitting, or the ground Kramers doublet of a crystal-field-split J multiplet, for example. Replacing S with $\mathcal{S}$ in (5) yields the pseudospin Hamiltonian, from which the elements of symmetrised HFC tensor $\boldsymbol{a}_\mathrm{N}\boldsymbol{a}_\mathrm{N}^\top$ are computed via the method of Chibotaru:[16]

$$\left(\boldsymbol{a}_\mathrm{N}\boldsymbol{a}_\mathrm{N}^\top\right)_{kl} = \frac{3}{\mathcal{S}(\mathcal{S}+1)(2\mathcal{S}+1)} \sum_{\mu,\nu}^{2\mathcal{S}+1} \left\langle \Psi_\mu \middle| \frac{\partial\,\hat{\mathrm{H}}^{(1)}_{\mathrm{N,SF\text{-}X2C}}}{\partial I_{\mathrm{N},k}} \middle| \Psi_\nu \right\rangle \left\langle \Psi_\nu \middle| \frac{\partial\,\hat{\mathrm{H}}^{(1)}_{\mathrm{N,SF\text{-}X2C}}}{\partial I_{\mathrm{N},l}} \middle| \Psi_\mu \right\rangle, \quad (6)$$

where Ψ_μ and Ψ_ν are eigenstates in the pseudospin manifold. A similar expression for the symmetrised g-tensor can be obtained by replacing the HFC operator derivative in (6) with the derivative of the Zeeman perturbation operator with respect to a component of the external magnetic field. The eigenvectors and eigenvalues of the symmetrised tensors correspond to the principal axes and the squared principal values of the original tensors; as a result, the pseudospin parameterisation yields unsigned g-values and HFCCs.

S2 Signs of pseudospin HFCCs

Although sign information is lost when modelling HFC using a pseudospin Hamiltonian, the signs of HFCCs influence HYSCORE simulations. We therefore developed a methodology for approximating HFCC signs in order to obtain simulated HYSCORE spectra that can be compared to experimental measurements. This method is implemented as an extension to the Hyperion package.

As shown in equation (1), the HFC operator can be split into a spin-dependent FC+SD contribution and a spin-independent PSO contribution; partial HFCCs can be obtained by replacing the full HFC operator in equation (6) with either the FC+SD part or the PSO part. Note that, since this method ignores any cross terms, the partial HFCCs do not add up to the total HFCCs; nevertheless, in practice, the cross terms are relatively small.

It is therefore possible to deduce the relative signs of the partial and the total HFCCs by comparing magnitudes. This is the first approximation in our methodology, as the three sets of HFCCs are defined with respect to different sets of main axes. To minimise the error

introduced in this step, HFCCs to be compared are grouped in a way that maximises the overlap between their corresponding eigenvectors.

In the following step, we take advantage of the *a posteriori* inclusion of SOC in our chosen electronic structure approach, drawing parallels between spin-only (signed) HFCCs derived from CASSCF/RASSCF and pseudospin (unsigned) HFCCs derived from CASSCF-SO/RASSCF-SO. This is done by rotating the FC+SD spin-only tensor, $\boldsymbol{a}^{\text{FC+SD}}_{\text{spin-only};(\mathbf{x},\mathbf{y},\mathbf{z})}$, from the molecular frame $(\mathbf{x},\mathbf{y},\mathbf{z})$ into the eigenframe $(\mathbf{e}_{\mathcal{S},1},\mathbf{e}_{\mathcal{S},2},\mathbf{e}_{\mathcal{S},3})$ of the FC+SD pseudospin tensor:

$$\boldsymbol{a}^{\text{FC+SD}}_{\text{spin-only};\mathcal{S}} = \begin{pmatrix}\mathbf{e}_{\mathcal{S},1} & \mathbf{e}_{\mathcal{S},2} & \mathbf{e}_{\mathcal{S},3}\end{pmatrix} \boldsymbol{a}^{\text{FC+SD}}_{\text{spin-only};(\mathbf{x},\mathbf{y},\mathbf{z})} \begin{pmatrix}\mathbf{e}_{\mathcal{S},1} \\ \mathbf{e}_{\mathcal{S},2} \\ \mathbf{e}_{\mathcal{S},3}.\end{pmatrix} \tag{7}$$

The signs of the diagonal elements of $\boldsymbol{a}^{\text{FC+SD}}_{\text{spin-only};\mathcal{S}}$ are then assigned to the FC+SD pseudospin HFCCs - in the limit of equivalence between the spin-only and pseudospin parametrisations, this becomes exact. Signs for the total pseudospin HFCCs are obtained by combining the relative sign information inferred in the first step with the absolute signs of the FC+SD eigenvalues deduced in the second step.

The second approximation underlying our methodology is therefore introduced by the forced equivalence between a spin-only multiplet and the set of SO-coupled states forming the pseudospin multiplet. This is, of course, inexact since a SO state is a superposition of multiple spin-free states. Moreover, results depend on which spin-free state is chosen for the comparison. Fortunately, we find this strategy to be straightforward when applied to the [$\text{AnCp}^{\text{tt}}_3$] complexes investigated in this work. [$\text{ThCp}^{\text{tt}}_3$] is, to a good approximation, a 6d^1 doublet and as such, there is a natural correspondence between the lowest energy spin-free state and the lowest-energy SO state. In the case of [UCp^{tt}_3], the ground Kramers doublet has a dominant spin quartet character (see Section S25) and we find that applying our strategy using any of the 13 spin-free quartet states considered herein yields consistent results for all ligand HFCCs studied.

S3 Principal directions of the theoretical *g*-matrices

All HYSCORE simulations reported herein use the experimental frozen solution *g*-values. The orientation of the calculated *g*-matrix principal axes with respect to the molecular XRD structure of [$\text{ThCp}^{\text{tt}}_3$] and [$\text{UCp}^{\text{tt}}_3$] is shown in Figure S1 and Figure S2, respectively. We

use the principal axes of the g and HFC matrices determined from *ab initio* data via Hyperion and as such, each electronic structure model (choice of CAS/RAS and number of optimised states) yields a different set of directions. Note that the state-specific (SS) calculations on $[\mathrm{ThCp}^{\mathrm{tt}}_3]$ yield three isotropic g-values ($g = 2$) and hence the resulting principal axes follow the molecular reference frame directions. In this case, a straightforward correspondence between theoretical and experimental g-values does not exist. Nevertheless, for the HYSCORE simulations presented in this work, we assign the experimental $g_{\|}$ value to the direction that lies closest to the pseudo-C_3 axis, which is consistent with the results of state-averaged calculations.

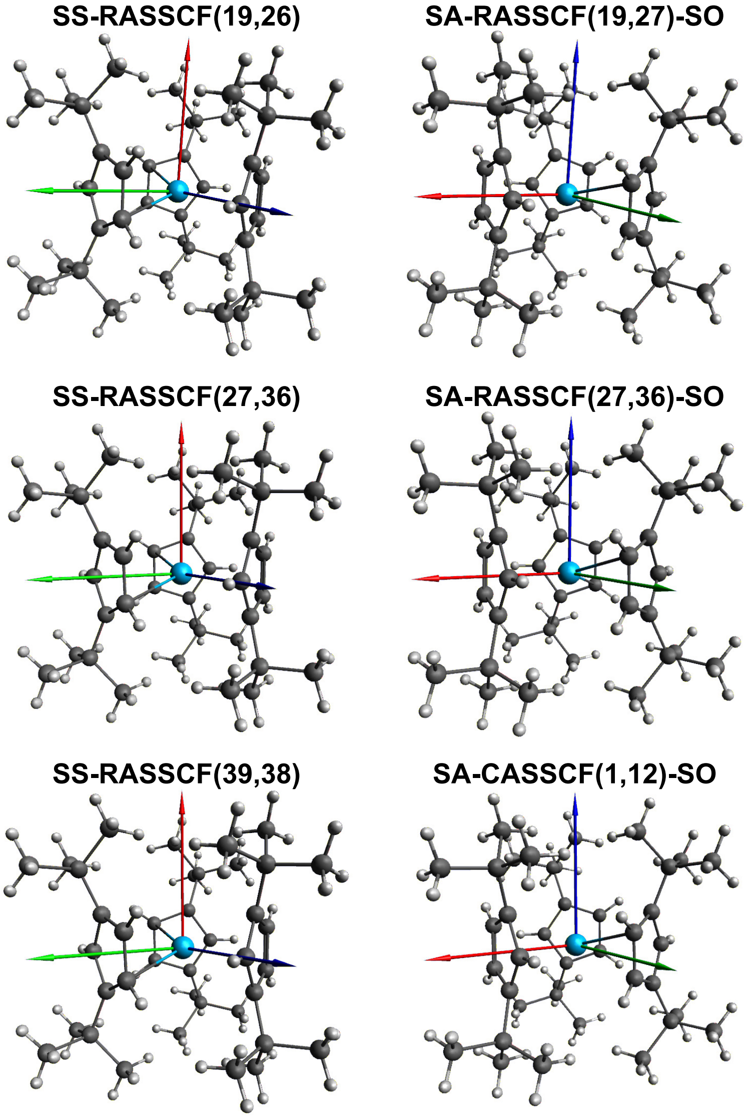

Figure S1: Principal axes for the g-matrix of $[\mathrm{ThCp}^{\mathrm{tt}}_3]$, as calculated via Hyperion from electronic structure data using the XRD structure. Axes corresponding to g_x and g_y (i.e. $g_\perp$) are shown in red and green, respectively. The blue axis corresponds to g_z ($g_{\|}$).

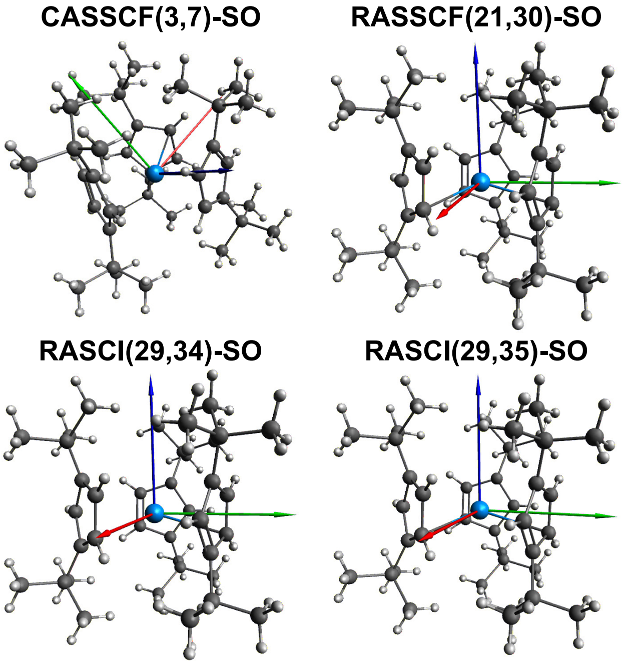

Figure S2: Principal axes for the g-matrix of [UCp$_3^{\mathrm{tt}}$], as calculated via Hyperion from electronic structure data using the XRD structure. Axes corresponding to g_x, g_y and g_z are shown in red, green and blue, respectively.

S4 Active Space Orbitals

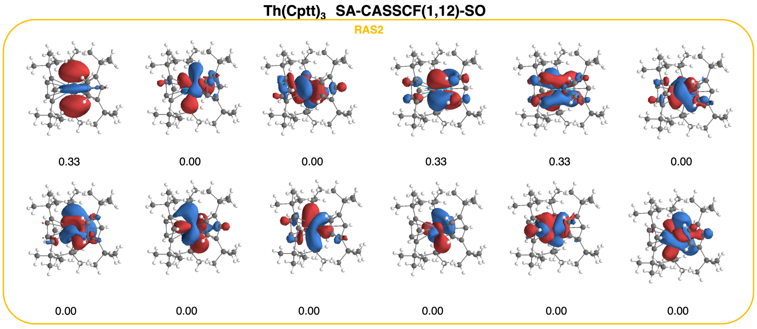

Figure S3: The SA-CASSCF(1,12)-SO active space molecular orbitals for [$ThCp^{tt}_3$] (XRD structure).

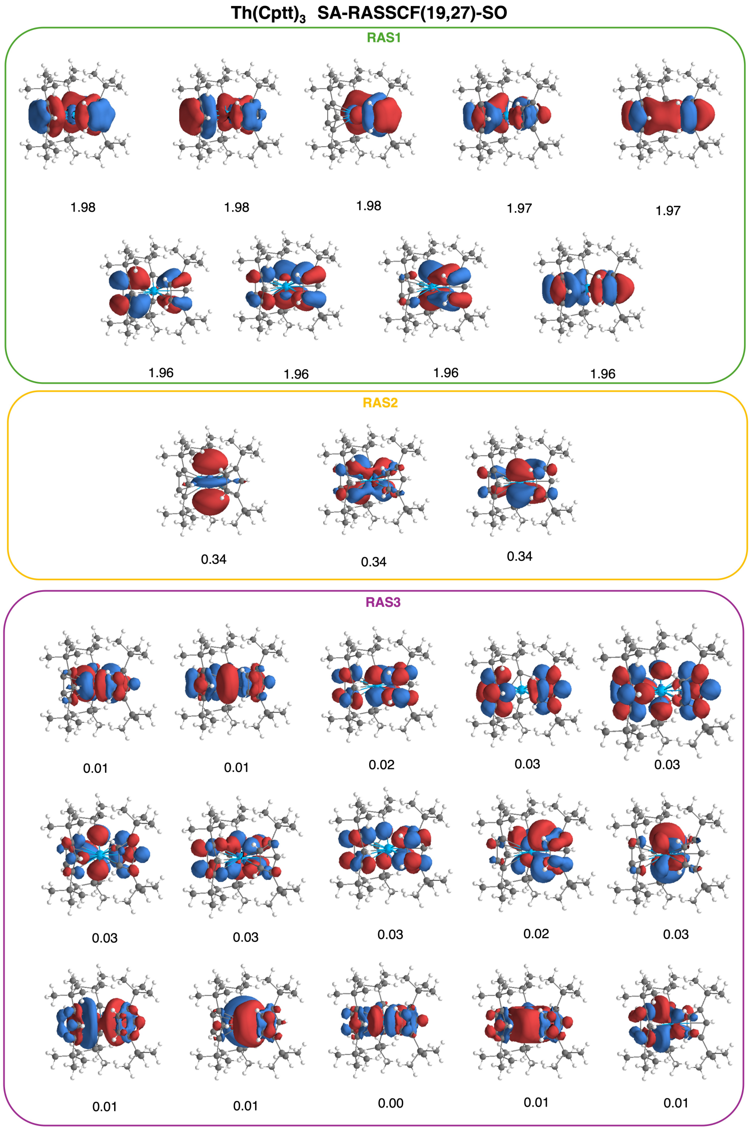

Figure S4: The SA-RASSCF(19,27)-SO active space molecular orbitals for [ThCp$_3^{tt}$] (XRD structure).

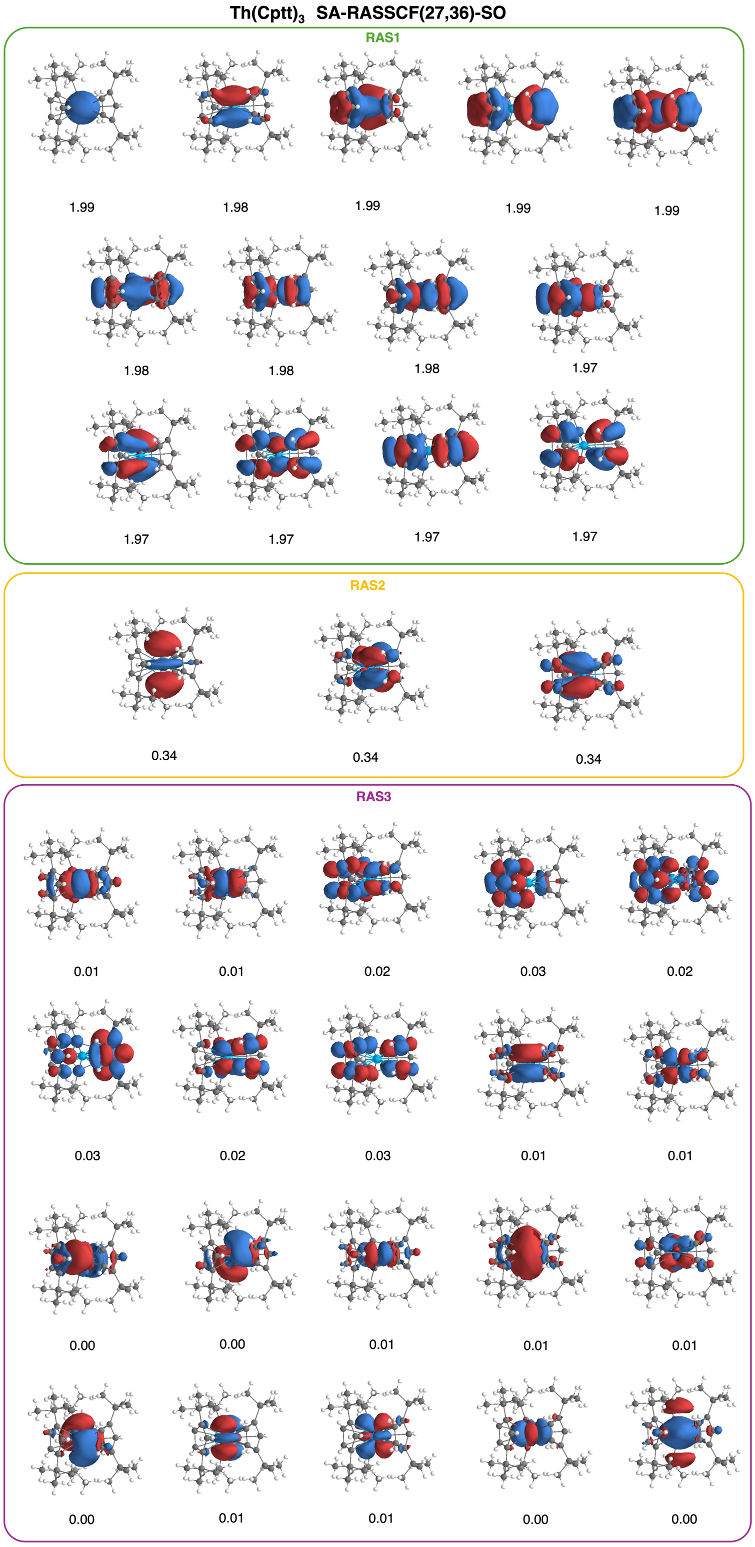

Figure S5: The SA-RASSCF(27,36)-SO active space molecular orbitals for [$ThCp^{tt}_3$] (XRD structure).

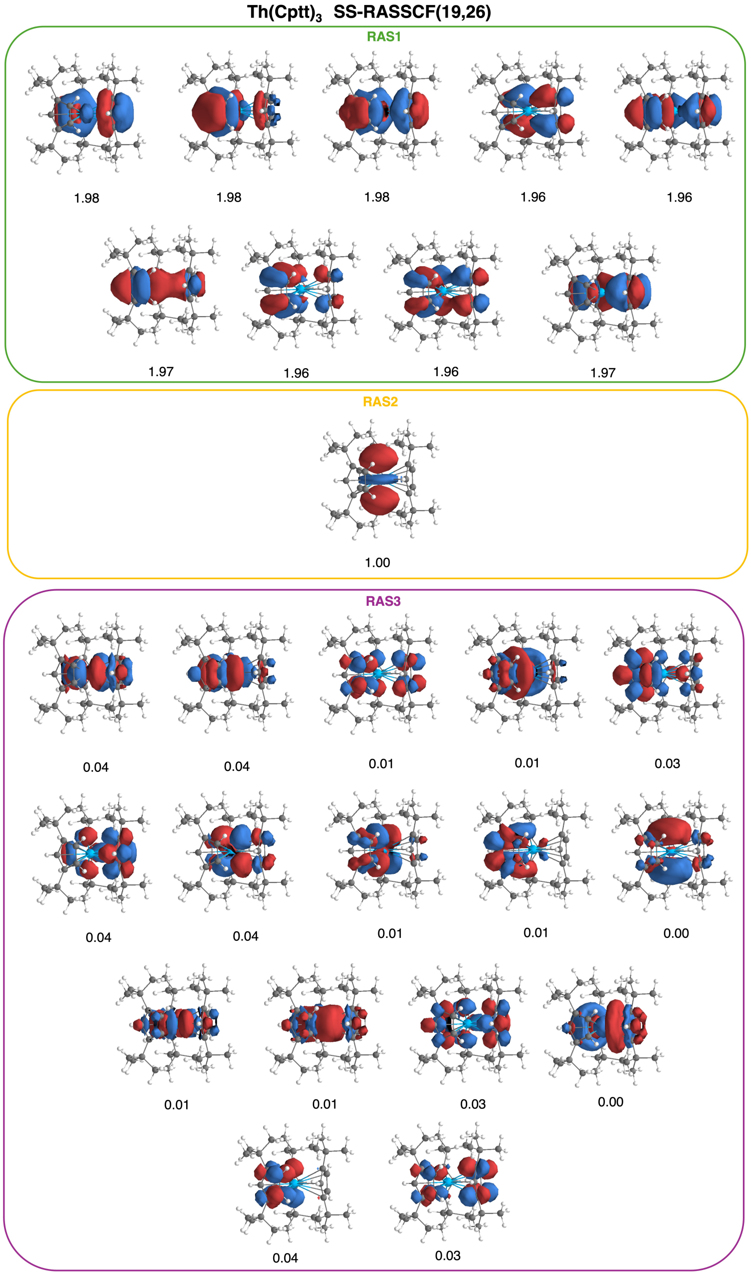

Figure S6: The SS-RASSCF(19,26) active space molecular orbitals for [ThCp$^{tt}_3$] (XRD structure).

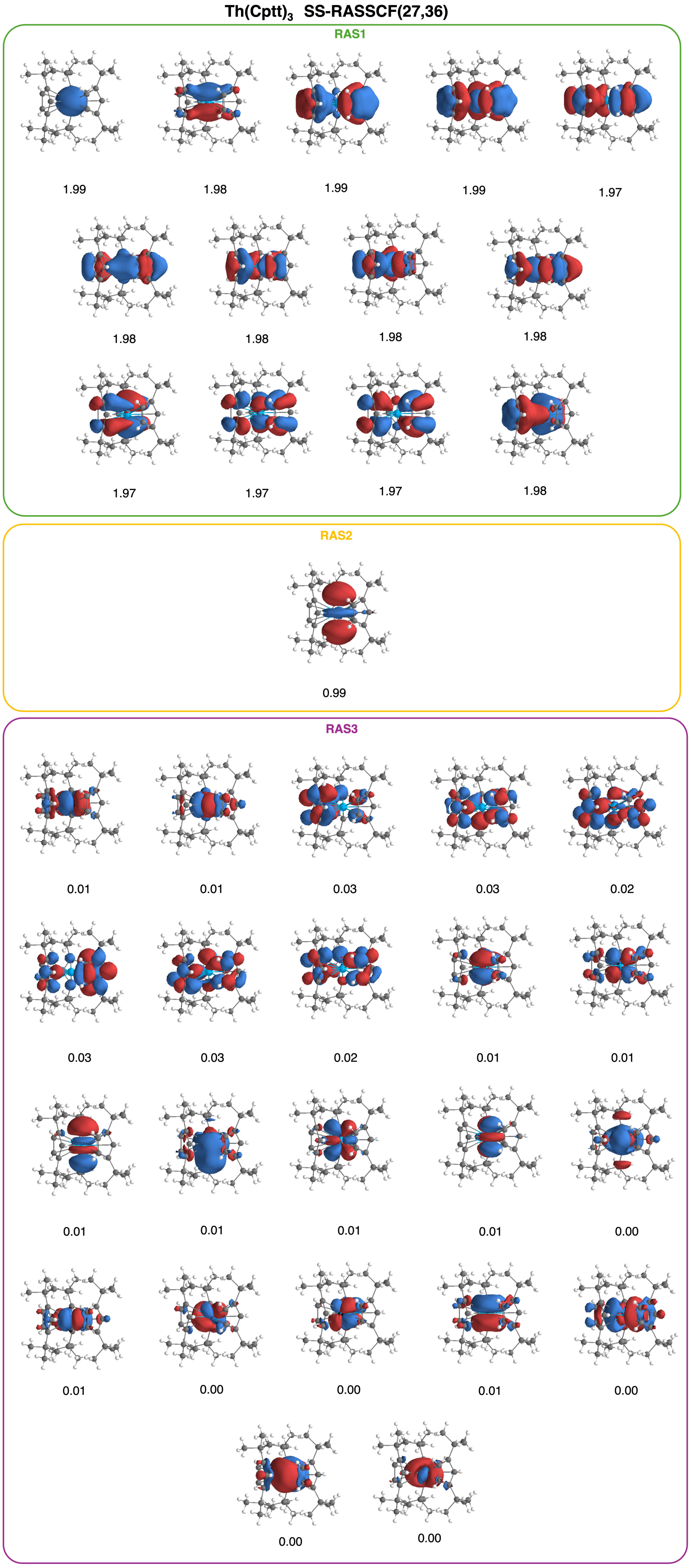

Figure S7: The SS-RASSCF(27,36) active space molecular orbitals for [ThCp$^{tt}_3$] (XRD structure).

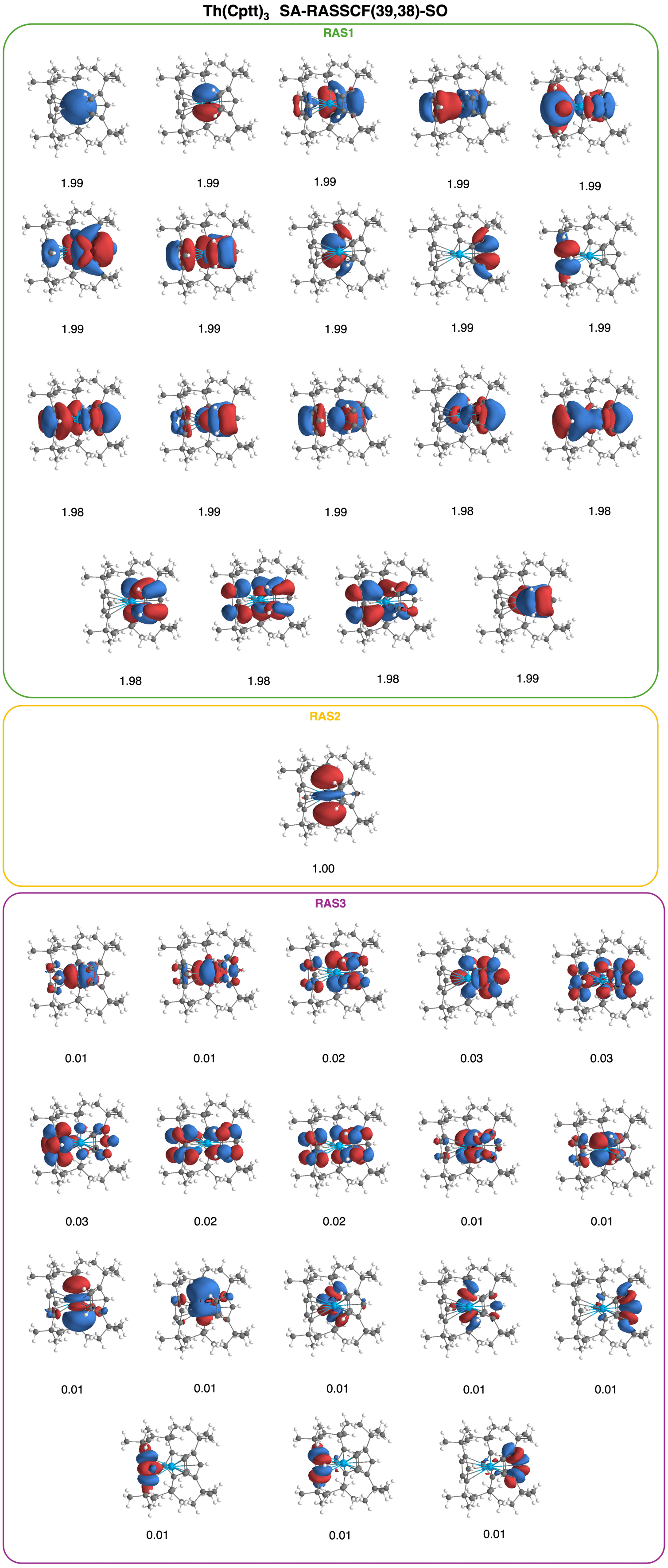

Figure S8: The SS-RASSCF(39,38) active space molecular orbitals for [ThCp$^{tt}_3$] (XRD structure).

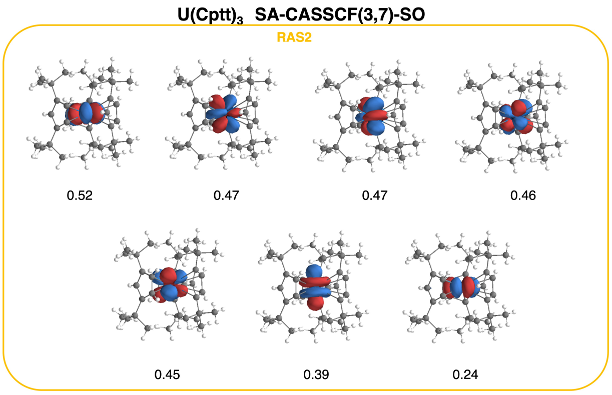

Figure S9: The SA-CASSCF(3,7)-SO active space molecular orbitals for [UCp^{tt}_3] (XRD structure).

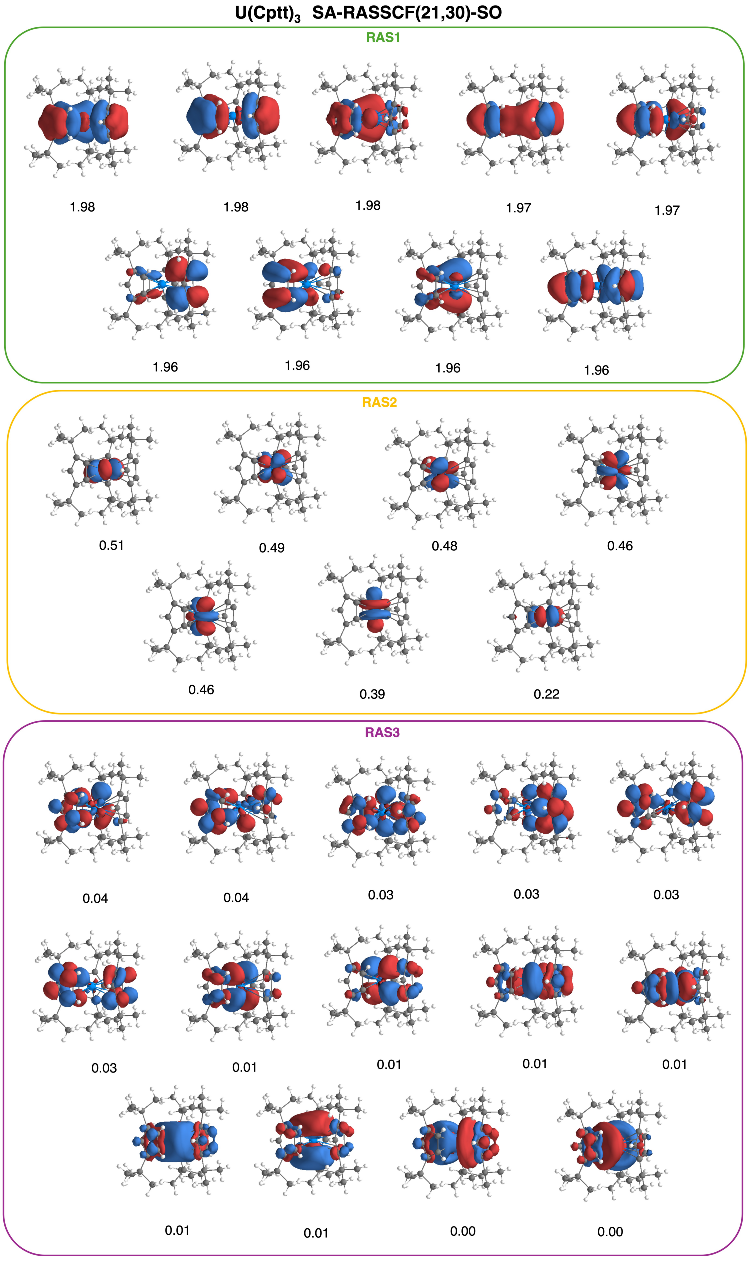

Figure S10: The SA-RASSCF(21,30)-SO active space molecular orbitals for [UCp^{tt}_3] (XRD structure).

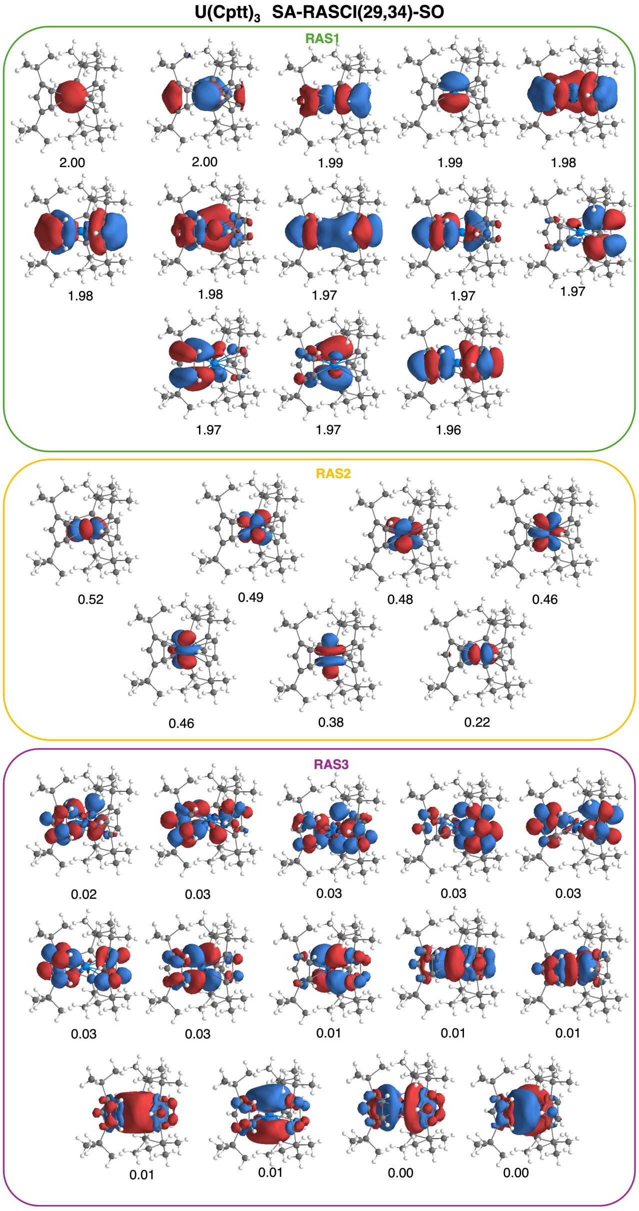

Figure S11: The SA-RASCI(29,34)-SO active space molecular orbitals for $[UCp^{tt}_3]$ (XRD structure).

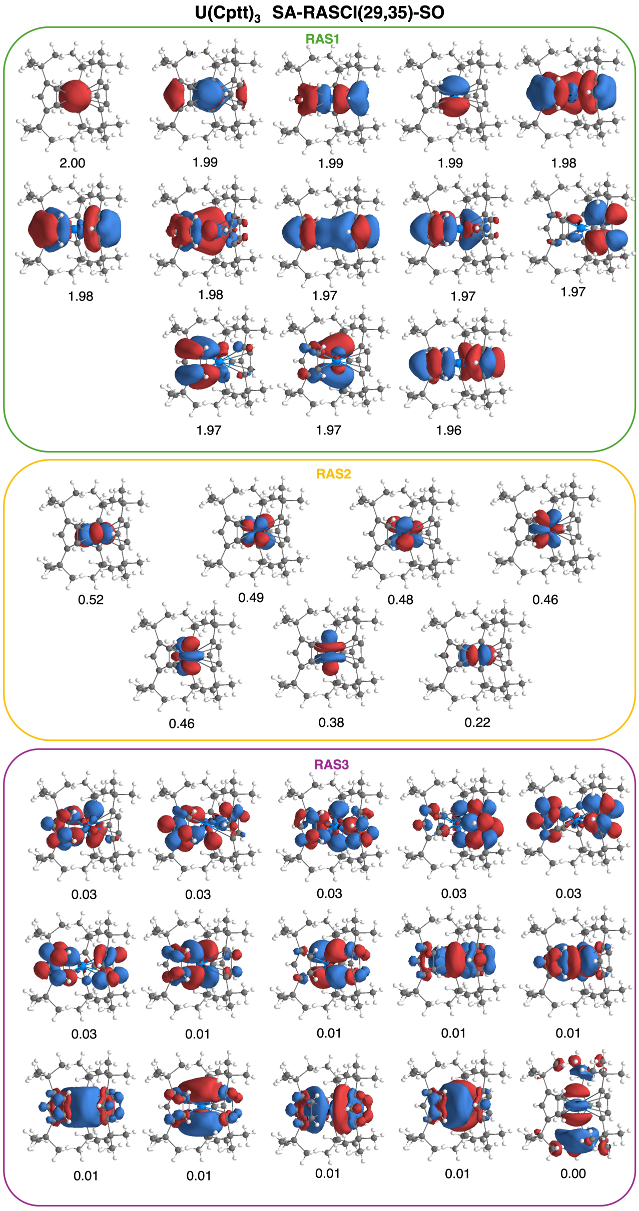

Figure S12: The SA-RASCI(29,35)-SO active space molecular orbitals for $[UCp^{tt}_3]$ (XRD structure).

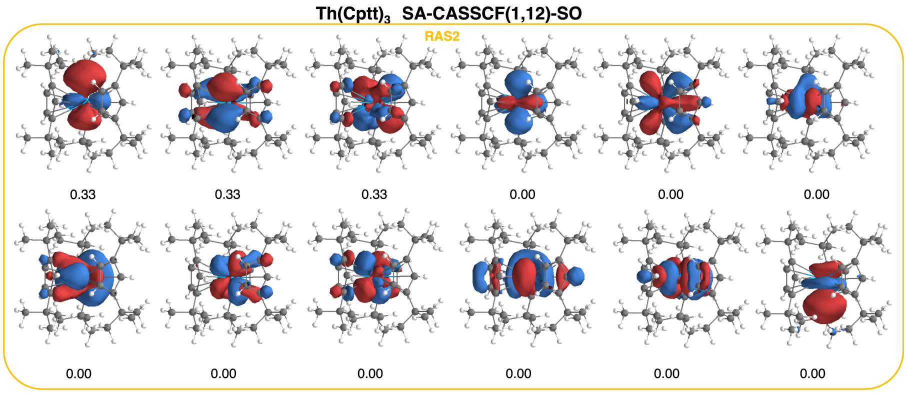

Figure S13: The SA-CASSCF(1,12)-SO active space molecular orbitals for $[ThCp^{tt}_3]$ (Optimised structure).

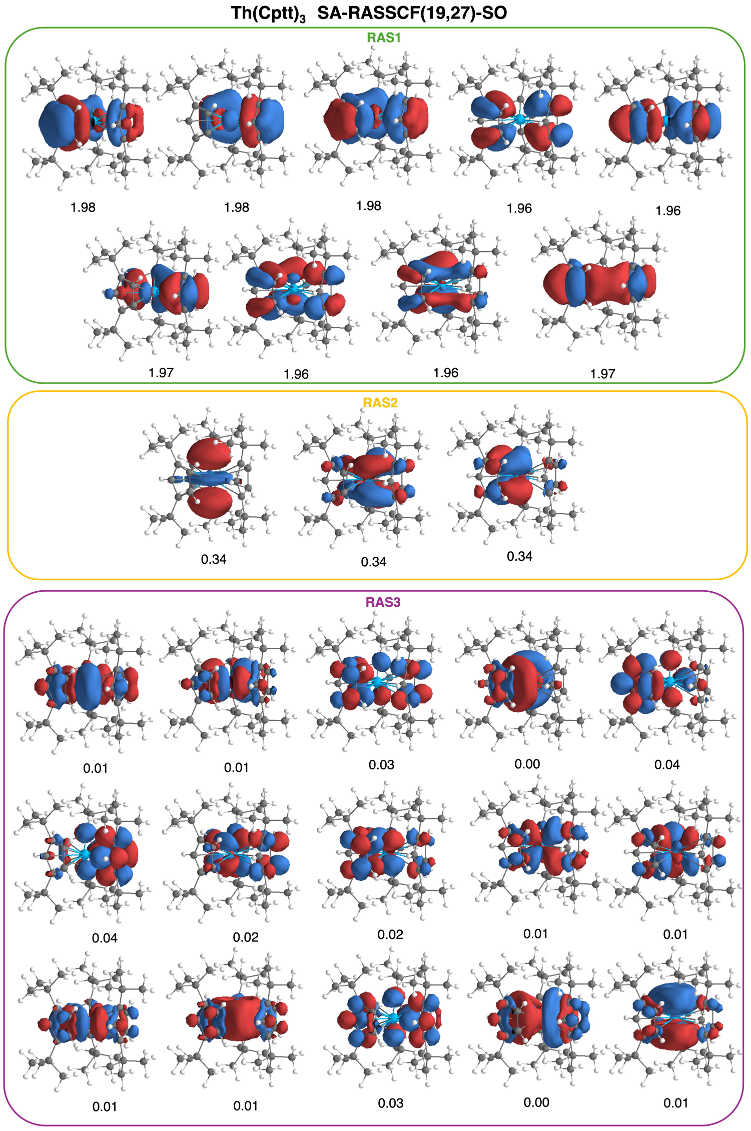

Figure S14: The SA-RASSCF(19,27)-SO active space molecular orbitals for [ThCp$^{tt}_3$] (Optimised structure).

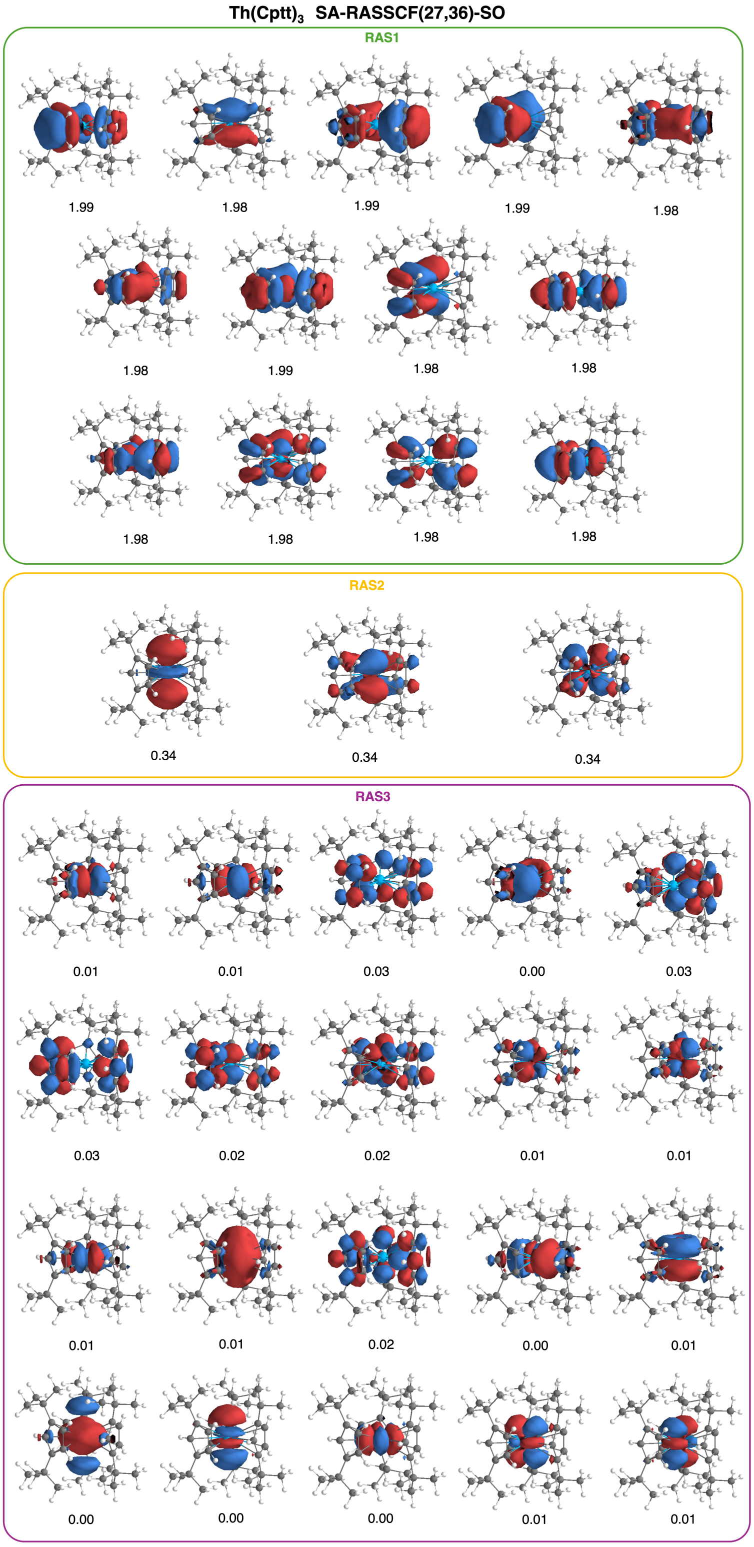

Figure S15: The SA-RASSCF(27,36)-SO active space molecular orbitals for [$ThCp^{tt}_3$] (Optimised structure).

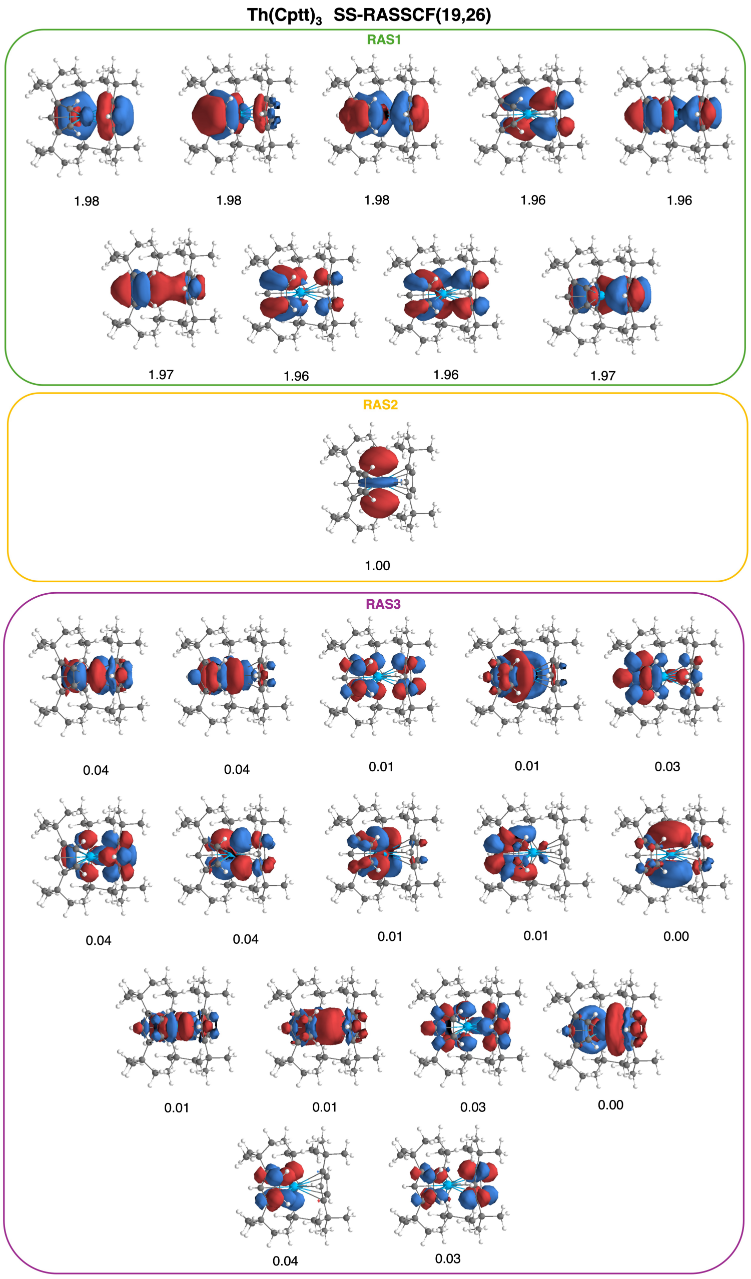

Figure S16: The SS-RASSCF(19,26) active space molecular orbitals for [$ThCp^{tt}_3$] (Optimised structure).

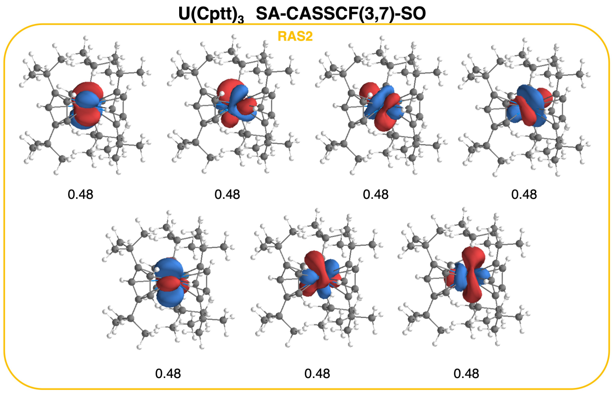

Figure S17: The SA-CASSCF(3,7)-SO active space molecular orbitals for [UCp^{tt}_3] (Optimised structure).

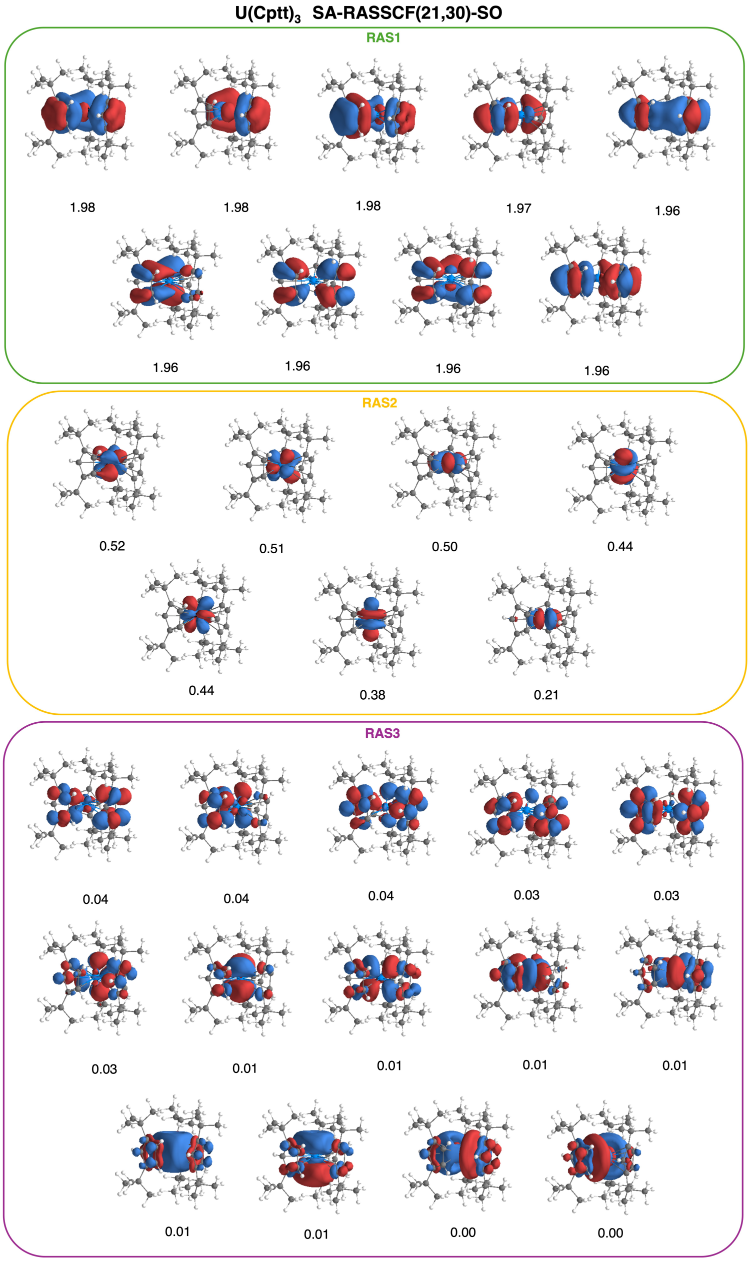

Figure S18: The SA-RASSCF(21,30)-SO active space molecular orbitals for [UCp$^{tt}_3$] (Optimised structure).

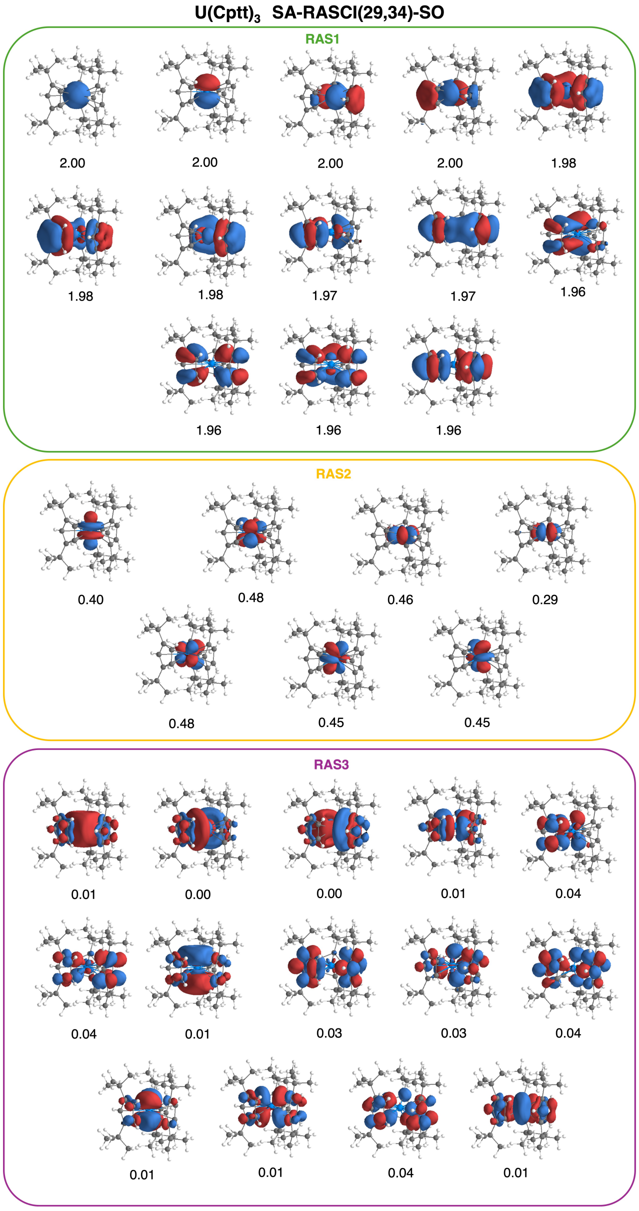

Figure S19: The SA-RASCI(29,34)-SO active space molecular orbitals for [UCp^{tt}_3] (Optimised structure).

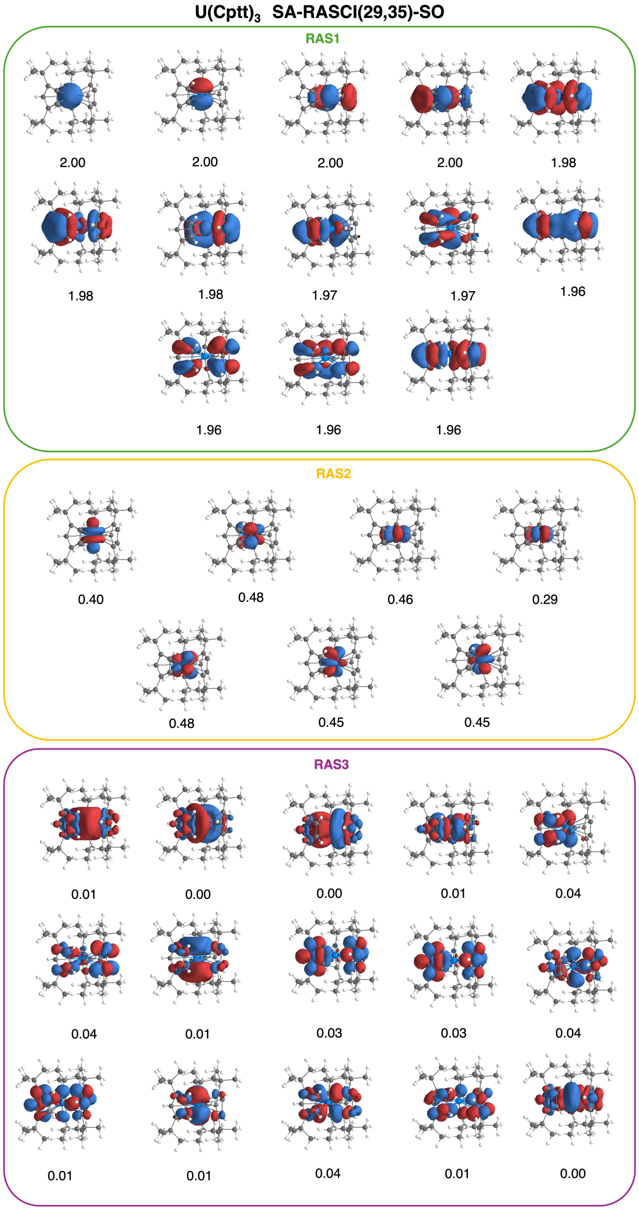

Figure S20: The SA-RASCI(29,35)-SO active space molecular orbitals for [UCp$_3^{\mathrm{tt}}$] (Optimised structure).

S5 Spin Density of [AnCp$_3^{tt}$]

(a) [ThCp$_3^{tt}$] **(b)** [UCp$_3^{tt}$]

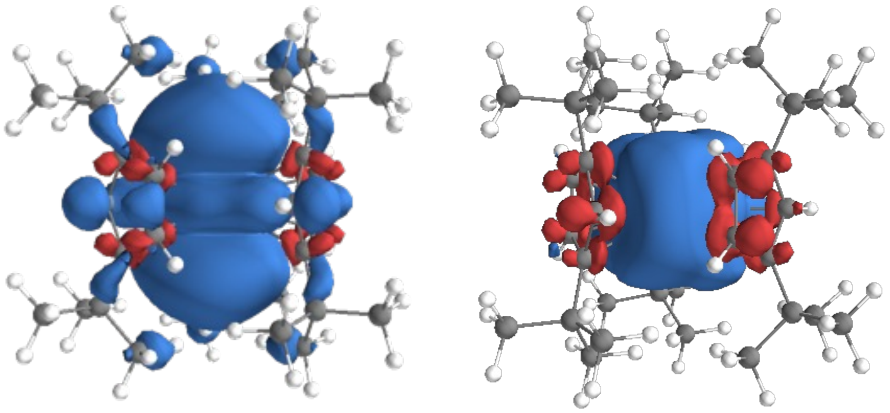

Figure S21: The spin density of a) the ground spin-free state of [ThCp$_3^{tt}$] and b) the fourth spin-free state of [UCp$_3^{tt}$], from calculations using the XRD structure, which provide the largest contribution to the ground spin-orbit state.

S6 Hybrid 6d/5f Orbitals for [ThCp$^{tt}_3$]

(a) Root 2

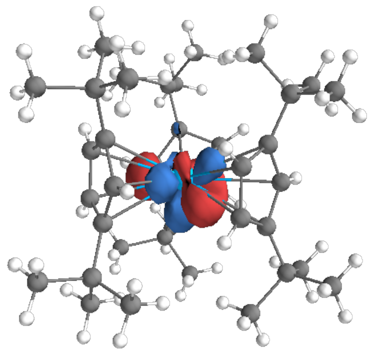

(b) Root 3

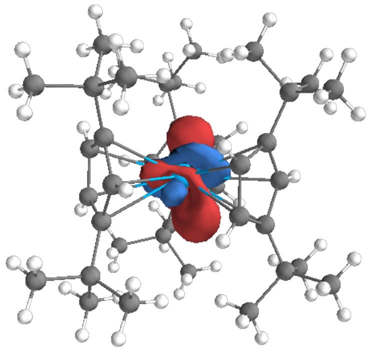

Figure S22: The pair of hybridised natural singly occupied molecular orbitals for the first and second pseudo-degenerate excited states of [ThCp$^{tt}_3$] (XRD structure).

S7 Computed Hyperfine Coupling Tensors

Table S1: SS-RASSCF(39,38) calculated hyperfine coupling tensors (in MHz) for the ^{1}H and ^{13}C nuclei in [$ThCp^{tt}_3$] (XRD structure).

Atom	A_1	A_2	A_3	A_{iso}	A_{Euclid}
C1	-0.786	-0.583	0.622	-0.249	1.159
C2	-0.153	-0.035	3.513	1.108	3.516
C3	-0.75	-0.587	0.567	-0.257	1.109
C4	-0.734	-0.017	1.024	0.091	1.26
C5	-0.72	-0.016	1.035	0.100	1.261
C6	1.294	1.433	2.457	1.728	3.124
C7	-0.142	-0.107	0.341	0.031	0.385
C8	0.514	0.58	1.392	0.829	1.593
C9	0.5	0.698	1.59	0.929	1.807
C10	1.087	1.211	2.194	1.497	2.731
C11	0.837	1.006	1.946	1.263	2.345
C12	-0.111	-0.078	0.359	0.057	0.384
C13	0.068	0.099	0.721	0.296	0.731
C14	-0.834	-0.674	0.469	-0.346	1.17
C15	-0.133	-0.024	3.668	1.170	3.67
C16	-0.826	-0.645	0.573	-0.299	1.194
C17	-0.759	-0.095	1.132	0.093	1.366
C18	-0.688	0.06	0.884	0.085	1.122
C19	0.906	1.039	1.986	1.310	2.417
C20	1.694	1.879	3.227	2.267	4.1
C21	0.04	0.068	0.678	0.262	0.683
C22	-0.131	-0.1	0.329	0.033	0.368
C23	1.186	1.34	2.372	1.633	2.971
C24	-0.101	-0.073	0.393	0.073	0.412
C25	-0.051	-0.029	0.546	0.155	0.549
C26	2.36	2.544	3.922	2.942	5.237
C27	-0.78	-0.584	0.693	-0.224	1.196
C28	-0.134	-0.038	3.399	1.076	3.402
C29	-0.694	-0.508	0.526	-0.225	1.009
C30	-0.711	0.041	0.966	0.099	1.2
C31	-0.75	-0.067	1.073	0.085	1.311
C32	1.191	1.333	2.346	1.623	2.949
C33	1.222	1.419	2.546	1.729	3.161
C34	0.224	0.264	0.969	0.486	1.029
C35	-0.121	-0.089	0.366	0.052	0.396
C36	0.968	1.087	2.044	1.366	2.51
C37	-0.132	-0.1	0.321	0.030	0.362
C38	0.088	0.122	0.763	0.324	0.777
C39	0.834	1.012	1.969	1.272	2.366
H1	-1.385	-1.248	2.782	0.050	3.349
H2	-1.204	-0.572	3.524	0.583	3.767
H3	-1.200	-0.579	3.625	0.615	3.862
H4	-0.214	-0.134	0.914	0.189	0.948
H5	-0.479	-0.398	0.936	0.020	1.124
H6	-0.514	-0.386	0.908	0.003	1.112

Table S1: SS-RASSCF(39,38) calculated hyperfine coupling tensors (in MHz) for the ^{1}H and ^{13}C nuclei in [ThCp$^{tt}_3$] (XRD structure).

Atom	A_1	A_2	A_3	A_{iso}	A_{Euclid}
H7	-0.892	-0.701	1.617	0.008	1.975
H8	-1.344	-1.153	3.538	0.347	3.956
H9	-0.554	-0.499	1.256	0.068	1.460
H10	-0.655	-0.564	1.361	0.047	1.612
H11	-1.410	-1.041	4.528	0.692	4.856
H12	-0.834	-0.766	1.670	0.023	2.017
H13	-0.509	-0.417	1.464	0.179	1.606
H14	-0.975	-0.890	1.872	0.002	2.290
H15	-1.224	-0.837	4.706	0.882	4.934
H16	-0.486	-0.365	0.861	0.003	1.054
H17	-0.476	-0.404	0.936	0.019	1.125
H18	-0.200	-0.124	0.883	0.186	0.914
H19	-0.517	-0.478	1.006	0.004	1.228
H20	-1.159	-1.058	2.437	0.073	2.898
H21	-0.730	-0.581	1.324	0.004	1.620
H22	-1.414	-1.218	2.790	0.053	3.356
H23	-1.304	-0.658	3.494	0.511	3.787
H24	-1.125	-0.544	3.626	0.652	3.836
H25	-0.577	-0.447	1.729	0.235	1.877
H26	-1.373	-0.823	6.173	1.326	6.378
H27	-1.032	-0.917	1.950	0.000	2.389
H28	-0.685	-0.542	1.248	0.007	1.523
H29	-1.177	-1.055	2.383	0.050	2.860
H30	-0.532	-0.491	1.023	0.000	1.254
H31	-0.188	-0.113	0.920	0.206	0.946
H32	-0.486	-0.413	0.963	0.021	1.155
H33	-0.475	-0.358	0.866	0.011	1.051
H34	-0.472	-0.357	0.871	0.014	1.053
H35	-0.507	-0.438	1.020	0.025	1.221
H36	-0.143	-0.063	1.002	0.265	1.014
H37	-0.504	-0.467	0.977	0.002	1.194
H38	-1.093	-0.974	2.132	0.022	2.587
H39	-0.674	-0.534	1.219	0.004	1.492
H40	-1.166	-1.000	2.308	0.047	2.772
H41	-1.140	-0.505	6.431	1.595	6.551
H42	-0.339	-0.211	1.999	0.483	2.039
H43	-1.356	-1.212	2.708	0.047	3.262
H44	-1.124	-0.528	3.626	0.658	3.833
H45	-1.286	-0.648	3.560	0.542	3.840
H46	-0.602	-0.490	1.580	0.163	1.760
H47	-1.348	-0.859	5.449	1.081	5.678
H48	-0.986	-0.884	1.876	0.002	2.296
H49	-0.564	-0.524	1.122	0.011	1.361
H50	-0.762	-0.607	1.409	0.013	1.712
H51	-1.277	-1.169	2.921	0.158	3.396
H52	-0.483	-0.406	0.960	0.024	1.149
H53	-0.493	-0.372	0.883	0.006	1.077
H54	-0.182	-0.101	0.947	0.221	0.970

Table S1: SS-RASSCF(39,38) calculated hyperfine coupling tensors (in MHz) for the ^{1}H and ^{13}C nuclei in [ThCp$^{tt}_{3}$] (XRD structure).

Atom	A_1	A_2	A_3	A_{iso}	A_{Euclid}
H55	-0.486	-0.366	0.863	0.004	1.056
H56	-0.471	-0.401	0.920	0.016	1.109
H57	-0.219	-0.148	0.860	0.164	0.900
H58	-1.190	-1.086	2.559	0.094	3.024
H59	-0.739	-0.584	1.343	0.007	1.641
H60	-0.524	-0.482	1.032	0.009	1.254
H61	-0.502	-0.408	1.500	0.197	1.633
H62	-1.000	-0.906	1.918	0.004	2.345
H63	-1.221	-0.832	4.853	0.933	5.073

Table S2: SS-RASSCF(27,36) calculated hyperfine coupling tensors (in MHz) for the ^{1}H and ^{13}C nuclei in [ThCp$^{tt}_{3}$] (XRD structure).

Atom	A_1	A_2	A_3	A_{iso}	A_{Euclid}
C1	-0.933	-0.642	0.673	-0.301	1.318
C2	-1.205	-1.069	2.361	0.029	2.858
C3	-0.897	-0.640	0.597	-0.313	1.253
C4	-0.777	-0.078	0.776	-0.026	1.100
C5	-0.764	-0.072	0.766	-0.023	1.084
C6	1.287	1.427	2.454	1.723	3.117
C7	-0.138	-0.102	0.354	0.038	0.393
C8	0.523	0.587	1.395	0.835	1.601
C9	0.504	0.699	1.580	0.928	1.799
C10	1.101	1.227	2.220	1.516	2.765
C11	0.859	1.025	1.962	1.282	2.375
C12	-0.109	-0.076	0.371	0.062	0.394
C13	0.075	0.106	0.732	0.304	0.743
C14	-0.940	-0.692	0.526	-0.369	1.281
C15	-1.235	-1.110	2.487	0.047	2.990
C16	-0.973	-0.706	0.617	-0.354	1.351
C17	-0.798	-0.149	0.887	-0.020	1.203
C18	-0.729	0.010	0.631	-0.029	0.964
C19	0.918	1.052	2.005	1.325	2.443
C20	1.698	1.879	3.201	2.259	4.082
C21	0.047	0.075	0.685	0.269	0.691
C22	-0.129	-0.098	0.339	0.037	0.376
C23	1.203	1.359	2.401	1.654	3.010
C24	-0.099	-0.071	0.404	0.078	0.422
C25	-0.047	-0.025	0.554	0.161	0.556
C26	2.388	2.567	3.933	2.963	5.269
C27	-0.930	-0.661	0.744	-0.282	1.362
C28	-1.157	-1.044	2.290	0.030	2.770
C29	-0.814	-0.549	0.544	-0.273	1.122
C30	-0.763	-0.029	0.708	-0.028	1.041
C31	-0.787	-0.116	0.808	-0.032	1.133
C32	1.190	1.333	2.350	1.624	2.952
C33	1.223	1.416	2.525	1.721	3.142

Table S2: SS-RASSCF(27,36) calculated hyperfine coupling tensors (in MHz) for the ^{1}H and ^{13}C nuclei in [ThCp$^{tt}_{3}$] (XRD structure).

Atom	A_1	A_2	A_3	A_{iso}	A_{Euclid}
C34	0.231	0.270	0.974	0.492	1.037
C35	-0.116	-0.084	0.381	0.060	0.407
C36	0.980	1.101	2.067	1.383	2.538
C37	-0.132	-0.100	0.330	0.033	0.369
C38	0.095	0.129	0.773	0.332	0.789
C39	0.855	1.030	1.984	1.290	2.393
H1	-1.416	-1.331	2.808	0.020	3.415
H2	-1.269	-0.620	3.495	0.535	3.770
H3	-1.260	-0.623	3.571	0.563	3.838
H4	-0.205	-0.124	0.925	0.199	0.956
H5	-0.479	-0.401	0.939	0.020	1.128
H6	-0.516	-0.388	0.912	0.003	1.118
H7	-0.893	-0.704	1.617	0.007	1.977
H8	-1.352	-1.157	3.482	0.324	3.911
H9	-0.553	-0.498	1.248	0.066	1.454
H10	-0.649	-0.559	1.351	0.048	1.600
H11	-1.420	-1.051	4.439	0.656	4.777
H12	-0.828	-0.768	1.660	0.021	2.007
H13	-0.507	-0.417	1.464	0.180	1.605
H14	-0.971	-0.894	1.874	0.003	2.292
H15	-1.251	-0.863	4.652	0.846	4.895
H16	-0.490	-0.368	0.867	0.003	1.062
H17	-0.477	-0.409	0.940	0.018	1.130
H18	-0.193	-0.116	0.896	0.196	0.924
H19	-0.519	-0.480	1.009	0.003	1.232
H20	-1.166	-1.063	2.433	0.068	2.900
H21	-0.734	-0.588	1.333	0.004	1.632
H22	-1.451	-1.303	2.817	0.021	3.426
H23	-1.367	-0.705	3.475	0.468	3.800
H24	-1.185	-0.584	3.579	0.603	3.815
H25	-0.570	-0.446	1.714	0.233	1.861
H26	-1.405	-0.855	6.031	1.257	6.251
H27	-1.022	-0.915	1.937	0.000	2.374
H28	-0.687	-0.547	1.252	0.006	1.529
H29	-1.177	-1.052	2.369	0.047	2.847
H30	-0.531	-0.491	1.021	0.000	1.251
H31	-0.177	-0.102	0.933	0.218	0.955
H32	-0.485	-0.417	0.964	0.021	1.157
H33	-0.478	-0.360	0.871	0.011	1.057
H34	-0.476	-0.360	0.879	0.014	1.062
H35	-0.508	-0.444	1.025	0.024	1.227
H36	-0.134	-0.053	1.017	0.277	1.028
H37	-0.506	-0.469	0.980	0.002	1.199
H38	-1.098	-0.978	2.136	0.020	2.593
H39	-0.678	-0.540	1.228	0.003	1.504

Table S3: SS-RASSCF(19,26) calculated hyperfine coupling tensors (in MHz) for the ^{1}H and ^{13}C nuclei in [ThCp$^{tt}_3$] (XRD structure).

Atom	A_1	A_2	A_3	A_{iso}	A_{Euclid}
C1	-0.995	-0.800	-0.119	-0.638	1.282
C2	-1.319	-1.212	2.576	0.015	3.137
C3	-1.073	-0.865	-0.166	-0.701	1.388
C4	-0.624	0.107	0.679	0.054	0.928
C5	-0.618	0.101	0.704	0.062	0.942
C6	1.414	1.552	2.591	1.852	3.335
C7	-0.119	-0.084	0.379	0.059	0.406
C8	0.653	0.719	1.551	0.974	1.830
C9	0.626	0.827	1.750	1.068	2.034
C10	1.203	1.327	2.326	1.619	2.935
C11	1.028	1.196	2.166	1.463	2.679
C12	-0.076	-0.044	0.410	0.097	0.419
C13	0.127	0.160	0.791	0.359	0.817
C14	-1.156	-0.933	-0.311	-0.800	1.518
C15	-1.362	-1.266	2.720	0.031	3.295
C16	-1.068	-0.913	-0.199	-0.727	1.419
C17	-0.642	0.004	0.772	0.057	1.005
C18	-0.593	0.158	0.629	0.065	0.879
C19	1.008	1.140	2.100	1.416	2.594
C20	1.994	2.175	3.575	2.581	4.635
C21	0.090	0.120	0.737	0.316	0.752
C22	-0.105	-0.075	0.366	0.062	0.388
C23	1.305	1.462	2.510	1.759	3.184
C24	-0.061	-0.035	0.449	0.118	0.455
C25	-0.020	0.003	0.583	0.189	0.583
C26	2.737	2.914	4.338	3.330	5.899
C27	-0.983	-0.813	-0.113	-0.636	1.280
C28	-1.269	-1.180	2.501	0.017	3.042
C29	-1.029	-0.709	-0.152	-0.630	1.259
C30	-0.607	0.130	0.644	0.056	0.894
C31	-0.630	0.054	0.716	0.047	0.956
C32	1.302	1.444	2.472	1.739	3.146
C33	1.442	1.637	2.805	1.961	3.553
C34	0.311	0.351	1.070	0.577	1.168
C35	-0.088	-0.056	0.416	0.091	0.429
C36	1.066	1.183	2.152	1.467	2.677
C37	-0.105	-0.073	0.360	0.061	0.382
C38	0.151	0.187	0.836	0.391	0.870
C39	1.015	1.192	2.179	1.462	2.683
H1	-1.476	-1.312	2.882	0.0313	3.493
H2	-1.266	-0.509	3.580	0.6017	3.831
H3	-1.254	-0.517	3.689	0.6393	3.931
H4	-0.168	-0.083	0.966	0.2383	0.983
H5	-0.465	-0.390	0.930	0.0250	1.110
H6	-0.514	-0.372	0.905	0.0063	1.105
H7	-0.909	-0.698	1.629	0.0073	1.992
H8	-1.327	-1.117	3.579	0.3783	3.977
H9	-0.548	-0.490	1.276	0.0793	1.473

Table S3: SS-RASSCF(19,26) calculated hyperfine coupling tensors (in MHz) for the ^{1}H and ^{13}C nuclei in [$ThCp^{tt}_3$] (XRD structure).

Atom	A_1	A_2	A_3	A_{iso}	A_{Euclid}
H10	-0.652	-0.562	1.381	0.0557	1.627
H11	-1.373	-0.979	4.604	0.7507	4.903
H12	-0.840	-0.772	1.678	0.0220	2.029
H13	-0.487	-0.396	1.501	0.2060	1.627
H14	-0.978	-0.891	1.885	0.0053	2.303
H15	-1.165	-0.752	4.785	0.9560	4.982
H16	-0.483	-0.351	0.854	0.0067	1.042
H17	-0.460	-0.395	0.930	0.0250	1.110
H18	-0.149	-0.070	0.937	0.2393	0.952
H19	-0.518	-0.478	1.014	0.0060	1.235
H20	-1.156	-1.049	2.463	0.0860	2.916
H21	-0.742	-0.579	1.333	0.0040	1.631
H22	-1.517	-1.276	2.888	0.0317	3.503
H23	-1.364	-0.610	3.558	0.5280	3.859
H24	-1.184	-0.469	3.694	0.6803	3.907
H25	-0.555	-0.426	1.772	0.2637	1.905
H26	-1.305	-0.717	6.271	1.4163	6.445
H27	-1.046	-0.916	1.963	0.0003	2.406
H28	-0.696	-0.539	1.255	0.0067	1.533
H29	-1.179	-1.047	2.406	0.0600	2.877
H30	-0.535	-0.491	1.030	0.0013	1.260
H31	-0.136	-0.059	0.975	0.2600	0.986
H32	-0.470	-0.406	0.960	0.0280	1.144
H33	-0.471	-0.344	0.861	0.0153	1.040
H34	-0.467	-0.344	0.868	0.0190	1.044
H35	-0.491	-0.431	1.021	0.0330	1.212
H36	-0.083	0.000	1.067	0.3280	1.070
H37	-0.508	-0.468	0.982	0.0020	1.200
H38	-1.099	-0.970	2.154	0.0283	2.605
H39	-0.684	-0.530	1.226	0.0040	1.501
H40	-1.167	-0.984	2.332	0.0603	2.787
H41	-1.057	-0.379	6.517	1.6937	6.613
H42	-0.296	-0.170	2.060	0.5313	2.088
H43	-1.444	-1.270	2.799	0.0283	3.396
H44	-1.186	-0.462	3.683	0.6783	3.897
H45	-1.360	-0.592	3.617	0.5550	3.910
H46	-0.588	-0.477	1.612	0.1823	1.781
H47	-1.291	-0.773	5.530	1.1553	5.731
H48	-0.997	-0.885	1.886	0.0013	2.309
H49	-0.565	-0.522	1.134	0.0157	1.370
H50	-0.775	-0.606	1.418	0.0123	1.726
H51	-1.270	-1.153	2.955	0.1773	3.417
H52	-0.467	-0.398	0.956	0.0303	1.136
H53	-0.491	-0.358	0.880	0.0103	1.069
H54	-0.129	-0.044	1.006	0.2777	1.015
H55	-0.483	-0.352	0.856	0.0070	1.044
H56	-0.456	-0.393	0.914	0.0217	1.094
H57	-0.174	-0.099	0.908	0.2117	0.930

Table S3: SS-RASSCF(19,26) calculated hyperfine coupling tensors (in MHz) for the ^{1}H and ^{13}C nuclei in [ThCp$^{tt}_{3}$] (XRD structure).

Atom	A_1	A_2	A_3	A_{iso}	A_{Euclid}
H58	-1.187	-1.074	2.584	0.1077	3.040
H59	-0.752	-0.581	1.351	0.0060	1.652
H60	-0.524	-0.480	1.041	0.0123	1.261
H61	-0.479	-0.387	1.537	0.2237	1.655
H62	-1.004	-0.906	1.932	0.0073	2.358
H63	-1.157	-0.746	4.934	1.0103	5.123

Table S4: SA-CASSCF(1,12) calculated hyperfine coupling tensors (in MHz) for the ^{1}H and ^{13}C nuclei in [ThCp$^{tt}_{3}$] (XRD structure).

Atom	A_1	A_2	A_3	A_{iso}	A_{Euclid}
C1	-0.459	-0.571	1.141	0.037	1.356
C2	-1.008	-1.143	1.998	-0.051	2.513
C3	-0.422	-0.537	1.118	0.053	1.310
C4	-0.189	-0.808	1.095	0.033	1.373
C5	-0.199	-0.815	1.106	0.031	1.388
C6	1.373	1.535	2.498	1.802	3.238
C7	-0.060	-0.102	0.379	0.072	0.397
C8	0.582	0.662	1.428	0.891	1.678
C9	0.526	0.757	1.584	0.956	1.833
C10	1.162	1.306	2.245	1.571	2.846
C11	0.889	1.100	1.974	1.321	2.429
C12	-0.018	-0.063	0.410	0.110	0.415
C13	0.113	0.153	0.736	0.334	0.760
C14	-0.471	-0.568	1.053	0.005	1.286
C15	-1.050	-1.180	2.084	-0.049	2.615
C16	-0.489	-0.614	1.144	0.014	1.388
C17	-0.259	-0.855	1.165	0.017	1.468
C18	-0.116	-0.740	1.004	0.049	1.253
C19	0.981	1.132	2.026	1.380	2.520
C20	1.760	1.995	3.298	2.351	4.237
C21	0.086	0.116	0.688	0.297	0.703
C22	-0.045	-0.082	0.370	0.081	0.382
C23	1.268	1.442	2.427	1.712	3.095
C24	0.011	-0.049	0.440	0.134	0.443
C25	0.006	-0.017	0.545	0.178	0.545
C26	2.461	2.702	4.022	3.062	5.434
C27	-0.463	-0.586	1.169	0.040	1.387
C28	-0.982	-1.093	1.930	-0.048	2.426
C29	-0.356	-0.444	1.067	0.089	1.209
C30	-0.136	-0.770	1.062	0.052	1.319
C31	-0.236	-0.842	1.130	0.017	1.429
C32	1.271	1.433	2.388	1.697	3.061
C33	1.275	1.511	2.583	1.790	3.253
C34	0.273	0.329	0.987	0.530	1.075
C35	-0.039	-0.080	0.405	0.095	0.415
C36	1.034	1.173	2.079	1.429	2.601

Table S4: SA-CASSCF(1,12) calculated hyperfine coupling tensors (in MHz) for the ^{1}H and ^{13}C nuclei in [ThCp$^{tt}_3$] (XRD structure).

Atom	A_1	A_2	A_3	A_{iso}	A_{Euclid}
C37	-0.045	-0.088	0.363	0.077	0.376
C38	0.134	0.175	0.778	0.362	0.808
C39	0.880	1.100	1.989	1.323	2.437
H1	-1.147	-1.401	2.608	0.020	3.175
H2	-0.597	-0.997	3.319	0.575	3.516
H3	-0.585	-0.997	3.398	0.605	3.589
H4	-0.067	-0.162	0.905	0.225	0.922
H5	-0.373	-0.473	0.842	-0.001	1.036
H6	-0.383	-0.484	0.828	-0.013	1.033
H7	-0.688	-0.766	1.536	0.027	1.850
H8	-0.940	-1.215	3.320	0.388	3.658
H9	-0.443	-0.506	1.192	0.081	1.369
H10	-0.490	-0.614	1.282	0.059	1.504
H11	-0.755	-1.223	4.289	0.770	4.524
H12	-0.695	-0.753	1.541	0.031	1.851
H13	-0.341	-0.470	1.399	0.196	1.514
H14	-0.816	-0.884	1.735	0.012	2.111
H15	-0.550	-1.028	4.489	0.970	4.638
H16	-0.357	-0.471	0.779	-0.016	0.978
H17	-0.381	-0.468	0.846	-0.001	1.040
H18	-0.053	-0.149	0.881	0.226	0.895
H19	-0.437	-0.483	0.954	0.011	1.155
H20	-0.938	-1.060	2.295	0.099	2.697
H21	-0.570	-0.644	1.260	0.015	1.525
H22	-1.135	-1.428	2.610	0.016	3.185
H23	-0.690	-1.082	3.293	0.507	3.534
H24	-0.537	-0.909	3.413	0.656	3.572
H25	-0.346	-0.518	1.659	0.265	1.772
H26	-0.419	-1.072	5.923	1.477	6.034
H27	-0.854	-0.907	1.810	0.016	2.197
H28	-0.532	-0.606	1.182	0.015	1.431
H29	-0.962	-1.051	2.240	0.076	2.655
H30	-0.453	-0.486	0.968	0.010	1.174
H31	-0.039	-0.130	0.918	0.250	0.928
H32	-0.390	-0.469	0.874	0.005	1.066
H33	-0.347	-0.459	0.785	-0.007	0.973
H34	-0.340	-0.461	0.791	-0.003	0.977
H35	-0.411	-0.485	0.933	0.012	1.129
H36	0.019	-0.083	1.010	0.315	1.014
H37	-0.434	-0.463	0.926	0.010	1.122
H38	-0.907	-0.973	2.011	0.044	2.411
H39	-0.526	-0.601	1.153	0.009	1.402
H40	-0.930	-1.024	2.151	0.066	2.558
H41	-0.094	-0.824	6.189	1.757	6.244
H42	-0.100	-0.270	1.946	0.525	1.967
H43	-1.125	-1.369	2.539	0.015	3.096
H44	-0.541	-0.916	3.414	0.652	3.576
H45	-0.672	-1.072	3.338	0.531	3.569

Table S4: SA-CASSCF(1,12) calculated hyperfine coupling tensors (in MHz) for the ^{1}H and ^{13}C nuclei in [$ThCp^{tt}_3$] (XRD structure).

Atom	A_1	A_2	A_3	A_{iso}	A_{Euclid}
H46	-0.404	-0.552	1.506	0.183	1.654
H47	-0.515	-1.098	5.208	1.198	5.347
H48	-0.820	-0.875	1.738	0.014	2.111
H49	-0.474	-0.522	1.061	0.022	1.274
H50	-0.593	-0.668	1.338	0.026	1.608
H51	-1.003	-1.173	2.740	0.188	3.145
H52	-0.380	-0.474	0.868	0.005	1.060
H53	-0.364	-0.473	0.802	-0.012	1.000
H54	-0.030	-0.129	0.943	0.261	0.953
H55	-0.359	-0.469	0.785	-0.014	0.982
H56	-0.379	-0.464	0.834	-0.003	1.027
H57	-0.082	-0.172	0.854	0.200	0.875
H58	-0.965	-1.085	2.409	0.120	2.813
H59	-0.576	-0.651	1.280	0.018	1.548
H60	-0.444	-0.486	0.980	0.017	1.181
H61	-0.330	-0.461	1.434	0.214	1.542
H62	-0.832	-0.902	1.776	0.014	2.159
H63	-0.534	-1.013	4.634	1.029	4.773

Table S5: SA-RASSCF(19,27) calculated hyperfine coupling tensors (in MHz) for the ^{1}H and ^{13}C nuclei in [$ThCp^{tt}_3$] (XRD structure).

Atom	A_1	A_2	A_3	A_{iso}	A_{Euclid}
C1	-0.402	-0.717	-0.915	-0.678	1.230
C2	-1.334	-1.436	2.666	-0.035	3.309
C3	-0.434	-0.770	-1.011	-0.738	1.343
C4	0.124	-0.660	-0.841	-0.459	1.076
C5	-0.119	0.676	-0.850	-0.098	1.093
C6	1.457	1.606	2.574	1.879	3.366
C7	-0.080	-0.124	0.351	0.049	0.381
C8	0.660	0.738	1.528	0.975	1.821
C9	0.561	0.782	1.638	0.994	1.899
C10	1.253	1.386	2.326	1.655	2.983
C11	0.961	1.159	2.067	1.396	2.557
C12	-0.041	-0.088	0.378	0.083	0.390
C13	0.147	0.187	0.786	0.373	0.821
C14	-0.569	-0.856	-1.118	-0.848	1.518
C15	-1.401	-1.494	2.836	-0.020	3.499
C16	-0.512	-0.840	-0.957	-0.770	1.373
C17	-0.028	0.705	-0.894	-0.072	1.139
C18	0.203	-0.625	0.806	0.128	1.040
C19	1.054	1.193	2.087	1.445	2.625
C20	1.850	2.070	3.405	2.442	4.393
C21	0.116	0.147	0.732	0.332	0.755
C22	-0.069	-0.108	0.336	0.053	0.359
C23	1.358	1.522	2.509	1.796	3.233
C24	0.026	-0.063	0.421	0.128	0.427

Table S5: SA-RASSCF(19,27) calculated hyperfine coupling tensors (in MHz) for the ^{1}H and ^{13}C nuclei in [ThCp$^{tt}_3$] (XRD structure).

Atom	A_1	A_2	A_3	A_{iso}	A_{Euclid}
C25	-0.001	-0.025	0.575	0.183	0.576
C26	2.602	2.830	4.186	3.206	5.683
C27	-0.440	-0.755	-0.854	-0.683	1.222
C28	-1.306	-1.387	2.603	-0.030	3.226
C29	-0.348	-0.620	-1.004	-0.657	1.231
C30	0.169	0.645	-0.830	-0.005	1.064
C31	0.062	0.678	-0.848	-0.036	1.087
C32	1.354	1.505	2.467	1.775	3.191
C33	1.340	1.565	2.666	1.857	3.369
C34	0.325	0.379	1.058	0.587	1.170
C35	-0.055	-0.096	0.386	0.078	0.401
C36	1.118	1.245	2.152	1.505	2.726
C37	-0.070	-0.114	0.328	0.048	0.354
C38	0.174	0.216	0.836	0.409	0.880
C39	0.948	1.155	2.077	1.393	2.558
H1	-1.114	-1.570	2.845	0.054	3.435
H2	-0.521	-0.916	3.539	0.701	3.692
H3	-0.516	-0.901	3.620	0.734	3.766
H4	-0.063	-0.158	0.907	0.229	0.923
H5	-0.363	-0.460	0.812	-0.004	1.001
H6	-0.364	-0.483	0.824	-0.008	1.023
H7	-0.684	-0.799	1.571	0.029	1.891
H8	-0.929	-1.207	3.413	0.426	3.738
H9	-0.434	-0.494	1.226	0.099	1.391
H10	-0.497	-0.612	1.299	0.063	1.519
H11	-0.754	-1.201	4.372	0.806	4.596
H12	-0.722	-0.750	1.559	0.029	1.875
H13	-0.337	-0.457	1.430	0.212	1.539
H14	-0.842	-0.886	1.767	0.013	2.149
H15	-0.530	-0.987	4.603	1.029	4.737
H16	-0.340	-0.466	0.765	-0.014	0.958
H17	-0.371	-0.454	0.818	-0.002	1.006
H18	-0.051	-0.145	0.880	0.228	0.893
H19	-0.439	-0.482	0.974	0.018	1.172
H20	-0.947	-1.066	2.368	0.118	2.765
H21	-0.567	-0.668	1.286	0.017	1.557
H22	-1.085	-1.617	2.853	0.050	3.454
H23	-0.636	-1.000	3.513	0.626	3.707
H24	-0.451	-0.826	3.623	0.782	3.743
H25	-0.345	-0.506	1.687	0.279	1.795
H26	-0.391	-1.018	6.030	1.540	6.127
H27	-0.858	-0.933	1.834	0.014	2.229
H28	-0.526	-0.629	1.203	0.016	1.455
H29	-0.968	-1.062	2.305	0.092	2.716
H30	-0.456	-0.488	0.982	0.013	1.188
H31	-0.037	-0.127	0.915	0.250	0.924
H32	-0.381	-0.455	0.848	0.004	1.034
H33	-0.329	-0.453	0.767	-0.005	0.950

Table S5: SA-RASSCF(19,27) calculated hyperfine coupling tensors (in MHz) for the ^{1}H and ^{13}C nuclei in [ThCp$_3^{tt}$] (XRD structure).

Atom	A_1	A_2	A_3	A_{iso}	A_{Euclid}
H34	-0.325	-0.454	0.774	-0.002	0.954
H35	-0.406	-0.471	0.915	0.013	1.107
H36	-0.021	-0.078	1.016	0.306	1.019
H37	-0.439	-0.467	0.939	0.011	1.137
H38	-0.915	-0.990	2.072	0.056	2.472
H39	-0.519	-0.623	1.175	0.011	1.428
H40	-0.932	-1.050	2.196	0.071	2.606
H41	0.045	-0.760	6.327	1.871	6.372
H42	-0.087	-0.247	1.993	0.553	2.010
H43	-1.089	-1.541	2.769	0.046	3.351
H44	-0.456	-0.833	3.633	0.781	3.755
H45	-0.604	-0.989	3.555	0.654	3.739
H46	-0.405	-0.544	1.530	0.194	1.674
H47	-0.496	-1.058	5.302	1.249	5.430
H48	-0.837	-0.887	1.762	0.013	2.143
H49	-0.475	-0.521	1.084	0.029	1.293
H50	-0.592	-0.695	1.365	0.026	1.642
H51	-1.007	-1.176	2.822	0.213	3.219
H52	-0.373	-0.460	0.843	0.003	1.030
H53	-0.348	-0.471	0.795	-0.008	0.987
H54	-0.025	-0.123	0.948	0.267	0.957
H55	-0.341	-0.465	0.771	-0.012	0.962
H56	-0.368	-0.450	0.805	-0.004	0.993
H57	-0.079	-0.167	0.853	0.202	0.873
H58	-0.972	-1.091	2.489	0.142	2.886
H59	-0.572	-0.677	1.308	0.020	1.580
H60	-0.443	-0.484	1.001	0.025	1.197
H61	-0.327	-0.447	1.465	0.230	1.566
H62	-0.856	-0.906	1.808	0.015	2.196
H63	-0.515	-0.969	4.748	1.088	4.873

Table S6: SA-RASSCF(27,36) calculated hyperfine coupling tensors (in MHz) for the ^{1}H and ^{13}C nuclei in [ThCp$_3^{tt}$] (XRD structure).

Atom	A_1	A_2	A_3	A_{iso}	A_{Euclid}
C1	0.433	0.627	0.798	0.619	1.103
C2	1.149	1.258	2.395	1.601	2.939
C3	0.365	0.593	0.751	0.570	1.025
C4	0.073	0.721	0.906	0.567	1.160
C5	0.063	0.699	0.886	0.549	1.131
C6	1.296	1.440	2.418	1.718	3.098
C7	0.110	0.150	0.318	0.193	0.368
C8	0.502	0.570	1.350	0.807	1.549
C9	0.413	0.620	1.444	0.826	1.625
C10	1.129	1.257	2.212	1.533	2.783
C11	0.768	0.955	1.844	1.189	2.214
C12	0.086	0.128	0.328	0.181	0.363

Table S6: SA-RASSCF(27,36) calculated hyperfine coupling tensors (in MHz) for the ^{1}H and ^{13}C nuclei in [ThCp$^{tt}_{3}$] (XRD structure).

Atom	A_1	A_2	A_3	A_{iso}	A_{Euclid}
C13	0.078	0.112	0.715	0.302	0.728
C14	0.296	0.666	0.810	0.591	1.090
C15	1.200	1.299	2.532	1.677	3.088
C16	0.376	0.679	0.844	0.633	1.147
C17	0.153	0.781	0.969	0.634	1.254
C18	0.025	0.595	0.816	0.479	1.010
C19	0.942	1.076	1.984	1.334	2.445
C20	1.523	1.729	2.992	2.081	3.777
C21	0.054	0.082	0.669	0.268	0.676
C22	0.105	0.141	0.301	0.182	0.348
C23	1.225	1.383	2.384	1.664	3.017
C24	0.074	0.109	0.369	0.184	0.392
C25	0.020	0.040	0.537	0.199	0.539
C26	2.210	2.421	3.734	2.788	4.968
C27	0.475	0.636	0.797	0.636	1.124
C28	1.126	1.212	2.333	1.557	2.860
C29	0.334	0.516	0.672	0.507	0.911
C30	0.020	0.662	0.870	0.517	1.093
C31	0.115	0.740	0.915	0.590	1.182
C32	1.210	1.354	2.327	1.630	2.952
C33	1.081	1.292	2.344	1.572	2.887
C34	0.221	0.269	0.943	0.478	1.006
C35	0.093	0.132	0.340	0.188	0.377
C36	1.007	1.132	2.056	1.398	2.554
C37	0.106	0.146	0.294	0.182	0.345
C38	0.101	0.138	0.761	0.333	0.780
C39	0.762	0.956	1.860	1.193	2.226
H1	-1.181	-1.473	2.781	0.042	3.362
H2	-0.597	-0.989	3.502	0.639	3.688
H3	-0.591	-0.981	3.545	0.658	3.725
H4	-0.110	-0.194	0.878	0.191	0.906
H5	-0.383	-0.473	0.846	-0.003	1.043
H6	-0.384	-0.491	0.845	-0.010	1.050
H7	-0.700	-0.804	1.573	0.023	1.900
H8	-1.017	-1.254	3.363	0.364	3.731
H9	-0.461	-0.514	1.207	0.077	1.390
H10	-0.514	-0.619	1.287	0.051	1.517
H11	-0.874	-1.274	4.267	0.706	4.538
H12	-0.729	-0.764	1.570	0.026	1.892
H13	-0.377	-0.486	1.411	0.183	1.539
H14	-0.860	-0.907	1.790	0.008	2.183
H15	-0.679	-1.095	4.544	0.923	4.723
H16	-0.361	-0.474	0.794	-0.014	0.993
H17	-0.394	-0.472	0.852	-0.005	1.051
H18	-0.105	-0.189	0.849	0.185	0.876
H19	-0.455	-0.492	0.977	0.010	1.185
H20	-0.994	-1.093	2.373	0.095	2.796
H21	-0.583	-0.674	1.299	0.014	1.575

Table S6: SA-RASSCF(27,36) calculated hyperfine coupling tensors (in MHz) for the ^{1}H and ^{13}C nuclei in [ThCp$^{tt}_{3}$] (XRD structure).

Atom	A_1	A_2	A_3	A_{iso}	A_{Euclid}
H22	-1.158	-1.510	2.790	0.041	3.377
H23	-0.696	-1.083	3.471	0.564	3.702
H24	-0.546	-0.897	3.551	0.703	3.703
H25	-0.387	-0.533	1.647	0.242	1.774
H26	-0.578	-1.152	5.866	1.379	6.006
H27	-0.874	-0.937	1.839	0.009	2.242
H28	-0.541	-0.631	1.211	0.013	1.469
H29	-1.003	-1.082	2.299	0.071	2.732
H30	-0.467	-0.496	0.984	0.007	1.197
H31	-0.087	-0.168	0.884	0.210	0.904
H32	-0.400	-0.472	0.877	0.002	1.074
H33	-0.351	-0.462	0.794	-0.006	0.983
H34	-0.348	-0.463	0.802	-0.003	0.990
H35	-0.427	-0.492	0.944	0.008	1.147
H36	-0.034	-0.122	0.976	0.273	0.984
H37	-0.450	-0.474	0.947	0.008	1.151
H38	-0.945	-1.008	2.078	0.042	2.496
H39	-0.535	-0.626	1.188	0.009	1.445
H40	-0.969	-1.076	2.210	0.055	2.642
H41	-0.267	-0.923	6.234	1.681	6.308
H42	-0.156	-0.302	1.942	0.495	1.971
H43	-1.153	-1.444	2.710	0.038	3.280
H44	-0.549	-0.914	3.581	0.706	3.736
H45	-0.665	-1.053	3.505	0.596	3.720
H46	-0.438	-0.563	1.502	0.167	1.663
H47	-0.659	-1.168	5.173	1.115	5.344
H48	-0.849	-0.898	1.773	0.009	2.161
H49	-0.491	-0.530	1.078	0.019	1.298
H50	-0.605	-0.698	1.369	0.022	1.652
H51	-1.065	-1.202	2.794	0.176	3.222
H52	-0.393	-0.475	0.873	0.002	1.069
H53	-0.367	-0.477	0.817	-0.009	1.015
H54	-0.078	-0.165	0.911	0.223	0.929
H55	-0.362	-0.473	0.799	-0.012	0.997
H56	-0.389	-0.466	0.840	-0.005	1.036
H57	-0.125	-0.204	0.830	0.167	0.864
H58	-1.021	-1.120	2.493	0.117	2.917
H59	-0.588	-0.682	1.319	0.016	1.598
H60	-0.460	-0.496	1.004	0.016	1.211
H61	-0.367	-0.478	1.444	0.200	1.564
H62	-0.875	-0.927	1.830	0.009	2.230
H63	-0.669	-1.085	4.679	0.975	4.850

Table S7: SA-RASCI(29,35) calculated hyperfine coupling tensors (in MHz) for the ^{1}H nuclei in [UCp_3^{tt}] (XRD structure).

Atom	A_1	A_2	A_3	A_{iso}	A_{Euclid}
H1	-1.114	-1.570	2.845	0.054	3.435
H2	-0.521	-0.916	3.539	0.701	3.692
H3	-0.516	-0.901	3.620	0.734	3.766
H4	-0.063	-0.158	0.907	0.229	0.923
H5	-0.363	-0.460	0.812	-0.004	1.001
H6	-0.364	-0.483	0.824	-0.008	1.023
H7	-0.684	-0.799	1.571	0.029	1.891
H8	-0.929	-1.207	3.413	0.426	3.738
H9	-0.434	-0.494	1.226	0.099	1.391
H10	-0.497	-0.612	1.299	0.063	1.519
H11	-0.754	-1.201	4.372	0.806	4.596
H12	-0.722	-0.750	1.559	0.029	1.875
H13	-0.337	-0.457	1.430	0.212	1.539
H14	-0.842	-0.886	1.767	0.013	2.149
H15	-0.530	-0.987	4.603	1.029	4.737
H16	-0.340	-0.466	0.765	-0.014	0.958
H17	-0.371	-0.454	0.818	-0.002	1.006
H18	-0.051	-0.145	0.880	0.228	0.893
H19	-0.439	-0.482	0.974	0.018	1.172
H20	-0.947	-1.066	2.368	0.118	2.765
H21	-0.567	-0.668	1.286	0.017	1.557
H22	-1.085	-1.617	2.853	0.050	3.454
H23	-0.636	-1.000	3.513	0.626	3.707
H24	-0.451	-0.826	3.623	0.782	3.743
H25	-0.345	-0.506	1.687	0.279	1.795
H26	-0.391	-1.018	6.030	1.540	6.127
H27	-0.858	-0.933	1.834	0.014	2.229
H28	-0.526	-0.629	1.203	0.016	1.455
H29	-0.968	-1.062	2.305	0.092	2.716
H30	-0.456	-0.488	0.982	0.013	1.188
H31	-0.037	-0.127	0.915	0.250	0.924
H32	-0.381	-0.455	0.848	0.004	1.034
H33	-0.329	-0.453	0.767	-0.005	0.950
H34	-0.325	-0.454	0.774	-0.002	0.954
H35	-0.406	-0.471	0.915	0.013	1.107
H36	-0.021	-0.078	1.016	0.306	1.019
H37	-0.439	-0.467	0.939	0.011	1.137
H38	-0.915	-0.990	2.072	0.056	2.472
H39	-0.519	-0.623	1.175	0.011	1.428
H40	-0.932	-1.050	2.196	0.071	2.606
H41	0.045	-0.760	6.327	1.871	6.372
H42	-0.087	-0.247	1.993	0.553	2.010
H43	-1.089	-1.541	2.769	0.046	3.351
H44	-0.456	-0.833	3.633	0.781	3.755
H45	-0.604	-0.989	3.555	0.654	3.739
H46	-0.405	-0.544	1.530	0.194	1.674
H47	-0.496	-1.058	5.302	1.249	5.430
H48	-0.837	-0.887	1.762	0.013	2.143

Table S7: SA-RASCI(29,35) calculated hyperfine coupling tensors (in MHz) for the ^{1}H nuclei in [UCp^{tt}_3] (XRD structure).

Atom	A_1	A_2	A_3	A_{iso}	A_{Euclid}
H49	-0.475	-0.521	1.084	0.029	1.293
H50	-0.592	-0.695	1.365	0.026	1.642
H51	-1.007	-1.176	2.822	0.213	3.219
H52	-0.373	-0.460	0.843	0.003	1.030
H53	-0.348	-0.471	0.795	-0.008	0.987
H54	-0.025	-0.123	0.948	0.267	0.957
H55	-0.341	-0.465	0.771	-0.012	0.962
H56	-0.368	-0.450	0.805	-0.004	0.993
H57	-0.079	-0.167	0.853	0.202	0.873
H58	-0.972	-1.091	2.489	0.142	2.886
H59	-0.572	-0.677	1.308	0.020	1.580
H60	-0.443	-0.484	1.001	0.025	1.197
H61	-0.327	-0.447	1.465	0.230	1.566
H62	-0.856	-0.906	1.808	0.015	2.196
H63	-0.515	-0.969	4.748	1.088	4.873

Table S8: SA-RASCI(29,34) calculated hyperfine coupling tensors (in MHz) for the ^{1}H nuclei in [UCp^{tt}_3] (XRD structure).

Atom	A_1	A_2	A_3	A_{iso}	$\sqrt{A_1^2 + A_2^2 + A_3^2}$
H1	0.415	2.508	-6.590	-1.222	7.063
H2	0.653	4.569	-5.843	-0.207	7.446
H3	0.641	4.691	-5.935	-0.201	7.593
H4	-0.439	-1.924	2.749	0.129	3.384
H5	-0.242	0.898	-1.557	-0.300	1.814
H6	0.633	-2.227	-4.256	-1.950	4.845
H7	0.228	1.236	-3.436	-0.657	3.658
H8	0.118	0.604	-1.489	-0.256	1.611
H9	0.132	0.783	-2.017	-0.367	2.167
H10	0.109	0.733	-1.308	-0.155	1.503
H11	0.115	0.617	-0.986	-0.085	1.169
H12	0.150	-1.069	-1.379	-0.766	1.751
H13	-0.174	-0.774	1.172	0.075	1.416
H14	0.240	-1.417	1.698	0.174	2.225
H15	-0.503	-1.875	2.903	0.175	3.492
H16	0.167	0.929	-2.439	-0.448	2.615
H17	0.115	0.576	-1.326	-0.212	1.450
H18	0.252	1.167	-2.872	-0.484	3.110
H19	0.105	0.698	-1.320	-0.172	1.497
H20	-0.125	0.930	-1.265	-0.153	1.576
H21	-0.103	0.573	-0.908	-0.146	1.079
H22	0.402	2.434	-7.342	-1.502	7.745
H23	0.572	3.457	-7.804	-1.258	8.555
H24	0.562	3.434	-7.667	-1.224	8.419
H25	0.441	-1.389	2.922	0.658	3.266
H26	0.230	-0.961	-2.246	-0.992	2.453
H27	-0.163	0.567	-1.351	-0.316	1.474

Table S8: SA-RASCI(29,34) calculated hyperfine coupling tensors (in MHz) for the ^{1}H nuclei in [UCp$^{tt}_{3}$] (XRD structure).

Atom	A_1	A_2	A_3	A_{iso}	$\sqrt{A_1^2 + A_2^2 + A_3^2}$
H28	-0.277	1.344	-2.744	-0.559	3.067
H29	0.123	0.559	-1.404	-0.241	1.516
H30	0.175	0.948	-2.541	-0.473	2.718
H31	0.092	0.412	-1.203	-0.233	1.275
H32	0.120	0.569	-1.868	-0.393	1.957
H33	0.106	0.528	-1.860	-0.409	1.937
H34	-0.361	1.280	-3.038	-0.706	3.317
H35	0.138	0.544	-1.484	-0.267	1.587
H36	0.198	0.897	-2.452	-0.452	2.618
H37	0.185	1.065	-2.818	-0.523	3.018
H38	0.124	0.615	-1.581	-0.281	1.701
H39	0.306	1.542	-3.287	-0.480	3.644
H40	0.107	0.543	-1.929	-0.426	2.007
H41	0.081	0.420	-1.301	-0.267	1.369
H42	0.101	0.556	-1.997	-0.447	2.075
H43	0.425	-3.815	-4.457	-2.616	5.882
H44	0.635	3.233	-8.257	-1.463	8.890
H45	0.569	3.196	-8.834	-1.690	9.412
H46	0.203	0.956	-2.356	-0.399	2.551
H47	0.137	0.647	-1.231	-0.149	1.397
H48	-0.366	1.445	-2.696	-0.539	3.080
H49	0.269	-1.852	2.292	0.236	2.959
H50	-0.114	0.845	1.010	0.580	1.322
H51	-0.163	1.512	1.581	0.977	2.194
H52	0.100	0.741	-1.401	-0.187	1.588
H53	0.090	0.526	-0.994	-0.126	1.129
H54	0.108	0.672	-1.624	-0.281	1.761
H55	-0.429	1.473	2.562	1.202	2.986
H56	-0.158	0.661	1.162	0.555	1.346
H57	-0.241	0.995	-2.277	-0.508	2.496
H58	0.160	-1.408	1.466	0.073	2.039
H59	-0.127	0.863	1.017	0.584	1.340
H60	-0.296	1.875	-2.597	-0.339	3.217
H61	0.118	0.685	-1.585	-0.261	1.731
H62	0.090	0.535	-0.944	-0.106	1.088
H63	0.103	0.722	-1.304	-0.160	1.494

Table S9: SA-RASSCF(21,30) calculated hyperfine coupling tensors (in MHz) for the ^{1}H nuclei in [UCp$^{tt}_{3}$] (XRD structure).

Atom	A_1	A_2	A_3	A_{iso}	A_{Euclid}
H1	0.371	2.077	-6.742	-1.431	7.064
H2	0.614	-4.022	-5.733	-3.047	7.030
H3	0.567	-3.987	-5.912	-3.111	7.153
H4	-0.387	-1.578	2.816	0.284	3.251
H5	-0.214	0.743	-1.622	-0.364	1.797
H6	0.551	-1.828	-4.417	-1.898	4.812

Table S9: SA-RASSCF(21,30) calculated hyperfine coupling tensors (in MHz) for the ^{1}H nuclei in [UCp_3^{tt}] (XRD structure).

Atom	A_1	A_2	A_3	A_{iso}	A_{Euclid}
H7	0.197	1.021	-3.563	-0.782	3.712
H8	0.103	0.498	-1.548	-0.316	1.630
H9	0.115	0.649	-2.086	-0.441	2.188
H10	0.097	0.619	-1.326	-0.203	1.466
H11	0.102	-0.515	-1.016	-0.476	1.143
H12	0.134	-0.905	1.390	0.206	1.664
H13	-0.155	-0.647	1.193	0.130	1.366
H14	0.212	-1.184	1.739	0.256	2.114
H15	-0.447	-1.561	2.954	0.315	3.371
H16	0.150	0.769	-2.487	-0.523	2.608
H17	0.103	0.479	-1.347	-0.255	1.433
H18	0.226	0.963	-2.910	-0.574	3.074
H19	0.095	0.595	-1.324	-0.211	1.454
H20	-0.111	0.810	-1.245	-0.182	1.489
H21	-0.094	0.488	-0.913	-0.173	1.040
H22	0.346	2.037	-7.378	-1.665	7.662
H23	0.506	2.866	-8.058	-1.562	8.568
H24	0.471	2.809	-7.818	-1.513	8.321
H25	0.391	-1.144	2.943	0.730	3.182
H26	0.204	-0.794	-2.272	-0.954	2.416
H27	-0.146	0.468	-1.363	-0.347	1.448
H28	-0.247	1.134	-2.707	-0.607	2.945
H29	0.110	0.468	-1.399	-0.274	1.479
H30	0.156	0.798	-2.525	-0.524	2.653
H31	0.081	0.342	-1.216	-0.264	1.266
H32	0.108	0.469	-1.906	-0.443	1.966
H33	0.094	0.436	-1.893	-0.454	1.945
H34	0.316	-1.054	-3.153	-1.297	3.340
H35	0.121	0.449	-1.541	-0.324	1.610
H36	0.175	0.737	-2.548	-0.545	2.658
H37	0.163	0.904	-2.836	-0.590	2.981
H38	0.109	0.518	-1.611	-0.328	1.696
H39	0.269	1.308	-3.326	-0.583	3.584
H40	0.095	0.447	-1.993	-0.484	2.045
H41	0.068	0.349	-1.337	-0.307	1.384
H42	0.088	0.461	-2.048	-0.500	2.101
H43	0.386	-3.422	4.238	0.401	5.461
H44	0.579	2.713	-8.281	-1.663	8.734
H45	0.472	2.642	-9.003	-1.963	9.394
H46	0.175	0.813	-2.386	-0.466	2.526
H47	0.120	0.555	-1.235	-0.187	1.359
H48	-0.317	1.223	-2.734	-0.609	3.012
H49	0.236	-1.544	2.333	0.342	2.807
H50	-0.101	-0.724	1.008	0.061	1.245
H51	0.144	-1.286	1.585	0.148	2.046
H52	0.088	-0.669	-1.332	-0.638	1.493
H53	0.080	0.464	-0.969	-0.142	1.078
H54	0.093	0.589	-1.593	-0.304	1.701

Table S9: SA-RASSCF(21,30) calculated hyperfine coupling tensors (in MHz) for the ^{1}H nuclei in [UCp$^{tt}_{3}$] (XRD structure).

Atom	A_1	A_2	A_3	A_{iso}	A_{Euclid}
H55	-0.384	1.231	2.557	1.135	2.864
H56	-0.141	-0.563	1.147	0.148	1.286
H57	-0.216	0.839	-2.249	-0.542	2.410
H58	0.143	-1.159	1.511	0.165	1.910
H59	-0.114	0.713	1.044	0.548	1.269
H60	-0.264	1.539	2.679	1.318	3.101
H61	0.104	0.599	-1.534	-0.277	1.650
H62	0.080	0.468	-0.910	-0.121	1.026
H63	0.091	-0.651	-1.227	-0.596	1.392

Table S10: SA-RASSCF(3,7) calculated hyperfine coupling tensors (in MHz) for the ^{1}H nuclei in [UCp$^{tt}_{3}$] (XRD structure).

Atom	A_1	A_2	A_3	A_{iso}	A_{Euclid}
H1	0.058	0.383	-3.941	-1.167	3.960
H2	0.125	-0.481	-3.872	-1.409	3.904
H3	-0.138	-0.479	3.503	0.962	3.538
H4	-0.055	0.209	1.847	0.667	1.859
H5	-0.030	0.099	-1.136	-0.356	1.141
H6	0.057	0.289	-3.198	-0.951	3.211
H7	0.024	0.183	-2.391	-0.728	2.398
H8	0.017	0.087	-1.041	-0.312	1.045
H9	0.023	0.115	-1.308	-0.390	1.314
H10	-0.020	0.105	-0.783	-0.233	0.790
H11	0.020	0.071	-0.650	-0.186	0.654
H12	0.024	-0.136	0.849	0.246	0.861
H13	0.020	-0.125	0.678	0.191	0.689
H14	0.033	-0.199	1.168	0.334	1.186
H15	0.056	-0.283	1.797	0.523	1.820
H16	0.019	0.151	-1.306	-0.379	1.315
H17	-0.014	0.098	-0.676	-0.197	0.683
H18	0.023	0.185	-1.487	-0.426	1.499
H19	-0.020	0.121	0.660	0.254	0.671
H20	0.024	-0.152	0.665	0.179	0.683
H21	0.019	-0.088	0.465	0.132	0.474
H22	-0.026	0.399	-4.447	-1.358	4.465
H23	0.092	0.434	-5.154	-1.543	5.173
H24	0.062	0.723	-4.277	-1.164	4.338
H25	-0.009	-0.231	1.598	0.453	1.615
H26	-0.003	-0.165	1.160	0.331	1.172
H27	0.002	-0.088	0.697	0.204	0.703
H28	-0.018	0.169	1.437	0.529	1.447
H29	-0.006	0.072	-0.731	-0.222	0.734
H30	-0.006	0.129	-1.378	-0.418	1.384
H31	-0.006	0.056	-0.633	-0.194	0.635
H32	-0.006	0.085	-1.004	-0.308	1.007
H33	-0.005	0.076	-1.028	-0.319	1.031

Table S10: SA-RASSCF(3,7) calculated hyperfine coupling tensors (in MHz) for the ^{1}H nuclei in [UCp$^{tt}_{3}$] (XRD structure).

Atom	A_1	A_2	A_3	A_{iso}	A_{Euclid}
H34	0.014	0.168	-2.273	-0.697	2.280
H35	0.004	0.071	-1.085	-0.337	1.087
H36	0.007	0.112	-1.723	-0.535	1.727
H37	0.007	0.145	-1.899	-0.582	1.904
H38	0.006	0.082	-1.124	-0.345	1.127
H39	0.020	0.204	-2.385	-0.720	2.393
H40	-0.002	0.076	-1.242	-0.389	1.244
H41	-0.006	0.058	-0.880	-0.276	0.882
H42	-0.002	0.078	-1.269	-0.398	1.272
H43	-0.049	0.463	2.667	1.027	2.707
H44	-0.014	0.503	-4.698	-1.403	4.725
H45	-0.097	0.540	-6.011	-1.856	6.036
H46	0.008	0.147	-1.699	-0.515	1.705
H47	0.008	0.097	-0.890	-0.262	0.896
H48	0.019	0.215	-2.009	-0.592	2.020
H49	0.019	-0.280	1.416	0.385	1.444
H50	-0.007	0.119	0.667	0.260	0.678
H51	0.015	-0.199	1.006	0.274	1.026
H52	-0.004	0.114	-0.829	-0.240	0.837
H53	0.000	0.087	-0.636	-0.183	0.642
H54	0.003	0.106	-1.021	-0.304	1.027
H55	0.029	-0.190	1.505	0.448	1.517
H56	-0.012	0.086	0.641	0.238	0.646
H57	-0.021	0.143	1.178	0.433	1.186
H58	0.008	-0.156	0.961	0.271	0.973
H59	-0.006	-0.094	0.674	0.191	0.681
H60	0.022	0.204	-1.773	-0.516	1.785
H61	0.008	0.106	-0.831	-0.239	0.838
H62	0.006	-0.081	0.495	0.140	0.502
H63	0.005	-0.111	0.679	0.191	0.688

Table S11: SA-CASSCF(1,12) calculated hyperfine coupling tensors (in MHz) for the ^{13}C and ^{1}H nuclei in [ThCp$^{tt}_{3}$] (Optimised structure).

Atom	A_1	A_2	A_3	A_{iso}	A_{Euclid}
C1	-0.431	-0.637	1.057	-0.004	1.307
C2	-1.044	-1.156	1.964	-0.079	2.507
C3	-0.432	-0.638	1.058	-0.004	1.309
C4	-0.233	-0.667	1.043	0.048	1.260
C5	-0.232	-0.666	1.042	0.048	1.259
C6	-0.431	-0.638	1.059	-0.003	1.309
C7	-1.046	-1.157	1.967	-0.079	2.510
C8	-0.431	-0.638	1.059	-0.003	1.309
C9	-0.231	-0.666	1.043	0.049	1.259
C10	-0.233	-0.668	1.045	0.048	1.262
C11	-0.433	-0.639	1.062	-0.003	1.313
C12	-1.050	-1.161	1.975	-0.079	2.520

Table S11: SA-CASSCF(1,12) calculated hyperfine coupling tensors (in MHz) for the ^{13}C and ^{1}H nuclei in [ThCp$^{tt}_{3}$] (Optimised structure).

Atom	A_1	A_2	A_3	A_{iso}	A_{Euclid}
C13	-0.434	-0.639	1.062	-0.004	1.313
C14	-0.232	-0.667	1.044	0.048	1.261
C15	-0.234	-0.669	1.047	0.048	1.264
C16	0.957	1.105	2.022	1.361	2.495
C17	0.961	1.108	2.026	1.365	2.501
C18	0.965	1.112	2.031	1.369	2.508
C19	0.964	1.111	2.030	1.368	2.507
C20	0.974	1.121	2.042	1.379	2.525
C21	0.973	1.120	2.041	1.378	2.523
C22	-0.037	-0.064	0.366	0.088	0.373
C23	0.133	0.179	0.776	0.363	0.808
C24	1.292	1.563	2.651	1.835	3.337
C25	1.286	1.557	2.644	1.829	3.327
C26	-0.037	-0.065	0.366	0.088	0.374
C27	0.136	0.182	0.781	0.366	0.813
C28	1.270	1.540	2.619	1.810	3.293
C29	0.137	0.183	0.782	0.367	0.815
C30	-0.036	-0.064	0.367	0.089	0.374
C31	-0.036	-0.064	0.368	0.089	0.375
C32	0.136	0.182	0.780	0.366	0.813
C33	1.271	1.541	2.622	1.811	3.296
C34	1.251	1.521	2.591	1.788	3.255
C35	0.144	0.191	0.792	0.376	0.827
C36	-0.036	-0.064	0.368	0.089	0.375
C37	-0.036	-0.064	0.369	0.090	0.376
C38	0.142	0.189	0.790	0.374	0.824
C39	1.259	1.530	2.605	1.798	3.273
H1	-0.998	-1.259	2.246	-0.004	2.761
H2	-0.659	-0.899	2.905	0.449	3.111
H3	-0.658	-0.898	2.903	0.449	3.110
H4	-0.998	-1.260	2.248	-0.003	2.763
H5	-0.657	-0.897	2.906	0.451	3.111
H6	-0.659	-0.899	2.904	0.449	3.111
H7	-0.998	-1.263	2.250	-0.004	2.767
H8	-0.657	-0.897	2.912	0.453	3.117
H9	-0.661	-0.901	2.906	0.448	3.114
H10	0.022	0.083	0.937	0.347	0.941
H11	-0.366	-0.433	0.787	-0.004	0.970
H12	-0.341	-0.417	0.742	-0.005	0.917
H13	-0.536	-0.583	1.207	0.029	1.443
H14	-0.985	-1.118	2.487	0.128	2.899
H15	-0.403	-0.445	0.919	0.024	1.097
H16	-0.214	-0.340	1.461	0.302	1.516
H17	-0.428	-1.013	5.209	1.256	5.324
H18	-0.745	-0.761	1.609	0.034	1.930
H19	-0.217	-0.342	1.459	0.300	1.514
H20	-0.743	-0.759	1.607	0.035	1.926
H21	-0.430	-1.015	5.204	1.253	5.319

Table S11: SA-CASSCF(1,12) calculated hyperfine coupling tensors (in MHz) for the ^{13}C and ^{1}H nuclei in [ThCp$^{tt}_3$] (Optimised structure).

Atom	A_1	A_2	A_3	A_{iso}	A_{Euclid}
H22	-0.341	-0.417	0.742	-0.005	0.917
H23	-0.366	-0.433	0.787	-0.004	0.970
H24	0.022	0.084	0.938	0.348	0.942
H25	-0.403	-0.445	0.920	0.024	1.098
H26	-0.986	-1.119	2.493	0.129	2.905
H27	-0.536	-0.583	1.209	0.030	1.445
H28	-0.219	-0.343	1.453	0.297	1.509
H29	-0.434	-1.014	5.181	1.244	5.297
H30	-0.743	-0.758	1.604	0.034	1.924
H31	-0.536	-0.583	1.209	0.030	1.445
H32	-0.986	-1.120	2.495	0.130	2.907
H33	-0.403	-0.445	0.920	0.024	1.099
H34	0.022	0.083	0.938	0.348	0.942
H35	-0.366	-0.433	0.787	-0.004	0.970
H36	-0.342	-0.417	0.742	-0.006	0.917
H37	-0.342	-0.417	0.742	-0.006	0.917
H38	-0.366	-0.433	0.787	-0.004	0.970
H39	0.022	0.083	0.937	0.347	0.941
H40	-0.403	-0.444	0.919	0.024	1.097
H41	-0.983	-1.117	2.488	0.129	2.899
H42	-0.537	-0.584	1.210	0.030	1.447
H43	-0.742	-0.758	1.604	0.035	1.923
H44	-0.434	-1.015	5.184	1.245	5.300
H45	-0.220	-0.344	1.453	0.296	1.509
H46	-0.224	-0.347	1.446	0.292	1.504
H47	-0.439	-1.017	5.156	1.233	5.273
H48	-0.740	-0.755	1.600	0.035	1.918
H49	-0.403	-0.446	0.923	0.025	1.101
H50	-0.538	-0.585	1.213	0.030	1.450
H51	-0.987	-1.124	2.508	0.132	2.920
H52	-0.366	-0.433	0.787	-0.004	0.970
H53	-0.342	-0.419	0.743	-0.006	0.919
H54	0.022	0.084	0.938	0.348	0.942
H55	-0.342	-0.418	0.743	-0.006	0.919
H56	-0.366	-0.433	0.787	-0.004	0.970
H57	0.022	0.084	0.939	0.348	0.943
H58	-0.986	-1.122	2.503	0.132	2.915
H59	-0.537	-0.585	1.212	0.030	1.449
H60	-0.403	-0.445	0.922	0.025	1.101
H61	-0.222	-0.347	1.449	0.293	1.507
H62	-0.741	-0.756	1.602	0.035	1.920
H63	-0.434	-1.015	5.170	1.240	5.287

Table S12: SS-RASSCF(19,26) calculated hyperfine coupling tensors (in MHz) for the ^{13}C and ^{1}H nuclei in [ThCp$^{tt}_3$] (Optimised structure).

Atom	A_1	A_2	A_3	A_{iso}	A_{Euclid}
C1	-0.933	-0.642	0.673	-0.301	1.318
C2	-1.205	-1.069	2.361	0.029	2.858
C3	-0.897	-0.640	0.597	-0.313	1.253
C4	-0.777	-0.078	0.776	-0.026	1.100
C5	-0.764	-0.072	0.766	-0.023	1.084
C6	1.287	1.427	2.454	1.723	3.117
C7	-0.138	-0.102	0.354	0.038	0.393
C8	0.523	0.587	1.395	0.835	1.601
C9	0.504	0.699	1.580	0.928	1.799
C10	1.101	1.227	2.220	1.516	2.765
C11	0.859	1.025	1.962	1.282	2.375
C12	-0.109	-0.076	0.371	0.062	0.394
C13	0.075	0.106	0.732	0.304	0.743
C14	-0.940	-0.692	0.526	-0.369	1.281
C15	-1.235	-1.110	2.487	0.047	2.990
C16	-0.973	-0.706	0.617	-0.354	1.351
C17	-0.798	-0.149	0.887	-0.020	1.203
C18	-0.729	0.010	0.631	-0.029	0.964
C19	0.918	1.052	2.005	1.325	2.443
C20	1.698	1.879	3.201	2.259	4.082
C21	0.047	0.075	0.685	0.269	0.691
C22	-0.129	-0.098	0.339	0.037	0.376
C23	1.203	1.359	2.401	1.654	3.010
C24	-0.099	-0.071	0.404	0.078	0.422
C25	-0.047	-0.025	0.554	0.161	0.556
C26	2.388	2.567	3.933	2.963	5.269
C27	-0.930	-0.661	0.744	-0.282	1.362
C28	-1.157	-1.044	2.290	0.030	2.770
C29	-0.814	-0.549	0.544	-0.273	1.122
C30	-0.763	-0.029	0.708	-0.028	1.041
C31	-0.787	-0.116	0.808	-0.032	1.133
C32	1.190	1.333	2.350	1.624	2.952
C33	1.223	1.416	2.525	1.721	3.142
C34	0.231	0.270	0.974	0.492	1.037
C35	-0.116	-0.084	0.381	0.060	0.407
C36	0.980	1.101	2.067	1.383	2.538
C37	-0.132	-0.100	0.330	0.033	0.369
C38	0.095	0.129	0.773	0.332	0.789
C39	0.855	1.030	1.984	1.290	2.393
H1	-1.199	-1.317	2.587	0.024	3.141
H2	-0.572	-1.144	3.245	0.510	3.488
H3	-0.571	-1.143	3.243	0.510	3.486
H4	-1.205	-1.307	2.579	0.022	3.132
H5	-0.570	-1.136	3.264	0.519	3.503
H6	-0.572	-1.138	3.263	0.518	3.502
H7	-1.198	-1.318	2.588	0.024	3.141
H8	-0.575	-1.150	3.247	0.507	3.492
H9	-0.578	-1.154	3.241	0.503	3.489

Table S12: SS-RASSCF(19,26) calculated hyperfine coupling tensors (in MHz) for the ^{13}C and ^{1}H nuclei in [ThCp$^{tt}_3$] (Optimised structure).

Atom	A_1	A_2	A_3	A_{iso}	A_{Euclid}
H10	0.017	0.082	1.051	0.383	1.054
H11	-0.380	-0.443	0.901	0.026	1.073
H12	-0.333	-0.454	0.855	0.023	1.024
H13	-0.554	-0.705	1.317	0.019	1.593
H14	-1.160	-1.262	2.748	0.109	3.239
H15	-0.455	-0.487	1.007	0.022	1.208
H16	-0.265	-0.363	1.609	0.327	1.671
H17	-0.806	-1.370	5.581	1.135	5.803
H18	-0.814	-0.925	1.768	0.010	2.155
H19	-0.268	-0.366	1.606	0.324	1.669
H20	-0.812	-0.923	1.766	0.010	2.151
H21	-0.808	-1.371	5.576	1.132	5.799
H22	-0.333	-0.454	0.855	0.023	1.024
H23	-0.380	-0.443	0.901	0.026	1.073
H24	0.017	0.082	1.051	0.383	1.054
H25	-0.455	-0.487	1.008	0.022	1.209
H26	-1.161	-1.263	2.755	0.110	3.246
H27	-0.555	-0.706	1.319	0.019	1.596
H28	-0.269	-0.368	1.604	0.322	1.668
H29	-0.797	-1.369	5.566	1.133	5.787
H30	-0.816	-0.924	1.769	0.010	2.156
H31	-0.554	-0.703	1.315	0.019	1.591
H32	-1.166	-1.265	2.745	0.105	3.240
H33	-0.459	-0.489	1.004	0.019	1.208
H34	0.017	0.082	1.053	0.384	1.057
H35	-0.380	-0.445	0.909	0.028	1.082
H36	-0.336	-0.456	0.852	0.020	1.023
H37	-0.336	-0.456	0.852	0.020	1.023
H38	-0.380	-0.445	0.909	0.028	1.082
H39	0.016	0.081	1.053	0.383	1.056
H40	-0.458	-0.489	1.003	0.019	1.206
H41	-1.162	-1.261	2.738	0.105	3.230
H42	-0.555	-0.705	1.316	0.019	1.593
H43	-0.815	-0.923	1.769	0.010	2.155
H44	-0.797	-1.370	5.570	1.134	5.791
H45	-0.270	-0.369	1.604	0.322	1.668
H46	-0.274	-0.371	1.591	0.315	1.657
H47	-0.811	-1.370	5.524	1.114	5.748
H48	-0.810	-0.916	1.758	0.011	2.141
H49	-0.455	-0.487	1.009	0.022	1.209
H50	-0.555	-0.707	1.320	0.019	1.597
H51	-1.161	-1.267	2.762	0.111	3.252
H52	-0.379	-0.442	0.900	0.026	1.072
H53	-0.334	-0.456	0.855	0.022	1.025
H54	0.016	0.081	1.049	0.382	1.052
H55	-0.334	-0.456	0.855	0.022	1.025
H56	-0.380	-0.443	0.900	0.026	1.073
H57	0.016	0.081	1.050	0.382	1.053

Table S12: SS-RASSCF(19,26) calculated hyperfine coupling tensors (in MHz) for the ^{13}C and ^{1}H nuclei in [ThCp$^{tt}_3$] (Optimised structure).

Atom	A_1	A_2	A_3	A_{iso}	A_{Euclid}
H58	-1.160	-1.265	2.756	0.110	3.247
H59	-0.554	-0.707	1.320	0.020	1.597
H60	-0.454	-0.487	1.008	0.022	1.209
H61	-0.273	-0.370	1.595	0.317	1.660
H62	-0.810	-0.918	1.760	0.011	2.144
H63	-0.808	-1.369	5.539	1.121	5.762

Table S13: SA-RASSCF(19,27) calculated hyperfine coupling tensors (in MHz) for the ^{13}C and ^{1}H nuclei in [ThCp$^{tt}_3$] (Optimised structure).

Atom	A_1	A_2	A_3	A_{iso}	A_{Euclid}
C1	-0.437	-0.866	-0.910	-0.738	1.329
C2	-1.341	-1.416	2.866	0.036	3.467
C3	-0.435	-0.865	-0.908	-0.736	1.327
C4	0.080	0.639	0.892	0.537	1.101
C5	0.082	0.640	0.892	0.538	1.101
C6	-0.438	-0.868	-0.910	-0.739	1.332
C7	-1.344	-1.419	2.874	0.037	3.476
C8	-0.439	-0.869	-0.912	-0.740	1.334
C9	0.083	-0.640	0.893	0.112	1.102
C10	0.080	-0.640	0.894	0.111	1.102
C11	-0.440	-0.874	-0.914	-0.743	1.339
C12	-1.349	-1.424	2.887	0.038	3.490
C13	-0.438	-0.873	-0.915	-0.742	1.338
C14	0.085	-0.644	0.893	0.111	1.104
C15	0.081	-0.645	0.894	0.110	1.105
C16	1.158	1.318	2.249	1.575	2.852
C17	1.162	1.322	2.254	1.579	2.860
C18	1.167	1.327	2.260	1.585	2.869
C19	1.167	1.326	2.260	1.584	2.868
C20	1.177	1.336	2.272	1.595	2.887
C21	1.176	1.335	2.271	1.594	2.885
C22	-0.045	-0.089	0.368	0.078	0.381
C23	0.236	0.281	0.914	0.477	0.985
C24	1.688	1.950	3.136	2.258	4.060
C25	1.682	1.944	3.129	2.252	4.049
C26	-0.045	-0.089	0.368	0.078	0.382
C27	0.240	0.285	0.920	0.482	0.993
C28	1.663	1.924	3.100	2.229	4.009
C29	0.242	0.287	0.922	0.484	0.996
C30	-0.044	-0.088	0.370	0.079	0.383
C31	-0.044	-0.088	0.370	0.079	0.383
C32	0.240	0.285	0.919	0.481	0.992
C33	1.665	1.925	3.104	2.231	4.014
C34	1.640	1.900	3.068	2.203	3.964
C35	0.251	0.297	0.934	0.494	1.012
C36	-0.044	-0.088	0.371	0.080	0.384

Table S13: SA-RASSCF(19,27) calculated hyperfine coupling tensors (in MHz) for the ^{13}C and ^{1}H nuclei in [ThCp$^{\mathrm{tt}}_3$] (Optimised structure).

Atom	A_1	A_2	A_3	A_{iso}	A_{Euclid}
C37	-0.044	-0.088	0.371	0.080	0.384
C38	0.248	0.294	0.931	0.491	1.007
C39	1.650	1.912	3.084	2.215	3.986
H1	-1.023	-1.373	2.531	0.045	3.056
H2	-0.584	-0.849	3.155	0.574	3.320
H3	-0.584	-0.848	3.154	0.574	3.317
H4	-1.022	-1.374	2.534	0.046	3.059
H5	-0.582	-0.847	3.157	0.576	3.320
H6	-0.584	-0.849	3.155	0.574	3.319
H7	-1.023	-1.378	2.538	0.046	3.063
H8	-0.581	-0.847	3.164	0.579	3.327
H9	-0.586	-0.851	3.158	0.574	3.323
H10	0.040	0.116	0.999	0.385	1.006
H11	-0.357	-0.436	0.796	0.001	0.975
H12	-0.323	-0.434	0.767	0.003	0.939
H13	-0.541	-0.627	1.267	0.033	1.514
H14	-1.037	-1.158	2.630	0.145	3.055
H15	-0.418	-0.451	0.963	0.031	1.142
H16	-0.195	-0.321	1.556	0.347	1.601
H17	-0.456	-1.084	5.446	1.302	5.572
H18	-0.771	-0.820	1.665	0.025	2.010
H19	-0.198	-0.324	1.553	0.344	1.599
H20	-0.769	-0.818	1.663	0.025	2.006
H21	-0.458	-1.086	5.442	1.299	5.568
H22	-0.323	-0.434	0.768	0.004	0.940
H23	-0.357	-0.436	0.796	0.001	0.975
H24	0.040	0.116	0.999	0.385	1.007
H25	-0.418	-0.451	0.964	0.032	1.143
H26	-1.037	-1.159	2.637	0.147	3.061
H27	-0.542	-0.628	1.270	0.033	1.516
H28	-0.201	-0.325	1.547	0.340	1.593
H29	-0.462	-1.085	5.418	1.290	5.544
H30	-0.768	-0.816	1.660	0.025	2.003
H31	-0.542	-0.627	1.270	0.034	1.517
H32	-1.038	-1.160	2.639	0.147	3.064
H33	-0.418	-0.451	0.964	0.032	1.144
H34	0.040	0.116	0.999	0.385	1.007
H35	-0.357	-0.435	0.796	0.001	0.975
H36	-0.324	-0.435	0.768	0.003	0.940
H37	-0.324	-0.435	0.768	0.003	0.940
H38	-0.357	-0.436	0.796	0.001	0.975
H39	0.040	0.116	0.999	0.385	1.006
H40	-0.417	-0.450	0.963	0.032	1.142
H41	-1.035	-1.157	2.632	0.147	3.055
H42	-0.543	-0.628	1.271	0.033	1.518
H43	-0.767	-0.816	1.660	0.026	2.002
H44	-0.462	-1.085	5.422	1.292	5.549
H45	-0.201	-0.326	1.547	0.340	1.594

Table S13: SA-RASSCF(19,27) calculated hyperfine coupling tensors (in MHz) for the ^{13}C and ^{1}H nuclei in [ThCp$^{tt}_3$] (Optimised structure).

Atom	A_1	A_2	A_3	A_{iso}	A_{Euclid}
H46	-0.205	-0.330	1.539	0.335	1.587
H47	-0.466	-1.087	5.392	1.280	5.520
H48	-0.767	-0.813	1.656	0.025	1.997
H49	-0.418	-0.451	0.968	0.033	1.147
H50	-0.543	-0.629	1.274	0.034	1.521
H51	-1.038	-1.164	2.653	0.150	3.077
H52	-0.357	-0.435	0.796	0.001	0.975
H53	-0.324	-0.436	0.769	0.003	0.942
H54	0.040	0.116	1.000	0.385	1.007
H55	-0.324	-0.436	0.769	0.003	0.942
H56	-0.357	-0.436	0.796	0.001	0.975
H57	0.040	0.117	1.001	0.386	1.008
H58	-1.038	-1.162	2.648	0.149	3.073
H59	-0.543	-0.629	1.274	0.034	1.521
H60	-0.418	-0.451	0.967	0.033	1.146
H61	-0.204	-0.329	1.543	0.337	1.590
H62	-0.767	-0.814	1.658	0.026	2.000
H63	-0.462	-1.085	5.407	1.287	5.535

Table S14: SA-RASSCF(27,36) calculated hyperfine coupling tensors (in MHz) for the ^{13}C and ^{1}H nuclei in [ThCp$^{tt}_3$] (Optimised structure).

Atom	A_1	A_2	A_3	A_{iso}	A_{Euclid}
C1	0.470	-0.726	-0.846	-0.367	1.210
C2	-1.127	-1.210	2.558	0.074	3.046
C3	0.471	-0.727	-0.846	-0.367	1.211
C4	-0.126	0.702	-0.908	-0.111	1.155
C5	-0.124	0.702	-0.907	-0.110	1.154
C6	0.471	-0.728	-0.847	-0.368	1.212
C7	-1.129	-1.212	2.564	0.074	3.052
C8	0.471	-0.728	-0.847	-0.368	1.212
C9	-0.123	0.701	-0.907	-0.110	1.153
C10	-0.126	0.703	-0.909	-0.111	1.156
C11	0.472	-0.730	-0.851	-0.370	1.217
C12	-1.132	-1.216	2.575	0.076	3.064
C13	0.473	-0.731	-0.852	-0.370	1.218
C14	-0.122	0.703	-0.908	-0.109	1.154
C15	-0.125	0.706	-0.910	-0.110	1.159
C16	1.046	1.198	2.146	1.463	2.671
C17	1.050	1.202	2.150	1.467	2.678
C18	1.054	1.206	2.156	1.472	2.686
C19	1.054	1.206	2.155	1.472	2.685
C20	1.063	1.215	2.167	1.482	2.702
C21	1.062	1.214	2.166	1.481	2.701
C22	-0.095	-0.135	0.315	0.028	0.355
C23	0.147	0.186	0.821	0.385	0.854
C24	1.381	1.628	2.753	1.921	3.484

Table S14: SA-RASSCF(27,36) calculated hyperfine coupling tensors (in MHz) for the ^{13}C and ^{1}H nuclei in [ThCp$^{tt}_{3}$] (Optimised structure).

Atom	A_1	A_2	A_3	A_{iso}	A_{Euclid}
C25	1.375	1.623	2.747	1.915	3.474
C26	-0.095	-0.135	0.315	0.028	0.355
C27	0.150	0.189	0.826	0.388	0.861
C28	1.358	1.605	2.721	1.895	3.439
C29	0.152	0.191	0.828	0.390	0.863
C30	-0.094	-0.134	0.316	0.029	0.356
C31	-0.094	-0.134	0.316	0.029	0.356
C32	0.150	0.189	0.825	0.388	0.860
C33	1.359	1.606	2.724	1.896	3.442
C34	1.338	1.585	2.693	1.872	3.399
C35	0.158	0.199	0.838	0.398	0.875
C36	-0.094	-0.134	0.317	0.030	0.357
C37	-0.094	-0.134	0.317	0.030	0.357
C38	0.157	0.196	0.835	0.396	0.872
C39	1.347	1.595	2.707	1.883	3.419
H1	-1.083	-1.293	2.485	0.036	3.003
H2	-0.630	-0.912	3.140	0.533	3.330
H3	-0.629	-0.912	3.138	0.532	3.328
H4	-1.082	-1.294	2.487	0.037	3.005
H5	-0.628	-0.911	3.141	0.534	3.330
H6	-0.630	-0.913	3.140	0.532	3.330
H7	-1.083	-1.297	2.490	0.037	3.009
H8	-0.628	-0.911	3.148	0.536	3.337
H9	-0.632	-0.915	3.142	0.532	3.333
H10	0.003	-0.064	0.941	0.293	0.943
H11	-0.376	-0.450	0.825	0.000	1.013
H12	-0.343	-0.442	0.789	0.001	0.967
H13	-0.556	-0.632	1.275	0.029	1.528
H14	-1.089	-1.182	2.632	0.120	3.084
H15	-0.437	-0.464	0.961	0.020	1.153
H16	-0.249	-0.363	1.515	0.301	1.577
H17	-0.616	-1.184	5.381	1.194	5.544
H18	-0.788	-0.834	1.684	0.021	2.038
H19	-0.251	-0.365	1.512	0.299	1.575
H20	-0.786	-0.831	1.682	0.022	2.034
H21	-0.618	-1.186	5.375	1.190	5.539
H22	-0.343	-0.443	0.789	0.001	0.968
H23	-0.376	-0.450	0.825	0.000	1.013
H24	0.003	-0.064	0.941	0.293	0.943
H25	-0.437	-0.464	0.962	0.020	1.154
H26	-1.090	-1.184	2.638	0.121	3.090
H27	-0.557	-0.633	1.277	0.029	1.530
H28	-0.253	-0.366	1.506	0.296	1.571
H29	-0.621	-1.184	5.352	1.182	5.517
H30	-0.785	-0.830	1.679	0.021	2.031
H31	-0.557	-0.633	1.277	0.029	1.531
H32	-1.090	-1.185	2.640	0.122	3.093
H33	-0.437	-0.464	0.962	0.020	1.154

Table S14: SA-RASSCF(27,36) calculated hyperfine coupling tensors (in MHz) for the ^{13}C and ^{1}H nuclei in [ThCp$^{tt}_3$] (Optimised structure).

Atom	A_1	A_2	A_3	A_{iso}	A_{Euclid}
H34	0.003	-0.064	0.941	0.293	0.943
H35	-0.376	-0.450	0.825	0.000	1.013
H36	-0.344	-0.443	0.789	0.001	0.968
H37	-0.344	-0.443	0.789	0.001	0.968
H38	-0.376	-0.450	0.825	0.000	1.013
H39	0.003	-0.064	0.941	0.293	0.943
H40	-0.436	-0.463	0.961	0.021	1.153
H41	-1.087	-1.181	2.633	0.122	3.084
H42	-0.558	-0.634	1.278	0.029	1.532
H43	-0.785	-0.829	1.679	0.022	2.030
H44	-0.621	-1.185	5.355	1.183	5.520
H45	-0.254	-0.367	1.506	0.295	1.571
H46	-0.258	-0.370	1.499	0.290	1.565
H47	-0.625	-1.186	5.326	1.172	5.493
H48	-0.784	-0.826	1.675	0.022	2.025
H49	-0.437	-0.465	0.965	0.021	1.157
H50	-0.558	-0.635	1.282	0.030	1.535
H51	-1.091	-1.189	2.654	0.125	3.106
H52	-0.376	-0.450	0.825	0.000	1.012
H53	-0.345	-0.444	0.790	0.000	0.970
H54	-0.004	-0.064	0.942	0.291	0.944
H55	-0.344	-0.444	0.791	0.001	0.970
H56	-0.376	-0.450	0.825	0.000	1.013
H57	-0.004	-0.065	0.942	0.291	0.945
H58	-1.091	-1.187	2.649	0.124	3.101
H59	-0.558	-0.634	1.281	0.030	1.535
H60	-0.437	-0.465	0.964	0.021	1.156
H61	-0.257	-0.370	1.502	0.292	1.568
H62	-0.784	-0.827	1.677	0.022	2.027
H63	-0.621	-1.184	5.341	1.179	5.506

Table S15: SA-CASSCF(3,7) calculated hyperfine coupling tensors (in MHz) for the ^{1}H nuclei in [UCp$^{tt}_3$] (Optimised structure).

Atom	A_1	A_2	A_3	A_{iso}	A_{Euclid}
H1	-0.032	2.952	-4.602	-0.561	5.468
H2	-0.136	3.539	-5.559	-0.719	6.591
H3	-0.072	3.523	-5.510	-0.686	6.541
H4	-0.042	2.671	-5.081	-0.817	5.741
H5	-0.163	3.762	-5.245	-0.549	6.456
H6	-0.048	3.730	-5.188	-0.502	6.390
H7	-0.048	2.807	-4.824	-0.688	5.582
H8	-0.143	3.777	-5.139	-0.502	6.380
H9	-0.069	3.812	-5.036	-0.431	6.316
H10	-0.017	0.520	-0.865	-0.121	1.010
H11	-0.018	0.713	-1.457	-0.254	1.622
H12	-0.016	0.668	-1.346	-0.231	1.502

Table S15: SA-CASSCF(3,7) calculated hyperfine coupling tensors (in MHz) for the ^{1}H nuclei in [UCp$^{tt}_{3}$] (Optimised structure).

Atom	A_1	A_2	A_3	A_{iso}	A_{Euclid}
H13	-0.018	1.027	-1.655	-0.215	1.948
H14	-0.032	1.828	-2.427	-0.210	3.039
H15	-0.016	0.700	-1.014	-0.110	1.232
H16	0.033	0.734	-1.061	-0.098	1.291
H17	0.083	2.030	2.542	1.552	3.254
H18	-0.040	1.305	-2.155	-0.296	2.520
H19	-0.023	0.664	-0.903	-0.087	1.122
H20	0.039	1.126	-1.786	-0.207	2.112
H21	0.077	1.753	2.071	1.301	2.715
H22	-0.013	0.673	-1.278	-0.206	1.445
H23	-0.018	0.697	-1.361	-0.227	1.530
H24	-0.012	0.499	-0.787	-0.100	0.932
H25	-0.017	0.650	-0.924	-0.097	1.130
H26	-0.042	1.539	-2.120	-0.208	2.621
H27	-0.023	1.054	-1.663	-0.211	1.969
H28	-0.033	-0.719	-1.086	-0.613	1.303
H29	-0.087	-1.999	2.629	0.181	3.304
H30	-0.040	1.353	-2.050	-0.246	2.457
H31	-0.018	0.928	-1.817	-0.302	2.041
H32	-0.032	1.667	-2.669	-0.345	3.147
H33	-0.016	0.637	-1.111	-0.163	1.281
H34	-0.018	0.499	-0.904	-0.141	1.032
H35	-0.018	0.712	-1.452	-0.253	1.617
H36	-0.017	0.636	-1.406	-0.262	1.543
H37	-0.014	0.631	-1.343	-0.242	1.484
H38	-0.018	0.697	-1.375	-0.232	1.542
H39	-0.016	0.477	-0.829	-0.123	0.956
H40	-0.017	0.587	-1.022	-0.151	1.179
H41	-0.039	1.409	-2.317	-0.316	2.712
H42	-0.023	0.927	-1.799	-0.298	2.024
H43	0.040	1.203	-1.760	-0.172	2.132
H44	0.083	1.763	2.260	1.369	2.868
H45	-0.024	0.653	-0.958	-0.109	1.160
H46	0.034	0.797	-0.988	-0.052	1.270
H47	0.087	2.218	2.406	1.570	3.273
H48	-0.041	1.441	-1.948	-0.183	2.424
H49	-0.016	0.675	-1.051	-0.131	1.249
H50	-0.019	0.979	-1.723	-0.254	1.982
H51	-0.033	1.670	-2.660	-0.341	3.141
H52	-0.018	0.778	-1.329	-0.190	1.540
H53	-0.017	0.699	-1.270	-0.196	1.450
H54	-0.017	0.547	-0.817	-0.096	0.983
H55	-0.015	0.697	-1.226	-0.181	1.411
H56	-0.017	0.755	-1.246	-0.169	1.458
H57	-0.016	0.519	-0.746	-0.081	0.909
H58	-0.041	1.382	-2.322	-0.327	2.703
H59	-0.024	0.971	-1.745	-0.266	1.997
H60	-0.018	0.609	-0.974	-0.127	1.148

Table S15: SA-CASSCF(3,7) calculated hyperfine coupling tensors (in MHz) for the ^{1}H nuclei in [UCp$^{tt}_3$] (Optimised structure).

Atom	A_1	A_2	A_3	A_{iso}	A_{Euclid}
H61	0.022	0.696	-0.851	-0.044	1.100
H62	0.038	1.252	-1.630	-0.114	2.056
H63	0.076	1.790	1.964	1.277	2.659

Table S16: SA-RASSCF(21,30) calculated hyperfine coupling tensors (in MHz) for the ^{1}H nuclei in [UCp$^{tt}_3$] (Optimised structure).

Atom	A_1	A_2	A_3	A_{iso}	A_{Euclid}
H1	0.318	2.984	-4.947	-0.548	5.786
H2	0.522	3.784	-5.597	-0.430	6.777
H3	0.368	3.793	-5.661	-0.500	6.824
H4	0.319	2.843	-5.189	-0.676	5.925
H5	0.516	3.804	-5.528	-0.403	6.730
H6	0.368	3.809	-5.574	-0.466	6.761
H7	0.317	2.842	-5.161	-0.667	5.900
H8	0.515	3.932	-5.312	-0.288	6.629
H9	0.364	3.974	-5.349	-0.337	6.674
H10	0.090	0.579	-0.912	-0.081	1.084
H11	0.105	0.780	-1.504	-0.206	1.697
H12	0.090	0.718	-1.424	-0.205	1.597
H13	0.122	1.055	-1.779	-0.201	2.072
H14	0.235	1.847	-2.621	-0.180	3.215
H15	0.100	0.725	-1.082	-0.086	1.306
H16	0.155	0.811	-1.095	-0.043	1.372
H17	-0.472	2.220	2.605	1.451	3.455
H18	0.236	1.421	-2.171	-0.171	2.606
H19	0.135	0.739	-0.948	-0.025	1.210
H20	-0.217	1.240	-1.824	-0.267	2.216
H21	-0.412	1.934	2.157	1.226	2.926
H22	0.087	0.721	-1.366	-0.186	1.547
H23	0.103	0.762	-1.426	-0.187	1.621
H24	0.078	0.557	-0.836	-0.067	1.008
H25	0.096	0.681	-0.995	-0.073	1.210
H26	0.223	1.583	-2.283	-0.159	2.787
H27	0.132	1.088	-1.790	-0.190	2.099
H28	0.157	-0.783	-1.133	-0.586	1.386
H29	-0.480	2.155	2.723	1.466	3.506
H30	0.235	1.409	-2.158	-0.171	2.588
H31	0.122	1.002	-1.859	-0.245	2.115
H32	0.237	1.799	-2.696	-0.220	3.250
H33	0.101	0.690	-1.133	-0.114	1.331
H34	0.090	0.553	-0.948	-0.102	1.102
H35	0.105	0.758	-1.538	-0.225	1.718
H36	0.091	0.683	-1.485	-0.237	1.637
H37	0.086	0.682	-1.423	-0.218	1.581
H38	0.104	0.745	-1.473	-0.208	1.654
H39	0.080	0.532	-0.877	-0.088	1.029

Table S16: SA-RASSCF(21,30) calculated hyperfine coupling tensors (in MHz) for the ^{1}H nuclei in [UCp$^{tt}_3$] (Optimised structure).

Atom	A_1	A_2	A_3	A_{iso}	A_{Euclid}
H40	0.095	0.649	-1.045	-0.100	1.234
H41	0.217	1.547	-2.341	-0.192	2.814
H42	0.127	1.013	-1.833	-0.231	2.098
H43	0.226	1.270	-1.875	-0.126	2.276
H44	-0.430	1.942	-2.354	-0.281	3.082
H45	0.140	0.727	-1.009	-0.047	1.252
H46	-0.158	0.825	-1.089	-0.141	1.375
H47	-0.483	2.265	2.634	1.472	3.508
H48	0.237	1.488	-2.069	-0.115	2.559
H49	0.100	0.695	-1.129	-0.111	1.329
H50	0.122	1.006	-1.853	-0.242	2.112
H51	0.235	1.753	-2.765	-0.259	3.282
H52	0.105	0.799	-1.462	-0.186	1.670
H53	0.090	0.711	-1.421	-0.207	1.591
H54	0.089	0.578	-0.909	-0.081	1.081
H55	0.087	0.713	-1.378	-0.193	1.554
H56	0.102	0.776	-1.394	-0.172	1.599
H57	0.079	0.552	-0.836	-0.068	1.005
H58	0.219	1.492	-2.388	-0.226	2.824
H59	0.129	1.018	-1.854	-0.236	2.119
H60	0.095	0.645	-1.040	-0.100	1.228
H61	-0.134	0.734	-0.949	-0.116	1.207
H62	0.219	1.305	-1.764	-0.080	2.205
H63	-0.402	1.880	2.152	1.210	2.885

Table S17: SA-RASCI(29,34) calculated hyperfine coupling tensors (in MHz) for the ^{1}H nuclei in [UCp$^{tt}_3$] (Optimised structure).

Atom	A_1	A_2	A_3	A_{iso}	A_{Euclid}
H1	0.297	2.953	-4.935	-0.562	5.758
H2	0.492	3.786	-5.509	-0.410	6.702
H3	0.337	3.801	-5.575	-0.479	6.756
H4	0.298	2.844	-5.121	-0.660	5.865
H5	0.486	3.768	-5.491	-0.412	6.678
H6	0.337	3.779	-5.546	-0.477	6.719
H7	0.297	2.816	-5.139	-0.675	5.868
H8	0.485	3.899	-5.275	-0.297	6.577
H9	0.333	3.945	-5.320	-0.347	6.631
H10	0.086	0.577	-0.903	-0.080	1.075
H11	0.100	0.779	-1.485	-0.202	1.680
H12	0.086	0.713	-1.408	-0.203	1.581
H13	0.115	1.045	-1.772	-0.204	2.061
H14	0.222	1.830	-2.621	-0.190	3.204
H15	0.095	0.720	-1.079	-0.088	1.300
H16	0.147	0.811	-1.085	-0.042	1.362
H17	-0.450	2.224	2.585	1.453	3.440
H18	0.224	1.427	-2.146	-0.165	2.587

Table S17: SA-RASCI(29,34) calculated hyperfine coupling tensors (in MHz) for the ^{1}H nuclei in [UCp^{tt}_{3}] (Optimised structure).

Atom	A_1	A_2	A_3	A_{iso}	A_{Euclid}
H19	0.126	0.733	-0.937	-0.026	1.196
H20	-0.203	1.238	-1.799	-0.255	2.193
H21	-0.386	1.923	2.134	1.224	2.899
H22	0.081	0.714	-1.352	-0.186	1.531
H23	0.096	0.757	-1.407	-0.185	1.601
H24	0.074	0.552	-0.825	-0.066	0.996
H25	0.090	0.671	-0.991	-0.077	1.201
H26	0.209	1.562	-2.280	-0.170	2.772
H27	0.124	1.074	-1.785	-0.196	2.086
H28	0.149	-0.782	-1.124	-0.586	1.377
H29	-0.457	2.158	2.705	1.469	3.491
H30	0.223	1.403	-2.152	-0.175	2.578
H31	0.116	1.003	-1.833	-0.238	2.093
H32	0.223	1.804	-2.664	-0.212	3.225
H33	0.096	0.692	-1.119	-0.110	1.319
H34	0.086	0.552	-0.939	-0.100	1.092
H35	0.100	0.753	-1.525	-0.224	1.704
H36	0.086	0.681	-1.466	-0.233	1.619
H37	0.081	0.678	-1.404	-0.215	1.561
H38	0.097	0.737	-1.457	-0.208	1.636
H39	0.075	0.528	-0.865	-0.087	1.016
H40	0.089	0.647	-1.029	-0.098	1.219
H41	0.204	1.544	-2.311	-0.188	2.787
H42	0.120	1.011	-1.806	-0.225	2.073
H43	-0.212	1.258	-1.861	-0.272	2.256
H44	-0.403	1.935	-2.325	-0.264	3.051
H45	0.131	0.722	-0.994	-0.047	1.236
H46	-0.150	0.815	-1.090	-0.142	1.369
H47	-0.460	2.246	-2.641	-0.285	3.498
H48	0.226	1.477	-2.069	-0.122	2.552
H49	0.095	0.691	-1.124	-0.113	1.322
H50	0.115	0.998	-1.843	-0.243	2.099
H51	0.222	1.751	-2.742	-0.256	3.261
H52	0.100	0.788	-1.462	-0.191	1.663
H53	0.086	0.701	-1.418	-0.210	1.584
H54	0.085	0.570	-0.909	-0.085	1.076
H55	0.081	0.701	-1.375	-0.198	1.545
H56	0.096	0.762	-1.391	-0.178	1.589
H57	0.074	0.542	-0.833	-0.072	0.997
H58	0.205	1.485	-2.364	-0.225	2.800
H59	0.122	1.009	-1.841	-0.237	2.103
H60	0.089	0.638	-1.033	-0.102	1.217
H61	-0.125	0.720	-0.946	-0.117	1.195
H62	0.205	1.287	-1.760	-0.089	2.190
H63	-0.377	1.855	2.146	1.208	2.861

Table S18: SA-RASCI(29,35) calculated hyperfine coupling tensors (in MHz) for the ^{1}H nuclei in [UCp_3^{tt}] (Optimised structure).

Atom	A_1	A_2	A_3	A_{iso}	A_{Euclid}
H1	0.284	2.947	-4.914	-0.561	5.737
H2	0.475	3.776	-5.498	-0.416	6.686
H3	0.317	3.784	-5.550	-0.483	6.724
H4	0.286	2.835	-5.104	-0.661	5.846
H5	0.471	3.762	-5.474	-0.414	6.659
H6	0.317	3.765	-5.516	-0.478	6.686
H7	0.284	2.811	-5.117	-0.674	5.846
H8	0.469	3.891	-5.261	-0.300	6.560
H9	0.313	3.930	-5.292	-0.350	6.599
H10	0.082	0.573	-0.900	-0.082	1.070
H11	0.095	0.774	-1.481	-0.204	1.674
H12	0.083	0.709	-1.407	-0.205	1.578
H13	0.110	1.040	-1.766	-0.205	2.053
H14	0.212	1.825	-2.610	-0.191	3.192
H15	0.090	0.718	-1.075	-0.089	1.296
H16	0.141	0.808	-1.081	-0.044	1.357
H17	-0.431	2.216	2.584	1.456	3.432
H18	0.214	1.421	-2.142	-0.169	2.579
H19	0.121	0.730	-0.935	-0.028	1.192
H20	-0.195	1.232	-1.794	-0.252	2.185
H21	-0.368	1.920	2.129	1.227	2.890
H22	0.077	0.712	-1.347	-0.186	1.526
H23	0.092	0.752	-1.403	-0.186	1.595
H24	0.070	0.547	-0.822	-0.068	0.990
H25	0.086	0.669	-0.987	-0.077	1.196
H26	0.200	1.557	-2.271	-0.171	2.761
H27	0.118	1.070	-1.777	-0.196	2.077
H28	0.143	-0.780	-1.121	-0.586	1.372
H29	-0.438	2.152	2.703	1.472	3.483
H30	0.214	1.399	-2.146	-0.178	2.570
H31	0.111	0.998	-1.828	-0.240	2.085
H32	0.213	1.796	-2.657	-0.216	3.214
H33	0.091	0.690	-1.116	-0.112	1.315
H34	0.082	0.548	-0.936	-0.102	1.088
H35	0.096	0.750	-1.520	-0.225	1.698
H36	0.084	0.677	-1.464	-0.234	1.616
H37	0.077	0.675	-1.400	-0.216	1.556
H38	0.093	0.733	-1.452	-0.209	1.630
H39	0.072	0.523	-0.862	-0.089	1.011
H40	0.085	0.644	-1.026	-0.099	1.214
H41	0.195	1.536	-2.305	-0.191	2.777
H42	0.115	1.006	-1.801	-0.227	2.066
H43	-0.203	1.254	-1.855	-0.268	2.248
H44	-0.385	1.932	-2.319	-0.257	3.042
H45	0.126	0.719	-0.992	-0.049	1.232
H46	-0.143	0.814	-1.085	-0.138	1.364
H47	-0.441	2.242	-2.636	-0.278	3.488
H48	0.216	1.472	-2.063	-0.125	2.544

Table S18: SA-RASCI(29,35) calculated hyperfine coupling tensors (in MHz) for the ^{1}H nuclei in [UCp$^{tt}_3$] (Optimised structure).

Atom	A_1	A_2	A_3	A_{iso}	A_{Euclid}
H49	0.091	0.689	-1.120	-0.113	1.318
H50	0.110	0.994	-1.836	-0.244	2.090
H51	0.211	1.744	-2.733	-0.259	3.249
H52	0.096	0.785	-1.456	-0.192	1.657
H53	0.083	0.698	-1.414	-0.211	1.580
H54	0.082	0.567	-0.905	-0.085	1.071
H55	0.077	0.699	-1.369	-0.198	1.539
H56	0.092	0.758	-1.386	-0.179	1.583
H57	0.071	0.538	-0.829	-0.073	0.991
H58	0.197	1.479	-2.357	-0.227	2.789
H59	0.116	1.005	-1.834	-0.238	2.094
H60	0.085	0.636	-1.029	-0.103	1.212
H61	-0.120	0.718	-0.943	-0.115	1.191
H62	0.196	1.282	-1.754	-0.092	2.181
H63	-0.360	1.854	2.138	1.211	2.853

Table S19: SA-RASSCF(19,27)-SO calculated hyperfine coupling tensors (in MHz) for the ^{1}H and ^{13}C nuclei in [ThCp$^{tt}_3$] (Optimised structure) with a Gaussian nucleus model.

Atom	A_1	A_2	A_3	A_{iso}	A_{Euclid}
C1	-0.437	-0.866	-0.910	-0.737	1.329
C2	-1.341	-1.416	2.866	0.036	3.467
C3	-0.435	-0.865	-0.908	-0.736	1.327
C4	0.080	0.639	0.892	0.537	1.101
C5	0.082	0.640	0.892	0.538	1.101
C6	-0.438	-0.868	-0.910	-0.739	1.332
C7	-1.344	-1.419	2.874	0.037	3.476
C8	-0.439	-0.869	-0.912	-0.740	1.334
C9	0.083	-0.640	0.893	0.112	1.102
C10	0.080	-0.640	0.894	0.111	1.102
C11	-0.440	-0.874	-0.914	-0.743	1.339
C12	-1.349	-1.424	2.887	0.038	3.490
C13	-0.438	-0.873	-0.915	-0.742	1.338
C14	0.085	-0.644	0.893	0.111	1.104
C15	0.081	-0.645	0.894	0.110	1.105
C16	1.158	1.318	2.249	1.575	2.852
C17	1.162	1.322	2.254	1.579	2.860
C18	1.167	1.327	2.260	1.585	2.869
C19	1.167	1.326	2.260	1.584	2.868
C20	1.177	1.336	2.272	1.595	2.887
C21	1.176	1.335	2.271	1.594	2.885
C22	-0.045	-0.089	0.368	0.078	0.381
C23	0.236	0.281	0.914	0.477	0.985
C24	1.688	1.950	3.136	2.258	4.060
C25	1.682	1.944	3.129	2.252	4.049
C26	-0.045	-0.089	0.368	0.078	0.382
C27	0.240	0.285	0.920	0.482	0.993

Table S19: SA-RASSCF(19,27)-SO calculated hyperfine coupling tensors (in MHz) for the ^{1}H and ^{13}C nuclei in [ThCp$^{tt}_3$] (Optimised structure) with a Gaussian nucleus model.

Atom	A_1	A_2	A_3	A_{iso}	A_{Euclid}
C28	1.663	1.924	3.100	2.229	4.009
C29	0.242	0.287	0.922	0.484	0.996
C30	-0.044	-0.088	0.370	0.079	0.383
C31	-0.044	-0.088	0.370	0.080	0.383
C32	0.240	0.285	0.919	0.482	0.992
C33	1.664	1.925	3.104	2.231	4.014
C34	1.640	1.900	3.068	2.203	3.964
C35	0.251	0.297	0.934	0.494	1.012
C36	-0.044	-0.088	0.371	0.080	0.384
C37	-0.044	-0.088	0.371	0.080	0.384
C38	0.248	0.294	0.931	0.491	1.007
C39	1.650	1.912	3.084	2.215	3.986
H1	-1.023	-1.373	2.531	0.0453	3.056
H2	-0.584	-0.849	3.155	0.5740	3.320
H3	-0.584	-0.848	3.154	0.5740	3.317
H4	-1.022	-1.374	2.534	0.0457	3.059
H5	-0.582	-0.847	3.157	0.5760	3.320
H6	-0.584	-0.849	3.155	0.5737	3.319
H7	-1.023	-1.378	2.538	0.0457	3.063
H8	-0.581	-0.847	3.164	0.5787	3.327
H9	-0.586	-0.851	3.158	0.5737	3.323
H10	0.040	0.116	0.999	0.3847	1.006
H11	-0.357	-0.436	0.796	0.0010	0.975
H12	-0.323	-0.434	0.767	0.0037	0.939
H13	-0.541	-0.627	1.267	0.0333	1.514
H14	-1.037	-1.158	2.630	0.1450	3.055
H15	-0.418	-0.451	0.963	0.0313	1.142
H16	-0.195	-0.321	1.556	0.3467	1.601
H17	-0.456	-1.084	5.446	1.3020	5.572
H18	-0.771	-0.820	1.665	0.0247	2.010
H19	-0.198	-0.324	1.553	0.3437	1.599
H20	-0.769	-0.818	1.663	0.0253	2.006
H21	-0.458	-1.086	5.442	1.2990	5.568
H22	-0.323	-0.434	0.768	0.0037	0.940
H23	-0.357	-0.436	0.796	0.0013	0.975
H24	0.040	0.116	0.999	0.3850	1.007
H25	-0.418	-0.451	0.964	0.0317	1.143
H26	-1.037	-1.159	2.637	0.1467	3.061
H27	-0.542	-0.628	1.270	0.0333	1.516
H28	-0.201	-0.325	1.547	0.3403	1.593
H29	-0.462	-1.085	5.418	1.2903	5.544
H30	-0.768	-0.816	1.660	0.0250	2.003
H31	-0.542	-0.627	1.270	0.0337	1.517
H32	-1.038	-1.160	2.639	0.1470	3.064
H33	-0.418	-0.451	0.964	0.0317	1.144
H34	0.040	0.116	0.999	0.3850	1.007
H35	-0.357	-0.435	0.796	0.0013	0.975
H36	-0.324	-0.435	0.768	0.0033	0.940

Table S19: SA-RASSCF(19,27)-SO calculated hyperfine coupling tensors (in MHz) for the ^{1}H and ^{13}C nuclei in [ThCp$_3^{tt}$] (Optimised structure) with a Gaussian nucleus model.

Atom	A_1	A_2	A_3	A_{iso}	A_{Euclid}
H37	-0.324	-0.435	0.768	0.0033	0.940
H38	-0.357	-0.436	0.796	0.0013	0.975
H39	0.040	0.116	0.999	0.3847	1.006
H40	-0.417	-0.450	0.963	0.0320	1.142
H41	-1.035	-1.157	2.632	0.1467	3.055
H42	-0.543	-0.628	1.271	0.0333	1.518
H43	-0.767	-0.816	1.660	0.0257	2.002
H44	-0.462	-1.085	5.422	1.2917	5.549
H45	-0.201	-0.326	1.547	0.3397	1.594
H46	-0.205	-0.330	1.539	0.3347	1.587
H47	-0.466	-1.087	5.392	1.2793	5.520
H48	-0.767	-0.813	1.656	0.0253	1.997
H49	-0.418	-0.451	0.968	0.0330	1.147
H50	-0.543	-0.629	1.274	0.0340	1.521
H51	-1.038	-1.164	2.653	0.1503	3.077
H52	-0.357	-0.435	0.796	0.0013	0.975
H53	-0.324	-0.436	0.769	0.0030	0.942
H54	0.040	0.116	1.000	0.3853	1.007
H55	-0.324	-0.436	0.769	0.0030	0.942
H56	-0.357	-0.436	0.796	0.0013	0.975
H57	0.040	0.117	1.001	0.3860	1.008
H58	-1.038	-1.162	2.648	0.1493	3.073
H59	-0.543	-0.629	1.274	0.0340	1.521
H60	-0.418	-0.451	0.967	0.0327	1.146
H61	-0.204	-0.329	1.543	0.3363	1.590
H62	-0.767	-0.814	1.658	0.0257	2.000
H63	-0.462	-1.085	5.407	1.2870	5.535

Table S20: SA-RASSCF(21,30)-SO calculated hyperfine coupling tensors (in MHz) for the ^{1}H nuclei in [UCp$_3^{tt}$] (Optimised structure) using the Gaussian nucleus model.

Atom	A_1	A_2	A_3	A_{iso}	A_{Euclid}
H1	0.318	2.984	-4.947	-0.548	5.786
H2	0.522	3.784	-5.597	-0.430	6.777
H3	0.368	3.793	-5.661	-0.500	6.824
H4	0.319	2.843	-5.189	-0.676	5.925
H5	0.516	3.804	-5.528	-0.403	6.730
H6	0.368	3.809	-5.574	-0.466	6.761
H7	0.317	2.842	-5.161	-0.667	5.900
H8	0.515	3.932	-5.312	-0.288	6.629
H9	0.364	3.974	-5.349	-0.337	6.674
H10	0.090	0.579	-0.912	-0.081	1.084
H11	0.105	0.780	-1.504	-0.206	1.697
H12	0.090	0.718	-1.424	-0.205	1.597
H13	0.122	1.055	-1.779	-0.201	2.072
H14	0.235	1.847	-2.621	-0.179	3.215
H15	0.100	0.725	-1.082	-0.086	1.306

Table S20: SA-RASSCF(21,30)-SO calculated hyperfine coupling tensors (in MHz) for the ^{1}H nuclei in [UCp$^{tt}_{3}$] (Optimised structure) using the Gaussian nucleus model.

Atom	A_1	A_2	A_3	A_{iso}	A_{Euclid}
H16	0.155	0.811	-1.095	-0.043	1.372
H17	-0.472	2.220	2.605	1.451	3.455
H18	0.236	1.421	-2.171	-0.171	2.606
H19	0.135	0.739	-0.948	-0.025	1.210
H20	-0.217	1.240	-1.824	-0.267	2.216
H21	-0.412	1.934	2.157	1.226	2.926
H22	0.087	0.721	-1.366	-0.186	1.547
H23	0.103	0.762	-1.426	-0.187	1.621
H24	0.078	0.557	-0.836	-0.067	1.008
H25	0.096	0.681	-0.995	-0.073	1.210
H26	0.223	1.583	-2.283	-0.159	2.787
H27	0.132	1.088	-1.790	-0.190	2.099
H28	0.157	-0.783	-1.133	-0.587	1.386
H29	-0.480	2.155	2.723	1.466	3.506
H30	0.235	1.409	-2.158	-0.171	2.588
H31	0.122	1.002	-1.859	-0.245	2.115
H32	0.237	1.799	-2.696	-0.220	3.250
H33	0.101	0.690	-1.133	-0.114	1.331
H34	0.090	0.553	-0.948	-0.102	1.102
H35	0.105	0.758	-1.538	-0.225	1.718
H36	0.091	0.683	-1.485	-0.237	1.637
H37	0.086	0.682	-1.423	-0.218	1.581
H38	0.104	0.745	-1.473	-0.208	1.654
H39	0.080	0.532	-0.877	-0.089	1.029
H40	0.095	0.649	-1.045	-0.101	1.234
H41	0.217	1.547	-2.341	-0.192	2.814
H42	0.127	1.013	-1.833	-0.231	2.098
H43	0.226	1.270	-1.875	-0.126	2.276
H44	-0.430	1.942	-2.354	-0.281	3.082
H45	0.140	0.727	-1.009	-0.047	1.252
H46	-0.158	0.825	-1.089	-0.140	1.375
H47	-0.483	2.265	2.634	1.472	3.508
H48	0.237	1.488	-2.069	-0.114	2.559
H49	0.100	0.695	-1.129	-0.111	1.329
H50	0.122	1.006	-1.853	-0.242	2.112
H51	0.235	1.753	-2.765	-0.259	3.282
H52	0.105	0.799	-1.462	-0.186	1.670
H53	0.090	0.711	-1.421	-0.206	1.591
H54	0.089	0.578	-0.909	-0.080	1.081
H55	0.087	0.713	-1.378	-0.193	1.554
H56	0.102	0.776	-1.394	-0.172	1.599
H57	0.079	0.552	-0.836	-0.068	1.005
H58	0.219	1.492	-2.388	-0.226	2.824
H59	0.129	1.018	-1.854	-0.235	2.119
H60	0.095	0.645	-1.040	-0.100	1.228
H61	-0.134	0.734	-0.949	-0.116	1.207
H62	0.219	1.305	-1.764	-0.080	2.205

Table S20: SA-RASSCF(21,30)-SO calculated hyperfine coupling tensors (in MHz) for the ^{1}H nuclei in [UCp^{tt}_3] (Optimised structure) using the Gaussian nucleus model.

Atom	A_1	A_2	A_3	A_{iso}	A_{Euclid}
H63	-0.402	1.880	2.152	1.210	2.885

Table S21: DFT calculated hyperfine coupling tensors (in MHz) for the ^{13}C and ^{1}H nuclei in [$ThCp^{tt}_3$] (XRD structure)

Atom	A_1	A_2	A_3	A_{iso}	A_{Euclid}
C1	-0.435	-1.867	-1.993	-1.432	2.766.
C2	-0.543	-0.712	5.092	1.279	5.171.
C3	-0.480	-1.810	-1.993	-1.428	2.735.
C4	-0.428	0.937	-1.120	-0.204	1.522.
C5	-0.437	0.898	-1.123	-0.221	1.503.
C6	3.717	3.883	5.054	4.218	7.378.
C7	-0.156	-0.755	-0.813	-0.575	1.120.
C8	1.984	2.085	3.007	2.359	4.163.
C9	1.985	2.286	3.462	2.578	4.599.
C10	3.324	3.472	4.656	3.817	6.692.
C11	3.076	3.301	4.557	3.644	6.412.
C12	-0.089	-0.699	-0.754	-0.514	1.032.
C13	0.750	0.805	1.454	1.003	1.823.
C14	-0.615	-1.761	-1.963	-1.446	2.707.
C15	-0.415	-0.580	5.647	1.551	5.691.
C16	-0.378	-1.919	-2.070	-1.456	2.848.
C17	-0.414	-1.020	1.160	-0.091	1.599.
C18	-0.460	0.659	-1.227	-0.343	1.467.
C19	2.888	3.052	4.147	3.362	5.903.
C20	5.135	5.346	7.344	5.942	10.435.
C21	0.674	0.718	1.336	0.909	1.660.
C22	-0.263	-0.796	-0.847	-0.635	1.192.
C23	3.730	3.931	5.227	4.296	7.529.
C24	-0.047	-0.709	-0.750	-0.502	1.033.
C25	0.370	0.402	0.977	0.583	1.120.
C26	7.007	7.191	9.161	7.786	13.592.
C27	-0.281	-1.790	-1.914	-1.328	2.635.
C28	-0.469	-0.614	5.084	1.334	5.142.
C29	-0.724	-1.717	-1.940	-1.461	2.690.
C30	-0.437	0.838	-1.146	-0.248	1.485.
C31	-0.465	0.979	-1.091	-0.192	1.537.
C32	3.489	3.655	4.818	3.987	6.981.
C33	3.985	4.240	5.822	4.682	8.231.
C34	1.226	1.288	2.052	1.522	2.716.
C35	-0.101	-0.727	-0.777	-0.535	1.068.
C36	3.092	3.235	4.344	3.557	6.236.
C37	-0.196	-0.741	-0.795	-0.577	1.104.
C38	0.811	0.872	1.553	1.079	1.957.
C39	2.983	3.223	4.499	3.568	6.287.
H1	1.887	-2.259	-3.072	-1.148	4.254.

Table S21: DFT calculated hyperfine coupling tensors (in MHz) for the ^{13}C and ^{1}H nuclei in [ThCp$^{tt}_3$] (XRD structure)

Atom	A_1	A_2	A_3	A_{iso}	A_{Euclid}
H2	-0.321	0.408	4.051	1.379	4.084.
H3	-0.287	0.421	4.097	1.410	4.128.
H4	-0.091	0.161	1.073	0.381	1.089.
H5	-0.430	-0.511	0.831	-0.037	1.066.
H6	-0.418	-0.658	0.781	-0.098	1.104.
H7	-0.678	-0.935	1.515	-0.033	1.904.
H8	-1.174	-1.606	3.249	0.156	3.809.
H9	-0.420	-0.515	1.323	0.129	1.480.
H10	-0.663	-0.907	1.270	-0.100	1.695.
H11	-1.230	-1.955	4.521	0.445	5.077.
H12	-0.782	-0.931	1.515	-0.066	1.942.
H13	-0.253	-0.502	1.662	0.302	1.754.
H14	-0.912	-1.095	1.754	-0.084	2.260.
H15	-0.943	-1.728	4.803	0.711	5.191.
H16	-0.388	-0.626	0.742	-0.091	1.045.
H17	-0.443	-0.514	0.841	-0.039	1.080.
H18	-0.077	0.168	1.061	0.384	1.077.
H19	-0.475	-0.521	1.006	0.003	1.228.
H20	-1.076	-1.210	2.305	0.006	2.816.
H21	-0.573	-0.760	1.266	-0.022	1.584.
H22	1.853	-2.334	-3.281	-1.254	4.432.
H23	0.142	-0.571	3.926	1.166	3.969.
H24	-0.065	0.611	4.202	1.583	4.247.
H25	-0.392	-0.741	1.847	0.238	2.028.
H26	-1.129	-2.126	6.404	1.049	6.841.
H27	-1.013	-1.312	1.719	-0.202	2.389.
H28	-0.522	-0.711	1.190	-0.014	1.481.
H29	-1.060	-1.160	2.230	0.003	2.728.
H30	-0.501	-0.542	0.994	-0.016	1.238.
H31	0.006	0.227	1.139	0.457	1.161.
H32	-0.425	-0.508	0.881	-0.018	1.102.
H33	-0.354	-0.571	0.770	-0.052	1.022.
H34	-0.347	-0.580	0.786	-0.047	1.036.
H35	-0.461	-0.540	0.952	-0.016	1.188.
H36	0.161	0.432	1.371	0.655	1.446.
H37	-0.486	-0.518	0.955	-0.016	1.190.
H38	-0.975	-1.066	2.073	0.011	2.526.
H39	-0.517	-0.702	1.180	-0.013	1.467.
H40	-1.030	-1.406	2.139	-0.099	2.759.
H41	-0.795	-1.991	6.537	1.250	6.879.
H42	-0.124	0.210	2.499	0.862	2.511.
H43	1.816	-2.213	-3.033	-1.143	4.170.
H44	-0.183	0.491	4.167	1.492	4.199.
H45	0.214	-0.499	3.927	1.214	3.965.
H46	-0.475	-0.771	1.637	0.130	1.870.
H47	-1.074	-2.008	5.521	0.813	5.972.
H48	-0.939	-1.173	1.679	-0.144	2.253.
H49	-0.525	-0.578	1.111	0.003	1.358.

Table S21: DFT calculated hyperfine coupling tensors (in MHz) for the ^{13}C and ^{1}H nuclei in [ThCp$^{tt}_3$] (XRD structure)

Atom	A_1	A_2	A_3	A_{iso}	A_{Euclid}
H50	-0.587	-0.788	1.337	-0.013	1.659.
H51	-1.188	-1.414	2.694	0.031	3.266.
H52	-0.434	-0.516	0.865	-0.028	1.097.
H53	-0.387	-0.625	0.763	-0.083	1.059.
H54	0.013	0.274	1.186	0.491	1.217.
H55	-0.385	-0.610	0.749	-0.082	1.040.
H56	-0.428	-0.498	0.830	-0.032	1.058.
H57	0.102	-0.109	1.002	0.331	1.013.
H58	-1.118	-1.260	2.425	0.016	2.953.
H59	-0.568	-0.770	1.284	-0.018	1.601.
H60	-0.465	-0.521	1.048	0.021	1.259.
H61	-0.240	-0.498	1.703	0.322	1.790.
H62	-0.918	-1.129	1.782	-0.088	2.300.
H63	-0.967	-1.729	4.998	0.768	5.376.

Table S22: DFT calculated hyperfine coupling tensors (in MHz) for the ^{13}C and ^{1}H nuclei in [ThCp$^{tt}_3$] (optimised structure)

Atom	A_1	A_2	A_3	A_{iso}	A_{Euclid}
C1	-0.023	-1.729	-1.881	-1.211	2.554
C2	-0.017	-0.133	6.175	2.008	6.177
C3	-0.020	-1.728	-1.879	-1.209	2.553
C4	-0.639	0.713	-1.399	-0.442	1.695
C5	-0.638	0.714	-1.400	-0.441	1.696
C6	-0.020	-1.727	-1.879	-1.209	2.552
C7	-0.025	-0.143	6.148	1.994	6.149
C8	-0.022	-1.727	-1.878	-1.209	2.552
C9	-0.650	0.695	-1.412	-0.456	1.702
C10	-0.651	0.698	-1.410	-0.454	1.703
C11	-0.025	-1.752	-1.905	-1.227	2.588
C12	-0.022	-0.141	6.227	2.021	6.229
C13	-0.023	-1.751	-1.902	-1.225	2.585
C14	-0.644	0.708	-1.411	-0.449	1.705
C15	-0.642	0.719	-1.406	-0.443	1.704
C16	3.177	3.367	4.483	3.676	6.444
C17	3.186	3.375	4.493	3.685	6.460
C18	3.180	3.369	4.484	3.677	6.447
C19	3.173	3.362	4.476	3.671	6.435
C20	3.234	3.424	4.548	3.735	6.547
C21	3.226	3.416	4.540	3.727	6.534
C22	-0.363	-0.940	-0.999	-0.767	1.419
C23	0.984	1.041	1.718	1.248	2.237
C24	5.567	5.849	7.676	6.364	11.141
C25	5.550	5.833	7.658	6.347	11.111
C26	-0.363	-0.941	-1.000	-0.768	1.420
C27	0.995	1.052	1.731	1.260	2.257
C28	5.484	5.766	7.575	6.275	10.987

Table S22: DFT calculated hyperfine coupling tensors (in MHz) for the ^{13}C and ^{1}H nuclei in [ThCp$^{tt}_3$] (optimised structure)

Atom	A_1	A_2	A_3	A_{iso}	A_{Euclid}
C29	0.990	1.046	1.724	1.254	2.247
C30	-0.362	-0.937	-0.996	-0.765	1.415
C31	-0.361	-0.937	-0.996	-0.765	1.414
C32	0.984	1.041	1.717	1.248	2.236
C33	5.482	5.764	7.576	6.274	10.985
C34	5.443	5.729	7.527	6.233	10.913
C35	1.034	1.092	1.778	1.301	2.328
C36	-0.356	-0.938	-0.998	-0.764	1.415
C37	-0.355	-0.938	-0.998	-0.764	1.415
C38	1.028	1.085	1.770	1.294	2.316
C39	5.468	5.753	7.560	6.260	10.961
H1	1.174	-2.512	-3.163	-1.500	4.206
H2	0.233	-0.345	3.548	1.145	3.572
H3	0.234	-0.343	3.547	1.146	3.571
H4	1.174	-2.507	-3.154	-1.496	4.197
H5	0.229	-0.349	3.531	1.137	3.556
H6	0.222	-0.356	3.526	1.131	3.551
H7	1.167	-2.535	-3.194	-1.521	4.242
H8	0.245	-0.335	3.558	1.156	3.582
H9	0.235	-0.344	3.549	1.146	3.573
H10	0.264	0.473	1.330	0.689	1.436
H11	-0.388	-0.474	0.826	-0.012	1.028
H12	-0.310	-0.522	0.792	-0.013	0.998
H13	-0.537	-0.705	1.233	-0.003	1.518
H14	-1.288	-1.419	2.431	-0.092	3.096
H15	-0.454	-0.479	0.979	0.015	1.181
H16	-0.111	-0.411	1.783	0.420	1.833
H17	-2.411	-3.502	4.374	-0.513	6.100
H18	-0.995	-1.260	1.407	-0.282	2.134
H19	-0.118	-0.418	1.775	0.413	1.827
H20	-0.992	-1.256	1.405	-0.281	2.130
H21	-2.412	-3.501	4.368	-0.515	6.095
H22	-0.310	-0.523	0.792	-0.014	0.998
H23	-0.388	-0.474	0.826	-0.012	1.028
H24	0.264	0.473	1.330	0.689	1.436
H25	-0.453	-0.479	0.981	0.016	1.182
H26	-1.291	-1.423	2.435	-0.093	3.102
H27	-0.538	-0.706	1.235	-0.003	1.520
H28	-0.118	-0.416	1.767	0.411	1.819
H29	-2.394	-3.473	4.353	-0.505	6.062
H30	-0.986	-1.247	1.402	-0.277	2.119
H31	-0.536	-0.705	1.234	-0.003	1.518
H32	-1.288	-1.427	2.433	-0.094	3.101
H33	-0.455	-0.481	0.978	0.014	1.181
H34	0.263	0.471	1.326	0.687	1.432
H35	-0.386	-0.471	0.825	-0.011	1.025
H36	-0.310	-0.521	0.790	-0.013	0.996
H37	-0.310	-0.521	0.790	-0.014	0.995

Table S22: DFT calculated hyperfine coupling tensors (in MHz) for the ^{13}C and ^{1}H nuclei in [ThCp$^{tt}_3$] (optimised structure)

Atom	A_1	A_2	A_3	A_{iso}	A_{Euclid}
H38	-0.386	-0.471	0.825	-0.011	1.025
H39	0.262	0.469	1.325	0.685	1.429
H40	-0.454	-0.479	0.977	0.015	1.179
H41	-1.283	-1.422	2.425	-0.093	3.090
H42	-0.537	-0.706	1.234	-0.003	1.520
H43	-0.985	-1.246	1.401	-0.277	2.118
H44	-2.394	-3.474	4.356	-0.504	6.064
H45	-0.122	-0.421	1.764	0.407	1.817
H46	-0.132	-0.429	1.754	0.398	1.810
H47	-2.396	-3.474	4.329	-0.514	6.045
H48	-0.987	-1.245	1.401	-0.277	2.118
H49	-0.458	-0.484	0.984	0.014	1.188
H50	-0.540	-0.711	1.242	-0.003	1.530
H51	-1.314	-1.455	2.443	-0.109	3.133
H52	-0.389	-0.473	0.826	-0.012	1.028
H53	-0.315	-0.529	0.791	-0.018	1.002
H54	0.264	0.474	1.333	0.690	1.439
H55	-0.314	-0.529	0.791	-0.017	1.002
H56	-0.389	-0.474	0.826	-0.012	1.029
H57	0.265	0.475	1.334	0.691	1.440
H58	-1.311	-1.451	2.439	-0.108	3.127
H59	-0.540	-0.710	1.242	-0.003	1.529
H60	-0.458	-0.484	0.982	0.013	1.187
H61	-0.133	-0.431	1.756	0.397	1.813
H62	-0.989	-1.248	1.402	-0.278	2.121
H63	-2.401	-3.484	4.336	-0.516	6.059

Table S23: DFT calculated hyperfine coupling tensors (in MHz) for selected ^{1}H nuclei (near C_3 axis) in [ThCp$^{tt}_3$] (XRD structure top, optimised structure bottom)

	DFT					CASSCF				
Atom	A_1	A_2	A_3	A_{iso}	A_{Euclid}	A_1	A_2	A_3	A_{iso}	A_{Euclid}
H8	-1.174	-1.606	3.249	0.156	3.809	-0.929	-1.207	3.413	0.426	3.738
H11	-1.230	-1.955	4.521	0.446	5.077	-0.754	-1.201	4.372	0.806	4.596
H15	-0.943	-1.728	4.803	0.711	5.191	-0.530	-0.987	4.603	1.028	4.737
H26	-1.129	-2.126	6.404	1.049	6.841	-0.391	-1.018	6.030	1.540	6.127
H41	-0.795	-1.991	6.537	1.250	6.879	0.045	-0.760	6.327	1.871	6.372
H47	-1.074	-2.008	5.521	0.813	5.972	-0.496	-1.058	5.302	1.250	5.430
H51	-1.188	-1.414	2.694	0.031	3.266	-1.007	-1.176	2.822	0.213	3.219
H63	-0.967	-1.729	4.998	0.768	5.376	-0.515	-0.969	4.748	1.088	4.873
H17	-2.411	-3.502	4.374	-0.513	6.100	-0.456	-1.084	5.446	1.302	5.572
H21	-2.412	-3.501	4.368	-0.515	6.095	-0.458	-1.086	5.442	1.299	5.568
H29	-2.394	-3.473	4.353	-0.505	6.062	-0.462	-1.085	5.418	1.290	5.544
H44	-2.394	-3.474	4.356	-0.504	6.064	-0.462	-1.085	5.422	1.292	5.549
H47	-2.396	-3.474	4.329	-0.514	6.045	-0.466	-1.087	5.392	1.279	5.520
H63	-2.401	-3.484	4.336	-0.516	6.059	-0.462	-1.085	5.407	1.287	5.535

Table S24: DFT calculated hyperfine coupling tensors (in MHz) for selected ^{1}H nuclei (H_A) in [$ThCp^{tt}_3$] (XRD structure top, optimised structure bottom)

	DFT					CASSCF				
Atom	A_1	A_2	A_3	A_{iso}	A_{Euclid}	A_1	A_2	A_3	A_{iso}	A_{Euclid}
H1	1.887	-2.259	-3.072	-1.148	4.254	-1.114	-1.570	2.845	0.054	3.435
H22	1.853	-2.334	-3.281	-1.254	4.432	-1.085	-1.617	2.853	0.050	3.454
H43	1.816	-2.213	-3.033	-1.143	4.170	-1.089	-1.541	2.769	0.047	3.351
H1	1.174	-2.512	-3.163	-1.500	4.206	-1.023	-1.373	2.531	0.045	3.056
H4	1.174	-2.507	-3.154	-1.496	4.197	-1.022	-1.374	2.534	0.046	3.059
H7	1.167	-2.535	-3.194	-1.521	4.242	-1.023	-1.378	2.538	0.046	3.063

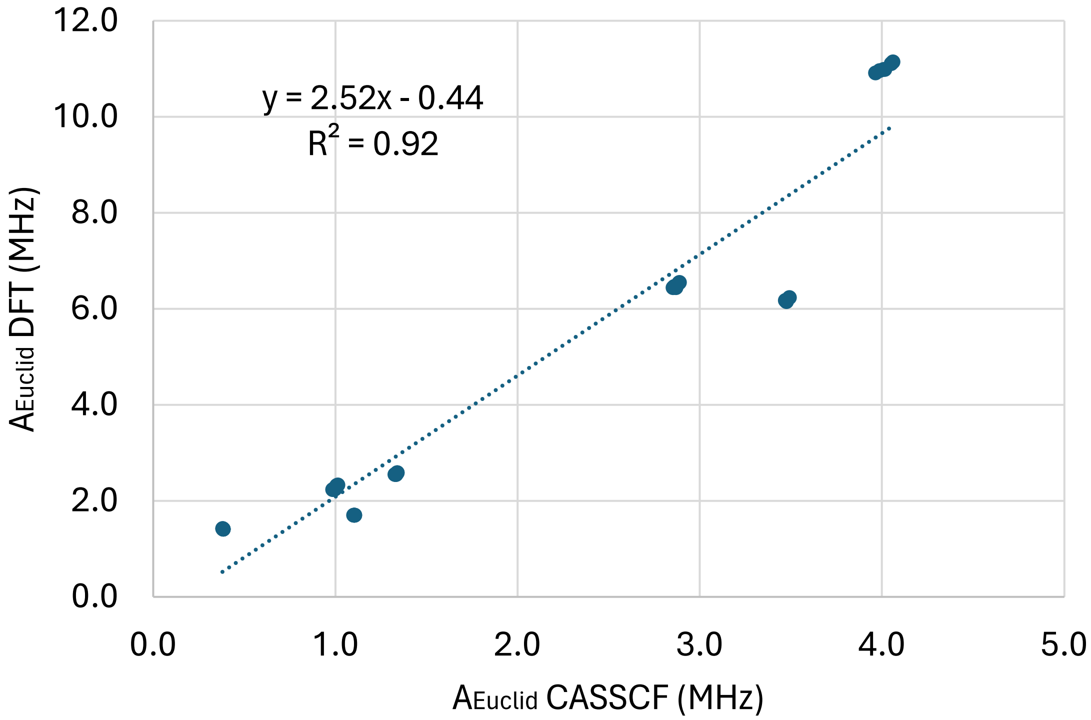

Figure S23: Correlation between CASSCF and DFT computed ^{13}C hyperfine couplings (A_{Euclid} = $\sqrt{A^2_{i,1} + A^2_{i,2} + A^2_{i,3}}$) for the optimised structure of [$ThCp^{tt}_3$].

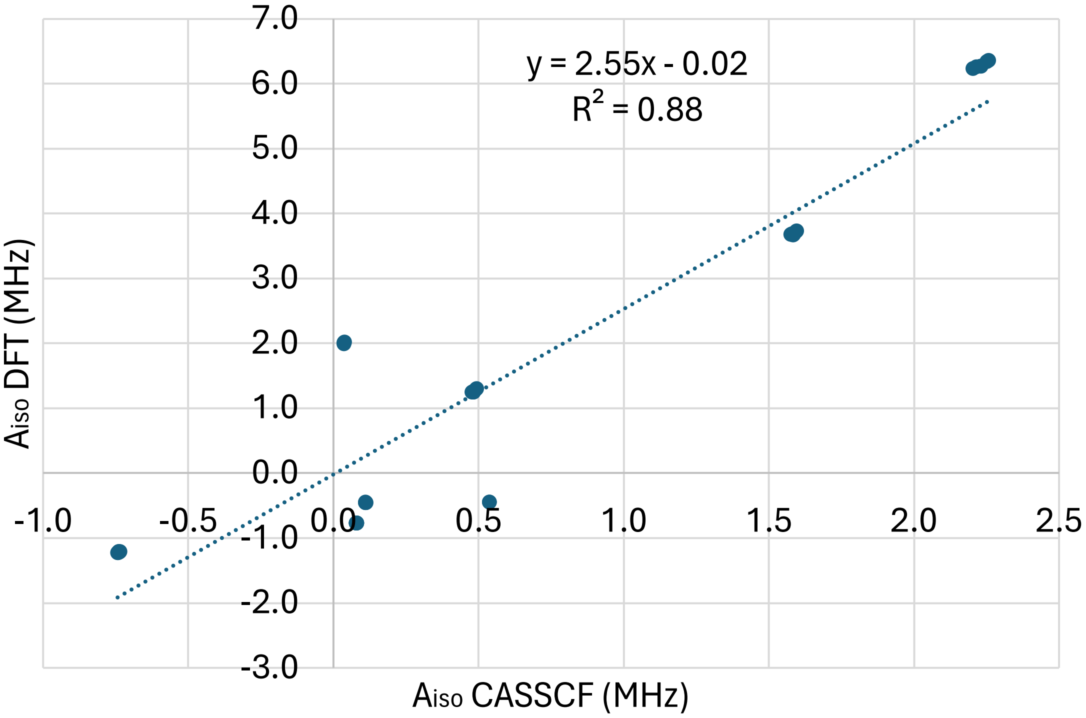

Figure S24: Correlation between CASSCF and DFT computed ^{13}C hyperfine couplings (A_{iso}) for the optimised structure of [$ThCp^{tt}_3$].

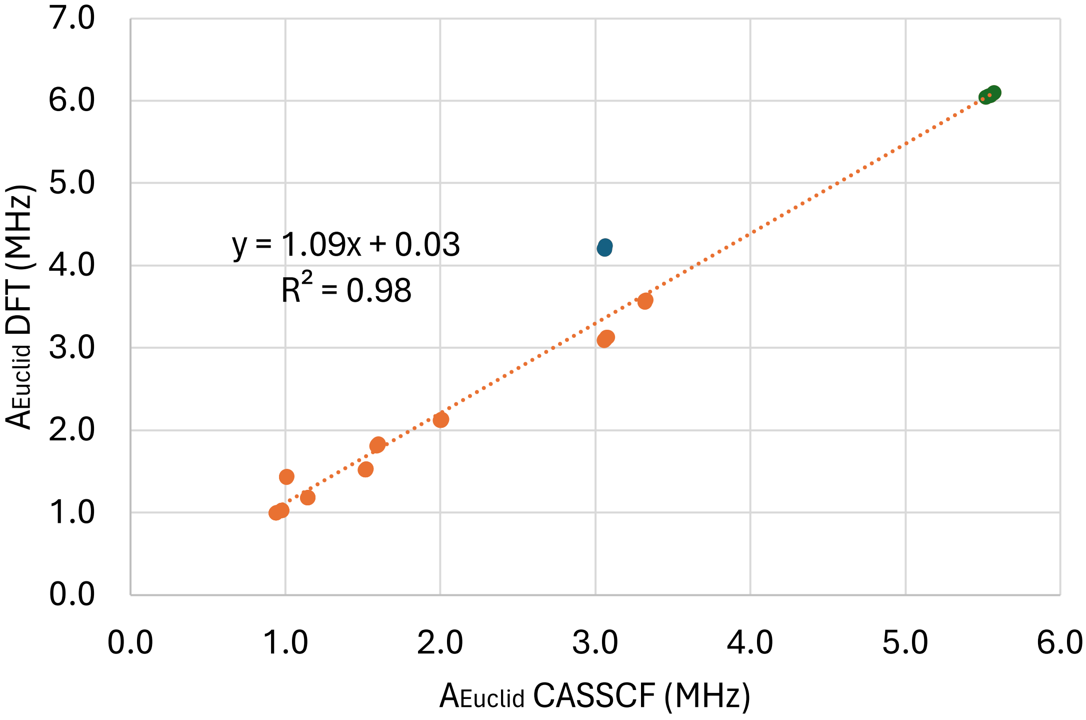

Figure S25: Correlation between CASSCF and DFT computed ^{1}H hyperfine couplings ($A_{Euclid} = \sqrt{A_{i,1}^2 + A_{i,2}^2 + A_{i,3}^2}$) for the optimised structure of [$ThCp^{tt}_3$]. Blue = H_A protons, green = C_3 protons, orange = all other protons.

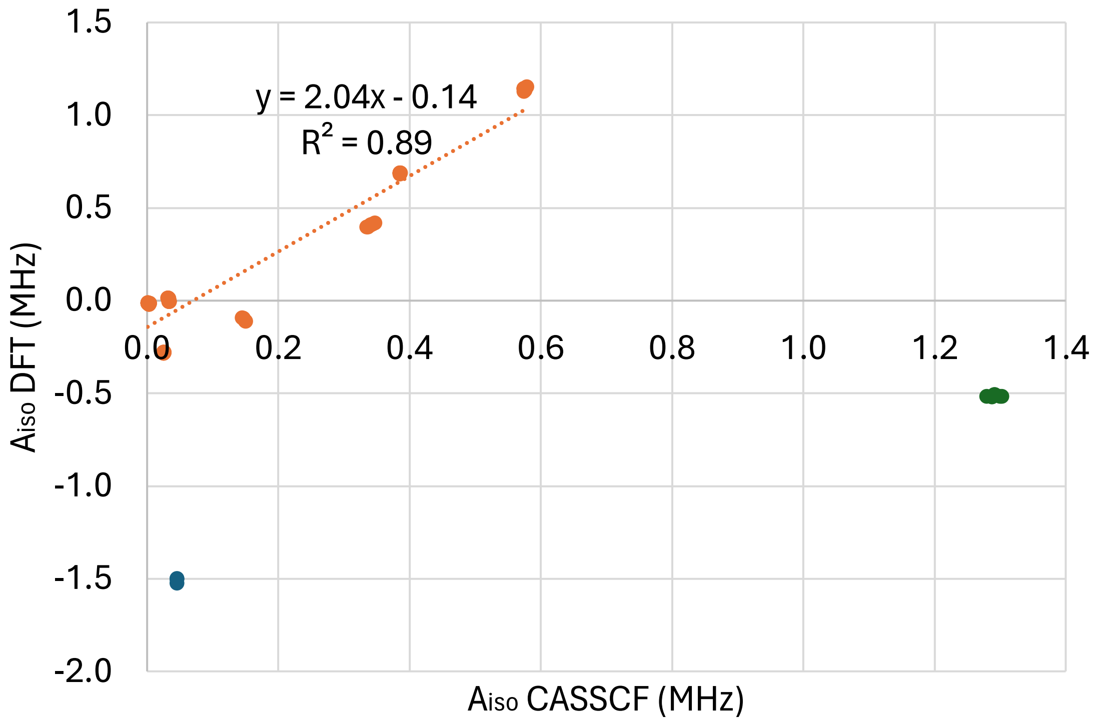

Figure S26: Correlation between CASSCF and DFT computed ^{1}H hyperfine couplings (A_{iso}) for the optimised structure of [$ThCp^{tt}_3$]. Blue = H_A protons, green = C_3 protons, orange = all other protons.

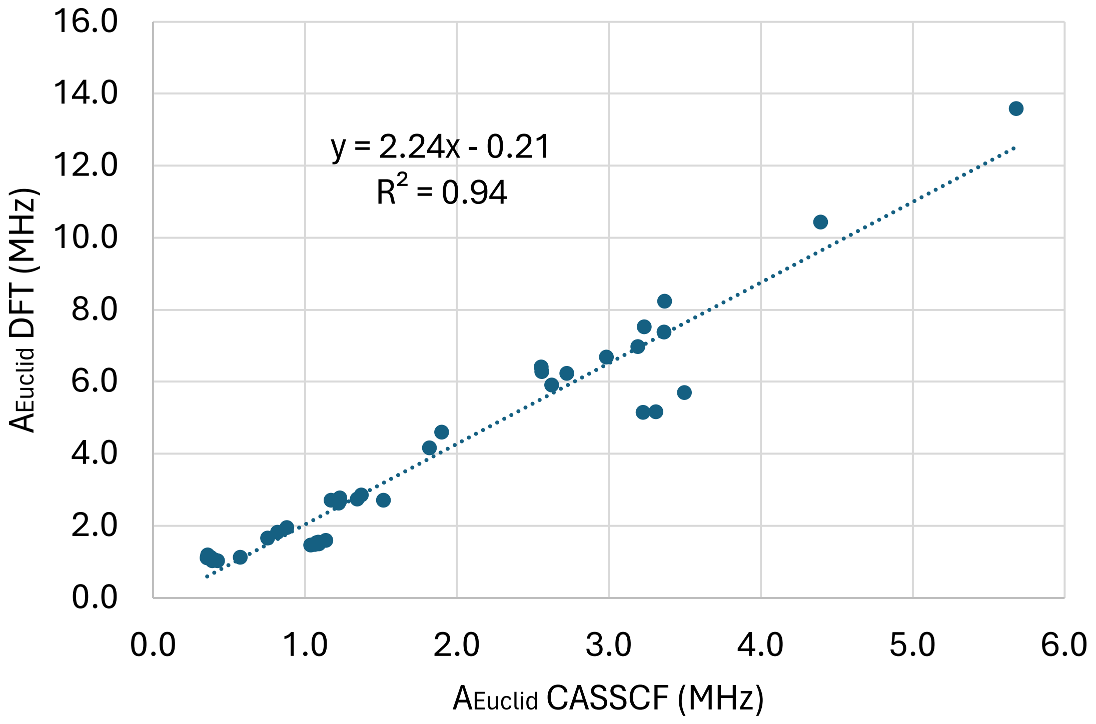

Figure S27: Correlation between CASSCF and DFT computed ^{13}C hyperfine couplings (A_{Euclid} = $\sqrt{A_{i,1}^2 + A_{i,2}^2 + A_{i,3}^2}$) for the XRD structure of [$ThCp^{tt}_3$].

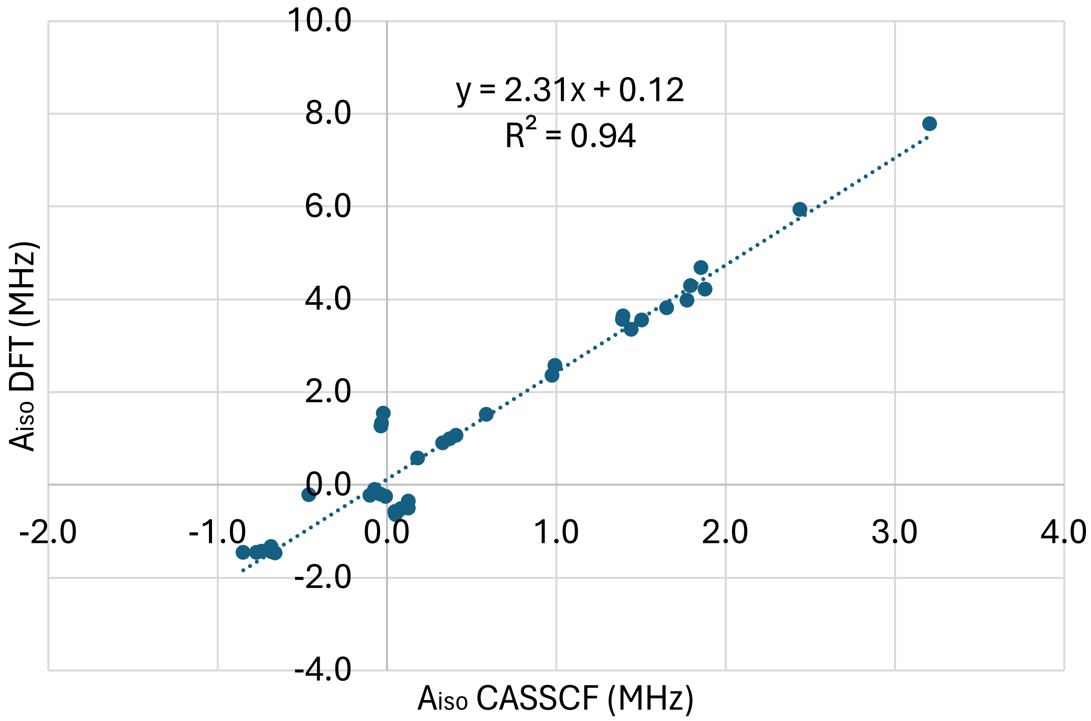

Figure S28: Correlation between CASSCF and DFT computed ^{13}C hyperfine couplings (A_{iso}) for the XRD structure of [ThCp$_3^{tt}$].

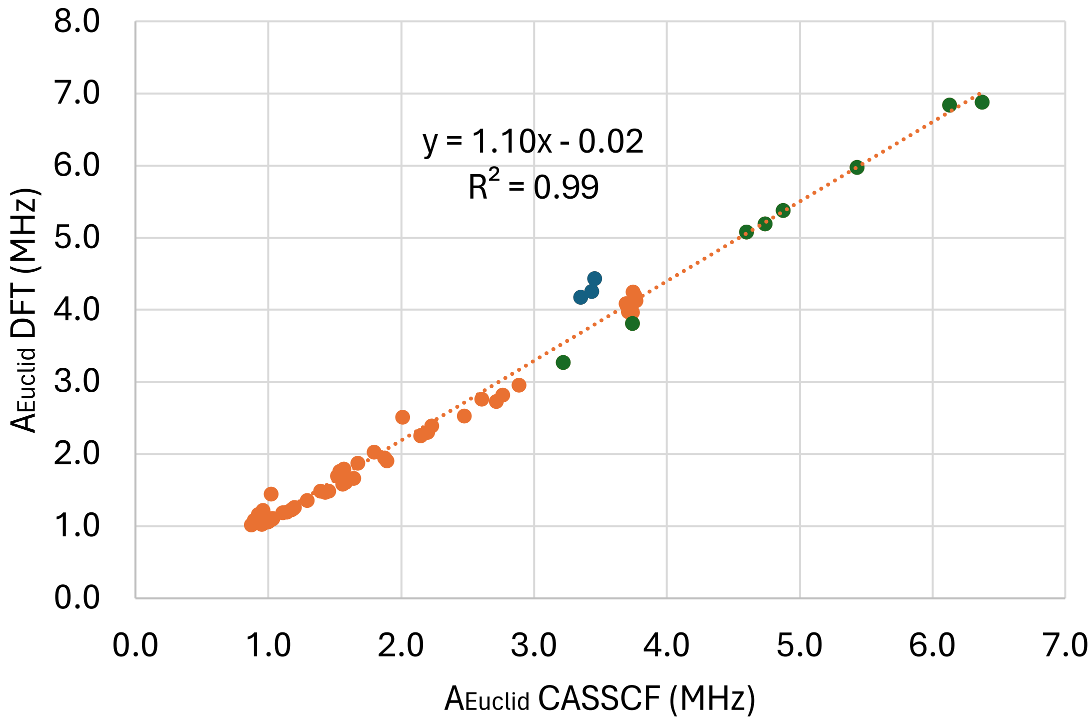

Figure S29: Correlation between CASSCF and DFT computed ^{1}H hyperfine couplings ($A_{Euclid} = \sqrt{A_{i,1}^2 + A_{i,2}^2 + A_{i,3}^2}$) for the XRD structure of [ThCp$_3^{tt}$]. Blue = H_A protons, green = C_3 protons, orange = all other protons.

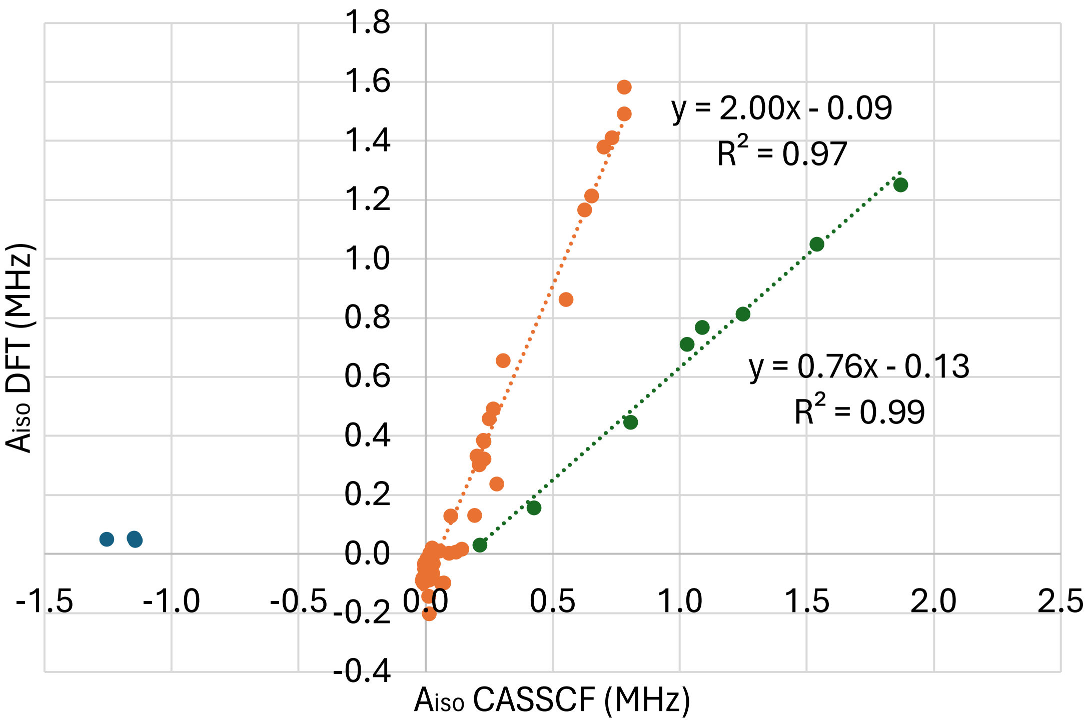

Figure S30: Correlation between CASSCF and DFT computed ^{1}H hyperfine couplings (A_{iso}) for the XRD structure of [$ThCp_3^{tt}$]. Blue = H_A protons, green = C_3 protons, orange = all other protons.

S8 Additional HYSCORE simulations

S8.1 HYSCORE simulation parameters

HYSCORE simulations for [ThCp$^{tt}_3$] are carried out at $B_0 = 351.6$ mT ($g_{\|}$) and at $B_0 = 366.3$ mT ($g_{\perp}$), with $\tau = 130$ ns and t_1 and t_2 incremented from 0.2 μs to 5.3 μs in steps of 0.02 μs (256 points). 181 orientations (knots) are computed explicitly, using a spectrometer frequency of 9.614 GHz and a microwave excitation bandwidth of 62.5 MHz. Experimental spectra are rendered using 30 contour levels in the following ranges: 0.018−1.5 (^{13}C HYSCORE, $g_{\perp}$), 0.05−1.5 (^{1}H HYSCORE, $g_{\perp}$) and 0.15−1.5 (^{13}C HYSCORE and ^{1}H HYSCORE, $g_{\|}$). Simulated spectra are rendered using 20 contour levels between 0.15 and 2 (^{13}C HYSCORE) and between 0.5 and 5 (^{1}H HYSCORE).

^{1}H HYSCORE simulations for [UCp$^{tt}_3$] are carried out at $B_0 = 244.3$ mT (g_{x}) and at $B_0 = 450.4$ mT (g_{y}), with $\tau = 200$ ns and t_1 and t_2 incremented from 0.2 μs to 5.3 μs in steps of 0.02 μs (256 points). 31 orientations (knots) are computed explicitly, using a spectrometer frequency of 9.723 GHz and a microwave excitation bandwidth of 62.5 MHz. Experimental spectra are rendered using 30 contour levels between 0.3 and 2, while simulated spectra are shown using 20 contour levels between 0.15 and 2. We note that, for both complexes, we employ the same HYSCORE simulation parameters as Formanuik *et al.*

The increased computational cost of HYSCORE simulations for [ThCp$^{tt}_3$], particularly ^{13}C HYSCORE, precludes the entire set of C(Cp) and C(^{t}Bu) nuclei; hence, complex selection criteria are needed. Due to the axially-symmetric *g*-values of [ThCp$^{tt}_3$], we assert that equivalent nuclei associated with different cyclopentadienyl rings give rise to similar HYSCORE features. In practice, this assertion only proves true for state-averaged results; EPR parameters from state-specific calculations yield subtly different HYSCORE signals for the three Cp^{tt} groups in the solid-state.

In addition to splitting ligand nuclei by Cp^{tt} group, we also investigate selections of three C(Cp), with one in-plane nucleus (C_A-type), one out-of-plane substituted nucleus (C_D/C_E-type) and one out-of-plane unsubstituted nucleus (C_B/C_C-type). The omission of two C(Cp) nuclei appears to have a minor impact – a slight decrease in intensity – on the simulated HYSCORE spectra, and we conclude that the $3\times$C(Cp1) simulation provides the best balance between computational cost and accuracy. To complete the set of representative ^{13}C nuclei, we additionally include one tertiary C(^{t}Bu) from the Cp1 group (C6 in the XRD

structure) and one primary C(ᵗBu) (C26 in the XRD structure); both are selected based on their distance from Th in the crystal structure.

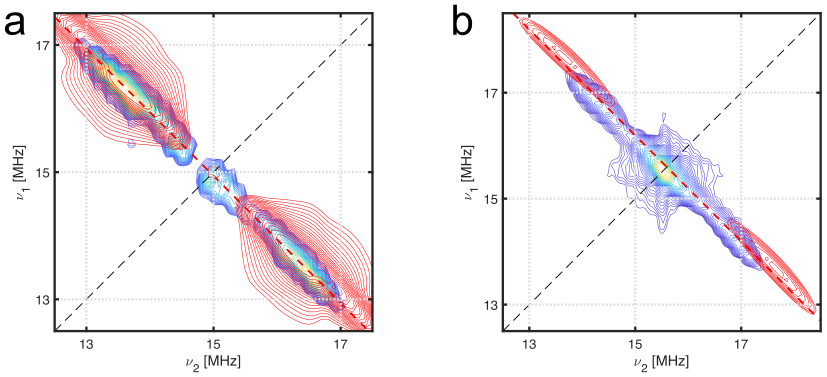

Figure S31: Experimental HYSCORE spectra in the ^{1}H region for [ThCp$_3^{tt}$] (blue contours); **a**: $B_0 = 351.6$ mT ($g_\parallel$), **b**: $B_0 = 366.3$ mT ($g_\perp$). Simulations (red contours) use EPR parameters calculated from SS-RASSCF(39,38) (XRD structure) and include all three H(Cp1) atoms and the H(ᵗBu) atom lying closest to the Th center in the XRD structure (H41).

S8.2 Simulated ^{13}C HYSCORE spectra of [ThCp$^{tt}_3$]

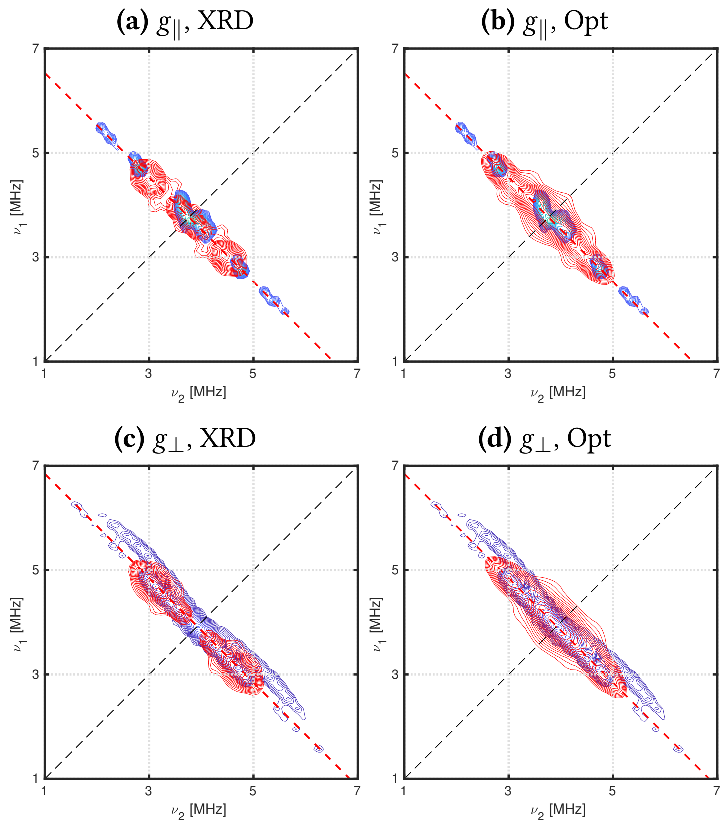

Figure S32: Experimental HYSCORE spectra in the ^{13}C region for [ThCp$^{tt}_3$] (blue contours); left: $B_0 = 366.3$ mT ($g_{||}$), right: $B_0 = 351.6$ mT ($g_{\perp}$). Simulations (red contours) use EPR parameters calculated from SA-(27,36)-SO. All simulations include five C(Cp) atoms from one ligand, two 1° C(^{t}Bu), and two 3° C(^{t}Bu) nuclei.

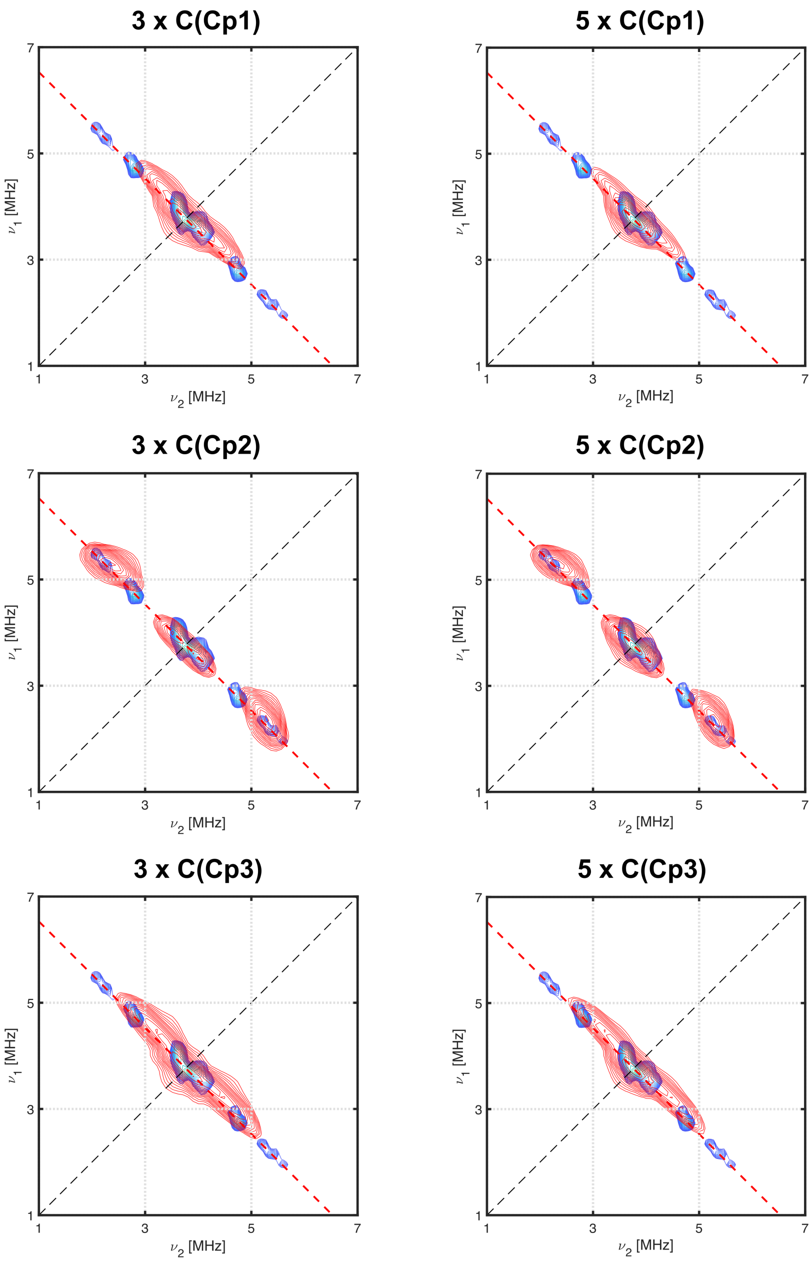

Figure S33: Experimental HYSCORE spectra (blue contours) in the ^{13}C region for [ThCp$^{tt}_3$], measured at $B_0 = 351.6$ mT ($g_{\|}$). Simulations (red contours) use EPR parameters calculated from SS-RASSCF(39,38) (XRD structure); only parameters for selected C(Cp) nuclei are included in the HYSCORE simulation.

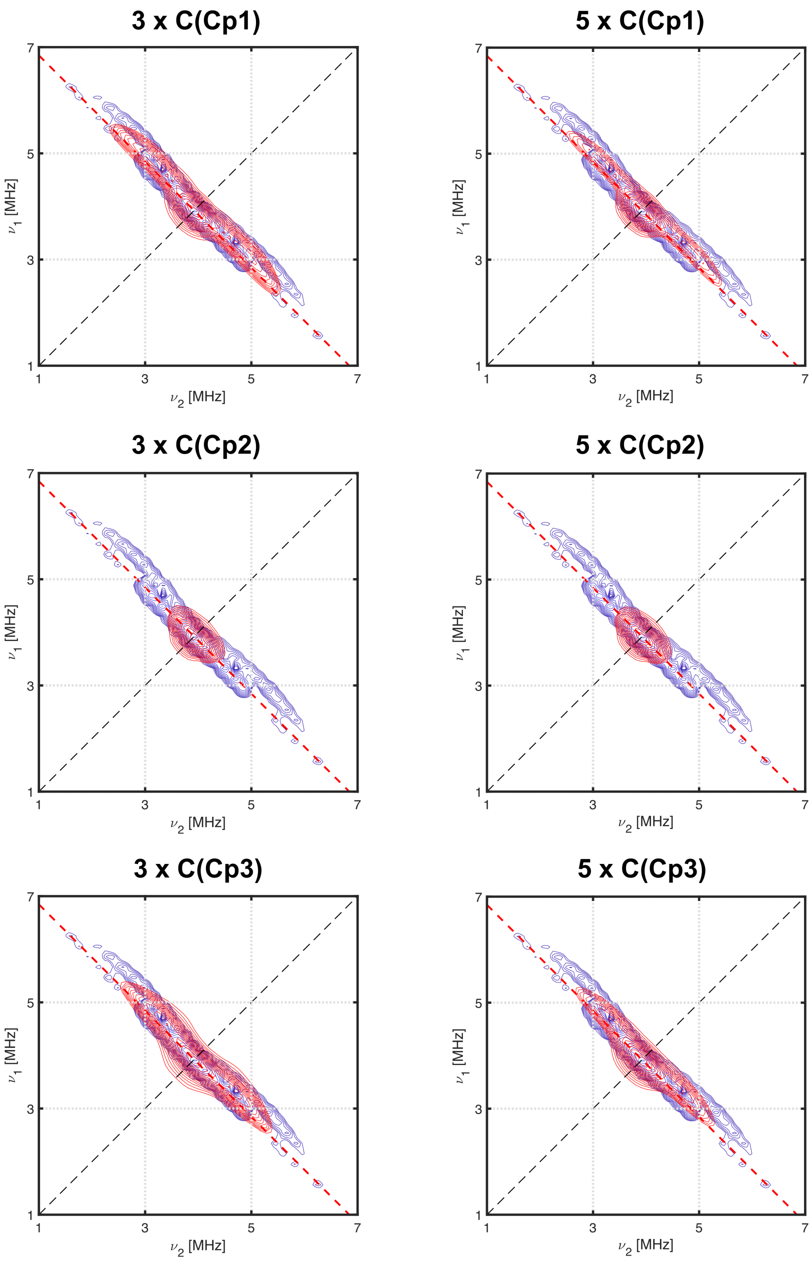

Figure S34: Experimental HYSCORE spectra (blue contours) in the ^{13}C region for [ThCp$^{tt}_3$], measured at $B_0 = 366.3$ mT ($g_\perp$). Simulations (red contours) use EPR parameters calculated from SS-RASSCF(39,38) (XRD structure); only parameters for selected C(Cp) nuclei are included in the HYSCORE simulation.

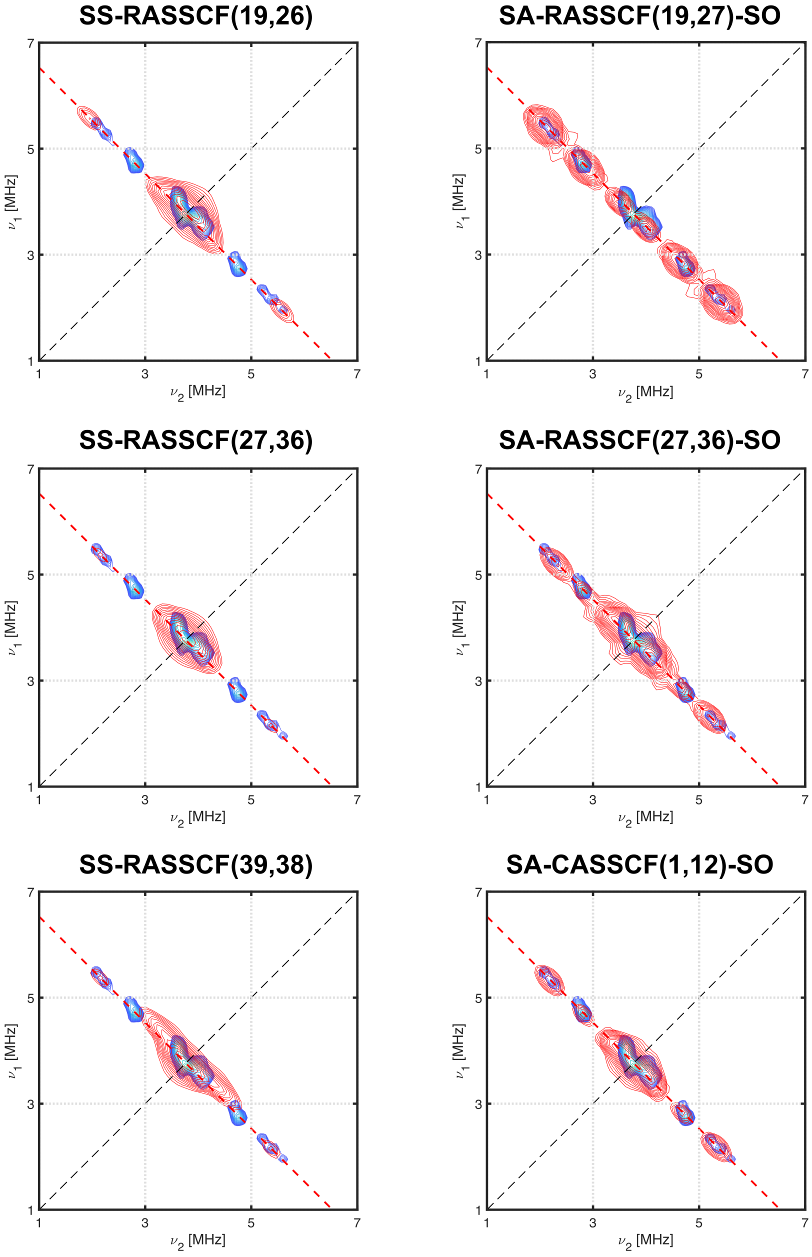

Figure S35: Experimental HYSCORE spectra in the ^{13}C region at $B_0 = 351.6$ mT ($g_{\|}$) for [ThCp$_3^{tt}$] (blue contours). HYSCORE simulations (XRD structure; red contours) include HFC matrices of 5 representative nuclei: three C(Cp), one 3° C(^{t}Bu) and one 1° C(^{t}Bu).

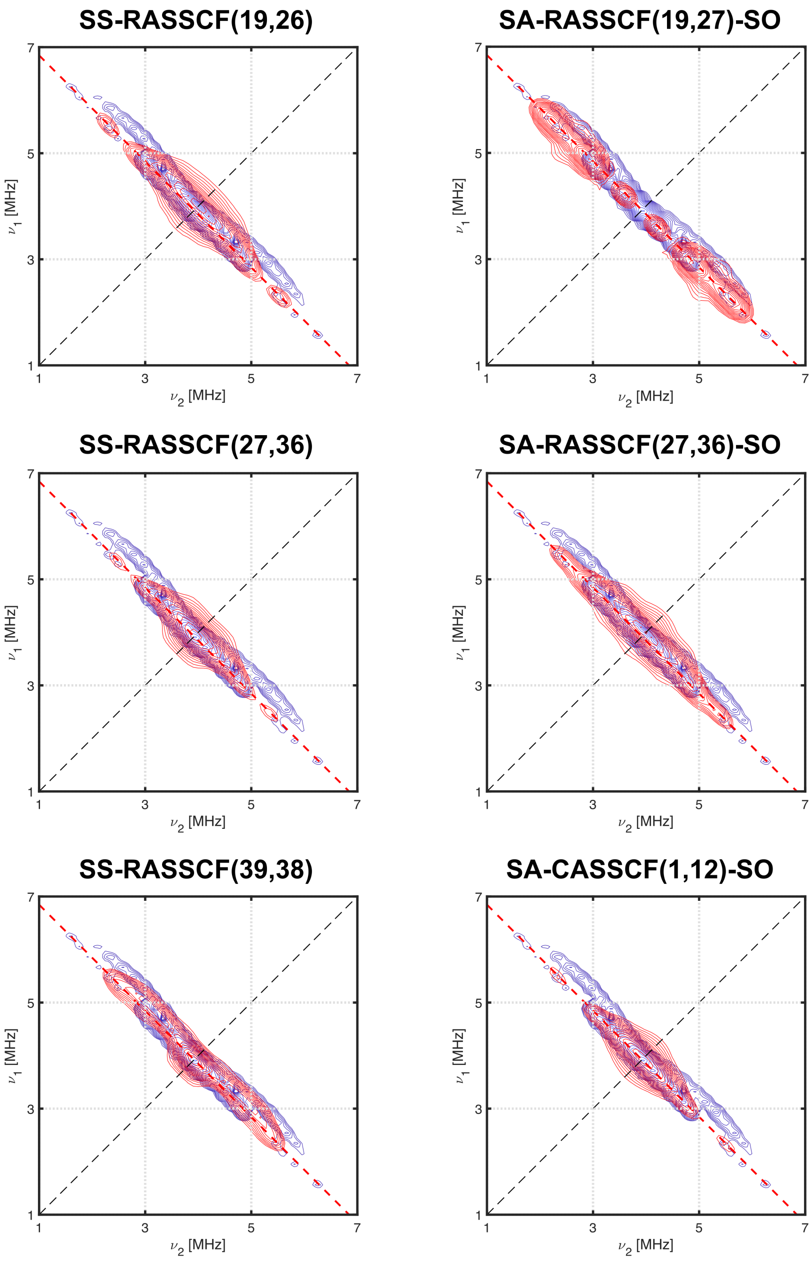

Figure S36: Experimental HYSCORE spectra in the ^{13}C region at $B_0 = 366.3$ mT ($g_\perp$) for [ThCp$_3^{tt}$] (blue contours). HYSCORE simulations (XRD structure; red contours) include HFC matrices of 5 representative nuclei: three C(Cp), one 3° C(^{t}Bu) and one 1° C(^{t}Bu).

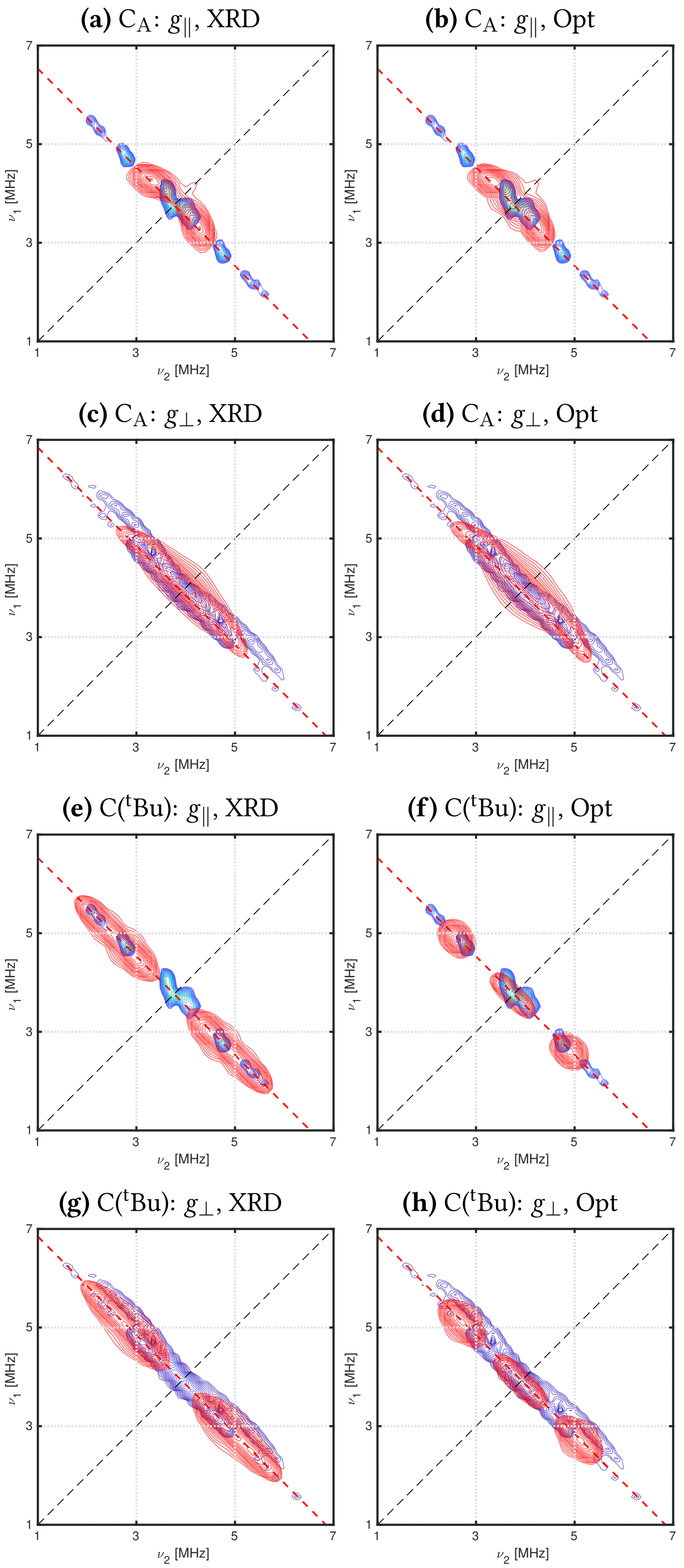

Figure S37: Experimental HYSCORE spectra (blue contours) in the ^{13}C region for [$ThCp^{tt}_3$], measured at $B_0 = 351.6$ mT ($g_{||}$). Simulations (red contours) use EPR parameters calculated from SA-RASSCF(19,27)-SO; all three C_A nuclei are included in simulations (a) - (d) and all six C(^{t}Bu) nuclei are included in (e) - (h).

S8.3 Simulated ^{1}H HYSCORE spectra of [ThCp$^{tt}_3$]

We use similar criteria as the ^{13}C case to select a set of ^{1}H nuclei, comprising three H(Cp) from one Cp^{tt} group and one H(^{t}Bu). However, HYSCORE simulations that include atom H41 (XRD structure label), having the smallest Th-H(^{t}Bu) distance, result in unusually intense HYSCORE features, as well as poor agreement with the spectrum measured at $g_\perp$ (Figure S31). This is most likely a consequence of the uncharacteristically large spin population associated with H41 (Table S26); it is unclear whether this is a limitation of the minimal basis used for H(^{t}Bu) in our electronic structure calculations, or whether such high spin densities are a feature of the crystal structure, but are not prevalent in frozen solution. Nevertheless, we observe significant improvement in the accuracy of HYSCORE simulations upon replacing HFC parameters of H41 with those calculated for H8, which has a Mulliken spin population closer to typical H(^{t}Bu) values.

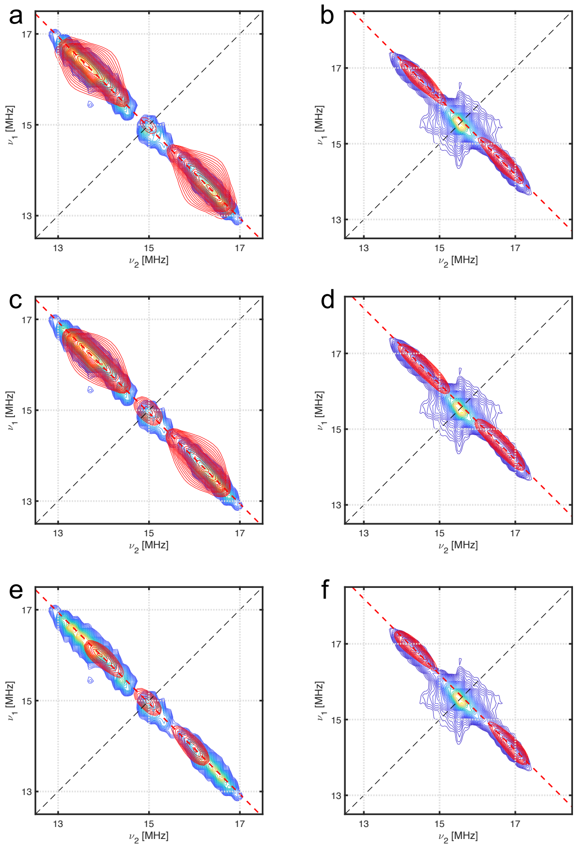

Figure S38: Experimental HYSCORE spectra in the ^{1}H region for [ThCp$^{tt}_3$] (blue contours); left: $B_0 = 366.3$ mT ($g_\|$), right: $B_0 = 351.6$ mT ($g_\perp$). Simulations (red contours) use EPR parameters calculated from SS-RASSCF(39,38) (XRD structure) and include all three H(Cp) atoms from one cyclopentadienyl ring and one H(^{t}Bu) atom (H8 in the XRD structure). **a**, **b**: H(Cp1)+H(^{t}Bu); **c**, **d**: H(Cp2)+H(^{t}Bu); **e**, **f**: H(Cp3)+H(^{t}Bu)

S8.4 Simulated ^{1}H HYSCORE spectra of [UCp$^{tt}_{3}$]

Preliminary calculations defined two sets of H(Cp) nuclei based on distances measured in the crystal structure: set **1** includes all in-plane (H_A-type) nuclei and the three out-of-plane (H_B/H_C-type) nuclei closest to U, while set **2** includes all in-plane nuclei and the three out-of-plane nuclei located farthest from U. Given the geometry fluctuations in solution and assuming no drastic change in the U-Cp centroid distance, we expect the HYSCORE signal of H(Cp) in solution to be roughly an average between the two sets.

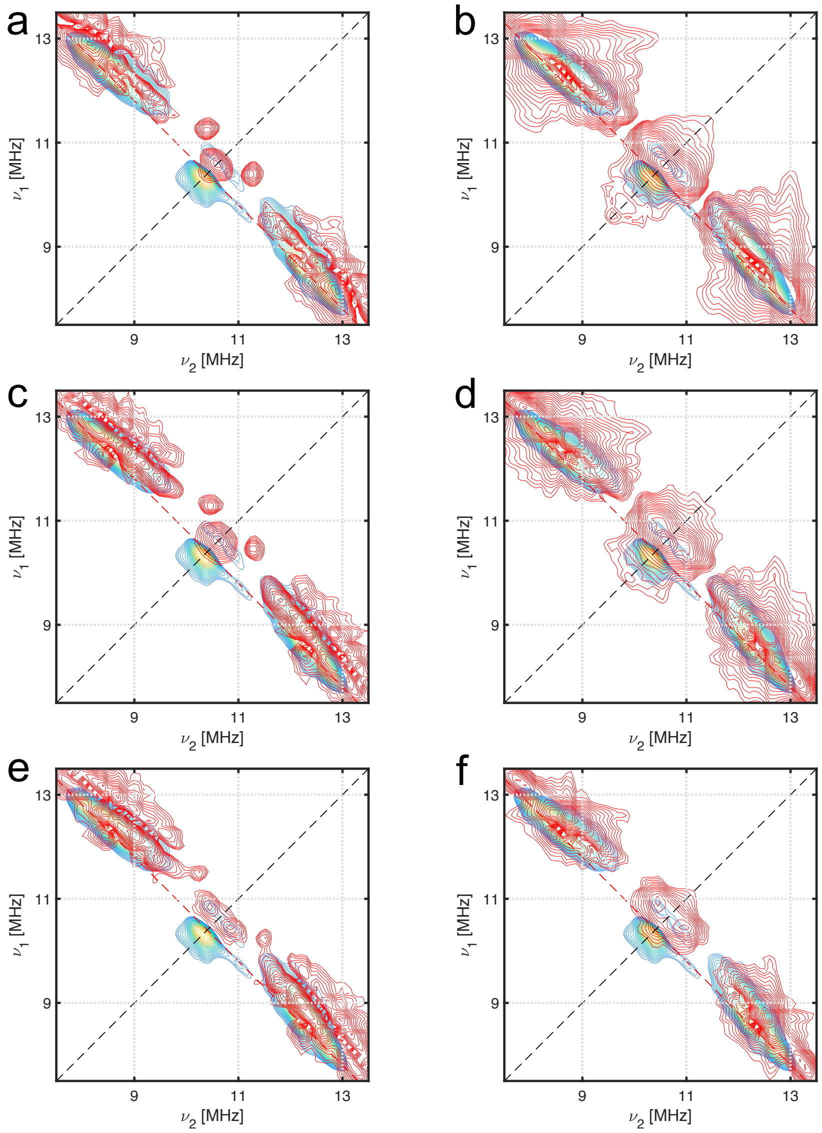

Figure S39: [UCp$^{tt}_{3}$] HYSCORE spectra, ^{1}H region, B_0 = 244.3 mT (near g_x). Simulations (XRD structure; red) include H(Cp) nuclei from set **1** (left) and set **2** (right). **a**, **b**: RASSCF(21,30)-SO; **c**, **d**: RASCI(29,34)-SO; **e**, **f**: RASCI(29,35)-SO.

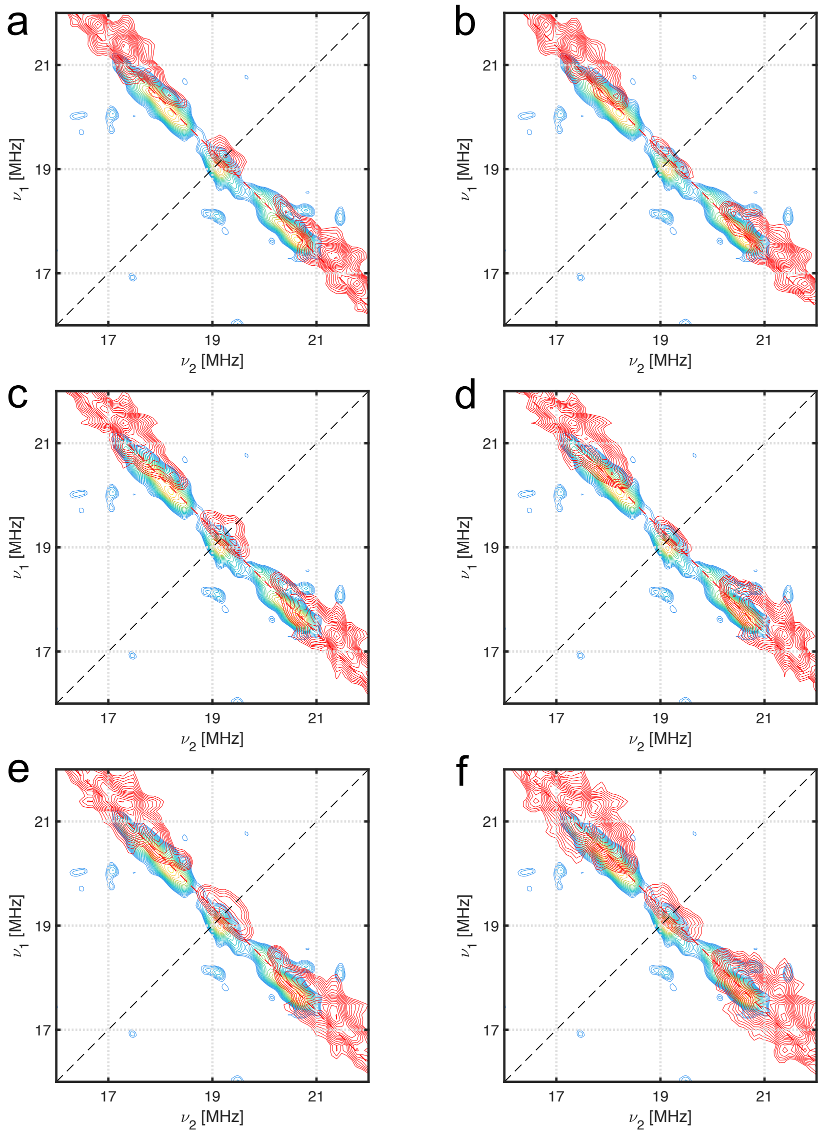

Figure S40: [UCp_3^{tt}] HYSCORE spectra, ^{1}H region, $B_0 = 450.4$ mT (near g_y). Simulations (XRD structure; red) include H(Cp) nuclei from set **1** (left) and set **2** (right). **a**, **b**: RASSCF(21,30)-SO; **c**, **d**: RASCI(29,34)-SO; **e**, **f**: RASCI(29,35)-SO.

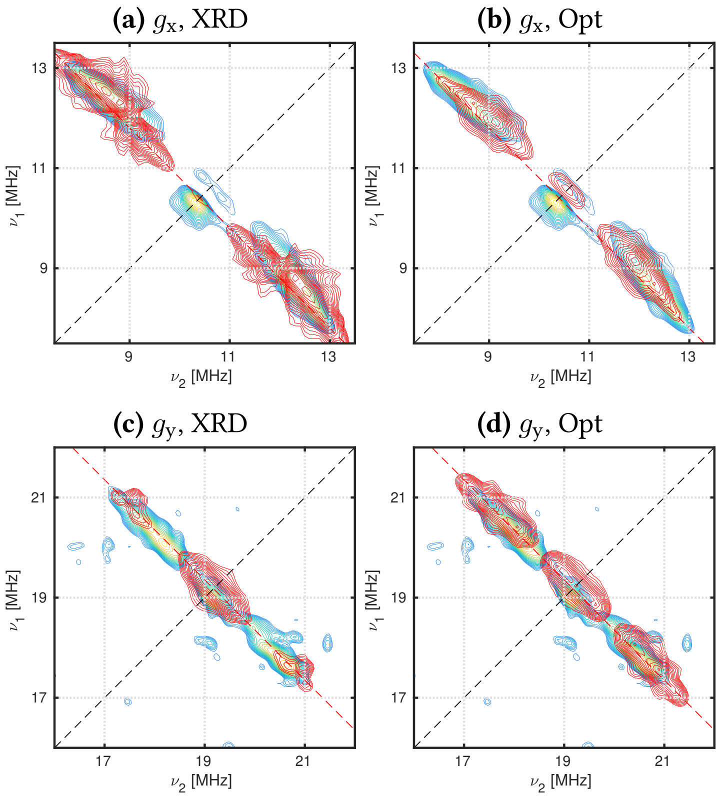

Figure S41: Experimental HYSCORE spectra in the ^{1}H region for [UCp$_3^{tt}$] (blue contours); top: $B_0 = 244.3$ mT (near g_x) and bottom:$B_0 = 450.4$ mT (near g_y). Simulations (red) for the XRD structures (a,c) include twelve nuclei: nine H(Cp) and the three closest H(^{t}Bu) and the Opt structures (b,d) include nine H(Cp) nuclei. Simulations used HFCCs computed with a CASSCF(3,7)-SO wave function.

S9 SO states of [UCp$_3^{tt}$]

Table S25: Composition of the lowest-energy Kramers doublet (SO states 1 and 2) of [UCp$_3^{tt}$] in terms of quartet SF states. SF state indices are assigned in order of increasing energy, such that SF state 1 is the lowest-energy quartet SF state. Note that only the lowest 6 quartet SF states make significant contributions to the ground Kramers doublet in all calculations, and that SF state 4 has the most significant contribution in all calculations that include ligand correlation effects.

	SO state 1		SO state 2	
**Calculation**	**SF state**	**Weight (%)**	**SF state**	**Weight (%)**
CASSCF(3,7)-SO	1	16.65	1	16.65
	2	15.61	2	15.61
	3	14.91	3	14.91
	4	14.47	4	14.47
	5	12.98	5	12.98
RASSCF(21,30)-SO	4	23.42	4	23.42
	5	20.60	5	20.60
	6	12.39	6	12.39
	3	11.61	3	11.61
	2	9.52	2	9.52

Calculation	SO state 1		SO state 2	
	SF state	Weight (%)	SF state	Weight (%)
RASCI(29,34)-SO	4	23.66	4	23.66
	5	20.91	5	20.91
	6	14.08	6	14.08
	3	10.48	3	10.48
	2	8.49	2	8.49
RASCI(29,35)-SO	4	23.49	4	23.49
	5	20.57	5	20.57
	6	13.61	6	13.61
	3	10.63	3	10.63
	2	8.95	2	8.95

S10 Mulliken spin population analysis

Table S26: Mulliken spin populations of [$ThCp^{tt}_3$] atoms. Columns **a**-**f** correspond to electronic structure calculations; **a**: SA-CASSCF(1,12)-SO; **b**: SS-RASSCF(19,26); **c**: SA-RASSCF(19,27)-SO; **d**: SS-RASSCF(27,36); **e**: SA-RASSCF(27,36)-SO; **f**: SS-RASSCF(39,38). Atom labels are consistent with the crystal structure reported in reference [17]. For the 1°C(^{t}Bu) and H(^{t}Bu) atom groups, the minimum and maximum spin populations are reported. Atoms C26 and H8 are included in the HYSCORE simulations reported in the main text, while H41 is the H(^{t}Bu) atom closest to the Th center.

**Atom**	**a**	**b**	**c**	**d**	**e**	**f**
Th	0.9367	0.9784	0.9674	0.9625	0.9574	0.9617
**C(Cp1)**						
C1	0.0073	0.0037	0.0038	0.0051	0.0052	0.0044
C2	0.0074	0.0092	0.0107	0.0083	0.0090	0.0089
C3	0.0071	0.0034	0.0035	0.0047	0.0046	0.0040
C4	0.0013	-0.0022	-0.0016	-0.0009	-0.0006	-0.0003
C5	0.0012	-0.0025	-0.0019	-0.0012	-0.0008	-0.0005
**C(Cp2)**						
C14	0.0072	0.0035	0.0035	0.0049	0.0049	0.0042
C15	0.0079	0.0099	0.0115	0.0090	0.0097	0.0095
C16	0.0077	0.0041	0.0042	0.0053	0.0054	0.0046
C17	0.0014	-0.0016	-0.0010	-0.0006	-0.0002	0.0000
C18	0.0012	-0.0027	-0.0022	-0.0013	-0.0010	-0.0006
**C(Cp3)**						
C27	0.0075	0.0042	0.0044	0.0055	0.0054	0.0047
C28	0.0072	0.0091	0.0106	0.0082	0.0089	0.0087
C29	0.0062	0.0023	0.0024	0.0038	0.0038	0.0032
C30	0.0012	-0.0025	-0.0020	-0.0011	-0.0008	-0.0005
C31	0.0013	-0.0022	-0.0015	-0.0010	-0.0006	-0.0003
**3°C(^{t}Bu)**						
C6	-0.0002	-0.0004	-0.0002	-0.0003	-0.0002	-0.0003
C10	-0.0002	-0.0004	-0.0002	-0.0003	-0.0002	-0.0003
C19	-0.0004	-0.0006	-0.0004	-0.0005	-0.0004	-0.0005
C23	-0.0004	-0.0006	-0.0005	-0.0005	-0.0004	-0.0005
C32	-0.0003	-0.0004	-0.0003	-0.0004	-0.0003	-0.0004
C36	-0.0002	-0.0004	-0.0003	-0.0003	-0.0003	-0.0003
**H(Cp1)**						
H1	0.0010	0.0011	0.0011	0.0011	0.0010	0.0011
H2	-0.0014	-0.0017	-0.0016	-0.0014	-0.0014	-0.0014
H3	-0.0020	-0.0024	-0.0022	-0.0020	-0.0019	-0.0020
**H(Cp2)**						
H22	0.0011	0.0011	0.0011	0.0011	0.0010	0.0011
H23	-0.0011	-0.0014	-0.0013	-0.0011	-0.0012	-0.0011
H24	-0.0019	-0.0023	-0.0021	-0.0019	-0.0018	-0.0019

**Atom**	**a**	**b**	**c**	**d**	**e**	**f**
**H(Cp3)**						
H43	0.0009	0.0010	0.0010	0.0009	0.0009	0.0009
H44	-0.0015	-0.0017	-0.0017	-0.0014	-0.0015	-0.0014
H45	-0.0016	-0.0020	-0.0019	-0.0016	-0.0016	-0.0016
**1°C(^{t}Bu)**						
C26	0.0003	0.0001	0.0002	0.0001	0.0002	0.0001
min	0.0000	-0.0001	-0.0001	-0.0001	-0.0001	-0.0001
max	0.0005	0.0004	0.0005	0.0003	0.0004	0.0004
**H(^{t}Bu)**						
H8	-0.0003	-0.0004	-0.0004	-0.0003	-0.0003	-0.0003
H41	-0.0012	-0.0013	-0.0012	-0.0012	-0.0011	-0.0012
min	-0.0012	-0.0013	-0.0012	-0.0012	-0.0011	-0.0012
max	0.0003	0.0003	0.0002	0.0003	0.0003	0.0001

Table S27: Mulliken spin populations of [UCp^{tt}_3] atoms, as determined from CASSCF(3,7)-SO.

Atom	SF state 1	SF state 2	SF state 3	SF state 4	SF state 5	SF state 6
U	2.9780	2.9746	2.9744	2.9677	2.9675	2.9671
**C(Cp1)**						
C1	0.0015	0.0016	0.0019	0.0020	0.0024	0.0021
C2	0.0014	0.0011	0.0015	0.0012	0.0011	0.0012
C3	0.0014	0.0015	0.0018	0.0020	0.0024	0.0023
C4	0.0015	0.0020	0.0016	0.0029	0.0023	0.0027
C5	0.0016	0.0020	0.0018	0.0027	0.0025	0.0024
**C(Cp2)**						
C14	0.0016	0.0014	0.0021	0.0018	0.0023	0.0021
C15	0.0014	0.0013	0.0013	0.0013	0.0010	0.0011
C16	0.0014	0.0014	0.0021	0.0020	0.0023	0.0023
C17	0.0015	0.0017	0.0019	0.0020	0.0030	0.0026
C18	0.0015	0.0017	0.0019	0.0018	0.0029	0.0027
**C(Cp3)**						
C27	0.0014	0.0022	0.0012	0.0026	0.0018	0.0022
C28	0.0013	0.0014	0.0009	0.0009	0.0011	0.0011
C29	0.0016	0.0022	0.0013	0.0025	0.0019	0.0019
C30	0.0016	0.0019	0.0020	0.0028	0.0024	0.0026
C31	0.0015	0.0018	0.0020	0.0029	0.0024	0.0027
**H(Cp1)**						
H1	0.0000	-0.0001	0.0000	0.0000	0.0000	0.0000
H2	-0.0001	-0.0001	-0.0001	-0.0001	0.0000	0.0000
H3	-0.0001	-0.0001	-0.0001	0.0000	0.0000	0.0000
**H(Cp2)**						
H22	-0.0001	0.0000	-0.0001	0.0000	0.0000	0.0000
H23	-0.0001	-0.0001	-0.0001	-0.0001	0.0000	-0.0001
H24	-0.0002	-0.0001	-0.0001	0.0000	-0.0001	0.0000
**H(Cp3)**						
H43	-0.0001	0.0000	-0.0001	0.0000	0.0000	0.0000
H44	-0.0001	-0.0001	-0.0001	0.0000	-0.0001	0.0000
H45	-0.0001	-0.0001	-0.0001	0.0000	-0.0001	-0.0001

Table S28: Mulliken spin populations of [UCp^{tt}_3] atoms, as determined from RASCI(29,35)-SO.

**Atom**	**SF state 1**	**SF state 2**	**SF state 3**	**SF state 4**	**SF state 5**	**SF state 6**
U	3.0474	3.0430	3.0429	3.0332	3.0332	3.0336
**C(Cp1)**						
C1	-0.0034	-0.0038	-0.0031	-0.0032	-0.0043	-0.0031
C2	-0.0023	-0.0031	-0.0017	-0.0031	-0.0028	-0.0032
C3	-0.0039	-0.0039	-0.0034	-0.0034	-0.0034	-0.0018
C4	-0.0033	-0.0016	-0.0036	-0.0010	-0.0004	-0.0013
C5	-0.0027	-0.0016	-0.0029	-0.0009	-0.0004	-0.0016
**C(Cp2)**						
C14	-0.0030	-0.0041	-0.0019	-0.0028	-0.0033	-0.0026
C15	-0.0026	-0.0026	-0.0032	-0.0038	-0.0025	-0.0040
C16	-0.0033	-0.0042	-0.0020	-0.0023	-0.0034	-0.0021
C17	-0.0031	-0.0026	-0.0023	-0.0007	-0.0020	-0.0010
C18	-0.0034	-0.0028	-0.0025	-0.0014	-0.0021	-0.0002
**C(Cp3)**						
C27	-0.0037	-0.0017	-0.0048	-0.0031	-0.0013	-0.0033
C28	-0.0022	-0.0021	-0.0030	-0.0030	-0.0038	-0.0021
C29	-0.0033	-0.0023	-0.0046	-0.0035	-0.0026	-0.0049
C30	-0.0025	-0.0024	-0.0013	-0.0006	0.0000	-0.0009
C31	-0.0033	-0.0034	-0.0016	-0.0004	-0.0011	-0.0014
**H(Cp1)**						
H1	-0.0002	-0.0002	-0.0001	-0.0001	-0.0002	-0.0001
H2	-0.0004	-0.0003	-0.0003	-0.0002	-0.0003	-0.0002
H3	-0.0003	-0.0002	-0.0002	-0.0001	-0.0001	-0.0001
**H(Cp2)**						
H22	-0.0002	-0.0001	-0.0003	-0.0002	-0.0001	-0.0002
H23	-0.0004	-0.0004	-0.0003	-0.0002	-0.0003	-0.0003
H24	-0.0004	-0.0003	-0.0004	-0.0002	-0.0001	-0.0001
**H(Cp3)**						
H43	-0.0002	-0.0002	-0.0002	-0.0002	-0.0002	-0.0001
H44	-0.0004	-0.0003	-0.0004	-0.0002	-0.0001	-0.0002
H45	-0.0003	-0.0003	-0.0002	-0.0001	-0.0001	-0.0002

S11 Orbital decomposition analysis

Below, we show HFC orbital decomposition diagrams generated by Hyperion for ligand atoms representative of different C(Cp) and H(Cp) local environments within [$AnCp_3^{tt}$]. The vertices represent RASSCF-optimised MOs, which are linear combinations of AOs from all atoms in [$AnCp_3^{tt}$]. As such, their labels are only approximate, as they represent an idealised picture where there is no mixing between An and ligand MOs. Although most labels could be unambiguously assigned, we observe strong mixing between Th $6p_{x,y}$ and Cp π_1 orbitals, which may complicate the interpretation of the orbital decomposition. We note that Hyperion's orbital decomposition scheme only takes into account the spin-dependent (FC+SD) part of the HFC operator, therefore the significant PSO interactions are not included in this breakdown.

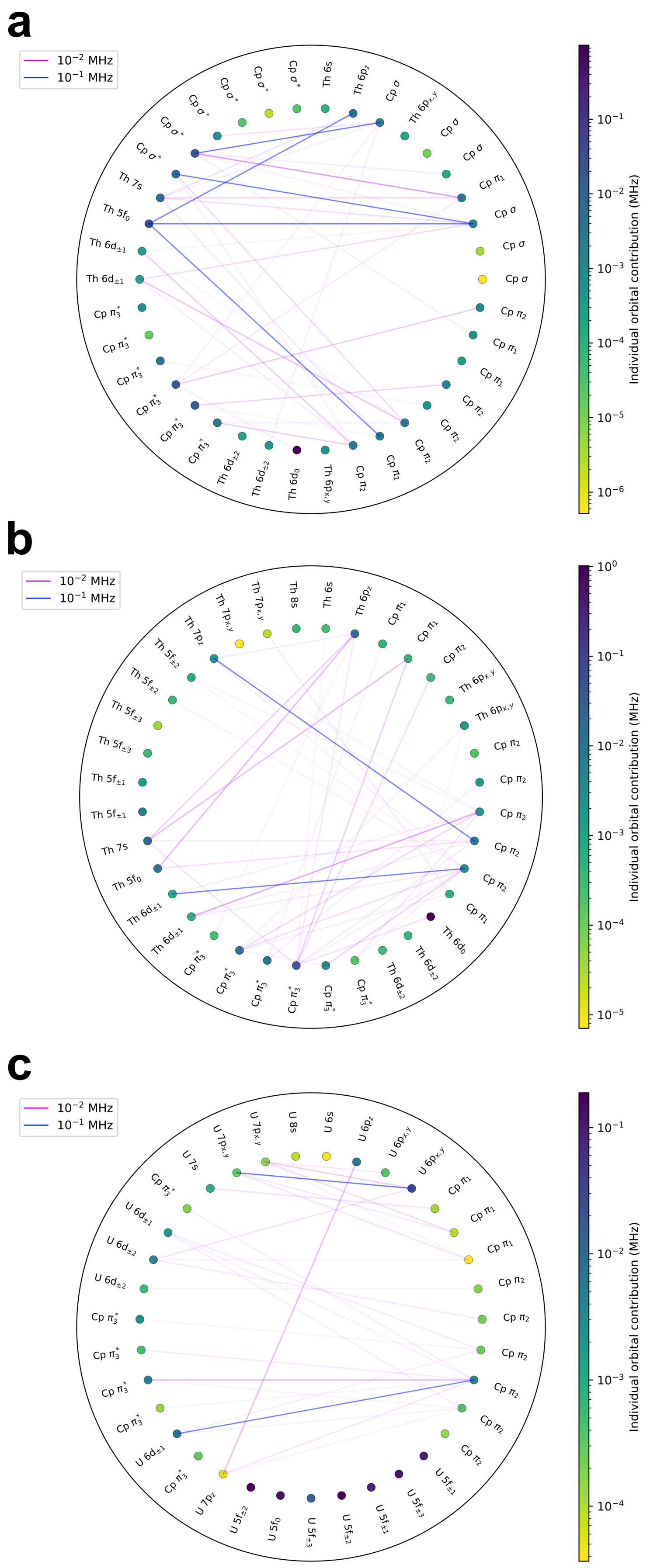

Figure S42: HFC orbital decomposition diagrams for nucleus C1 (as labelled in the XRD structure). Only pairwise contributions $> 10^{-2}$ MHz are shown. **a**: [ThCp$^{tt}_3$], SF state 1 ($S = 1/2$), SS-RASSCF(39,38); **b**: [ThCp$^{tt}_3$], SF state 1 ($S = 1/2$), SS-RASSCF(27,36); **c**: [UCp$^{tt}_3$], SF state 4 ($S = 3/2$), SS-RASCI(29,35)

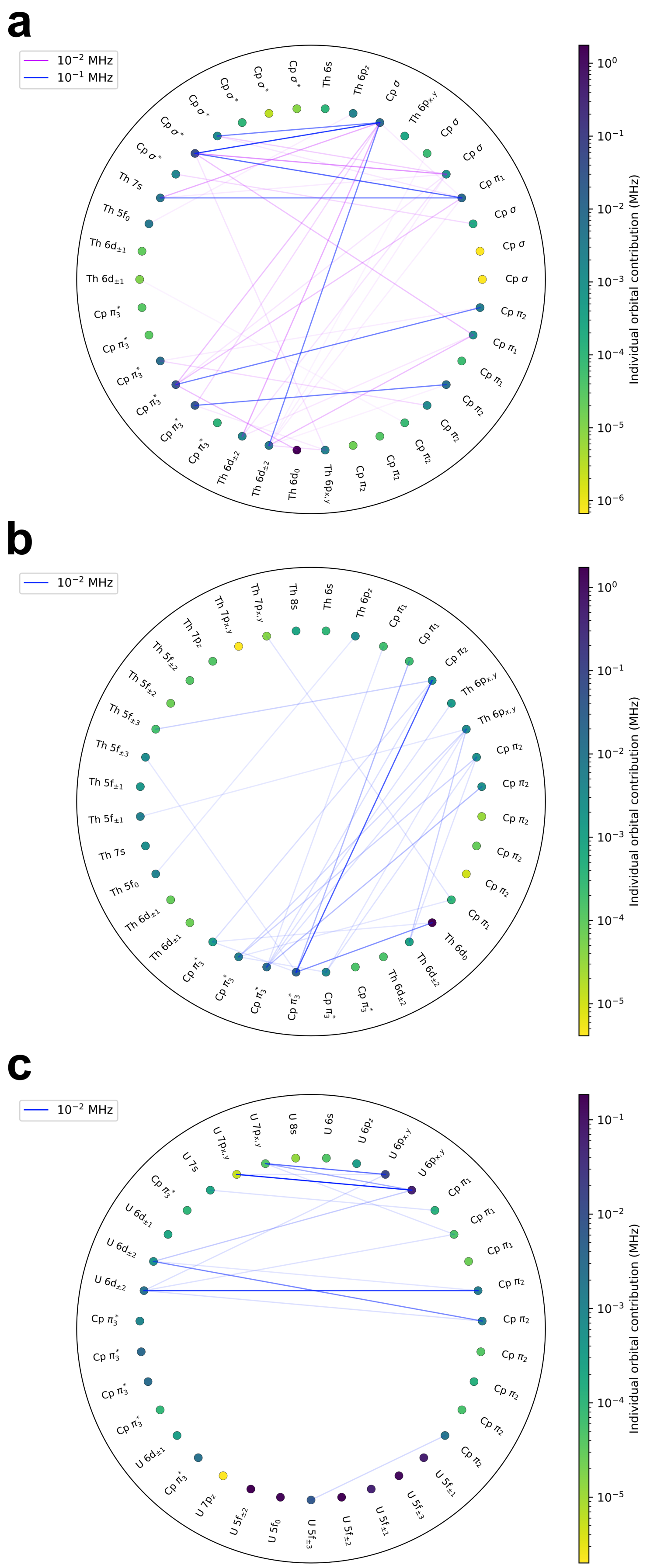

Figure S43: HFC orbital decomposition diagrams for nucleus C2 (as labelled in the XRD structure). Only pairwise contributions $> 10^{-2}$ MHz are shown. **a**: [ThCp_3^{tt}], SF state 1 ($S = 1/2$), SS-RASSCF(39,38); **b**: [ThCp_3^{tt}], SF state 1 ($S = 1/2$), SS-RASSCF(27,36); **c**: [UCp_3^{tt}], SF state 4 ($S = 3/2$), SS-RASCI(29,35)

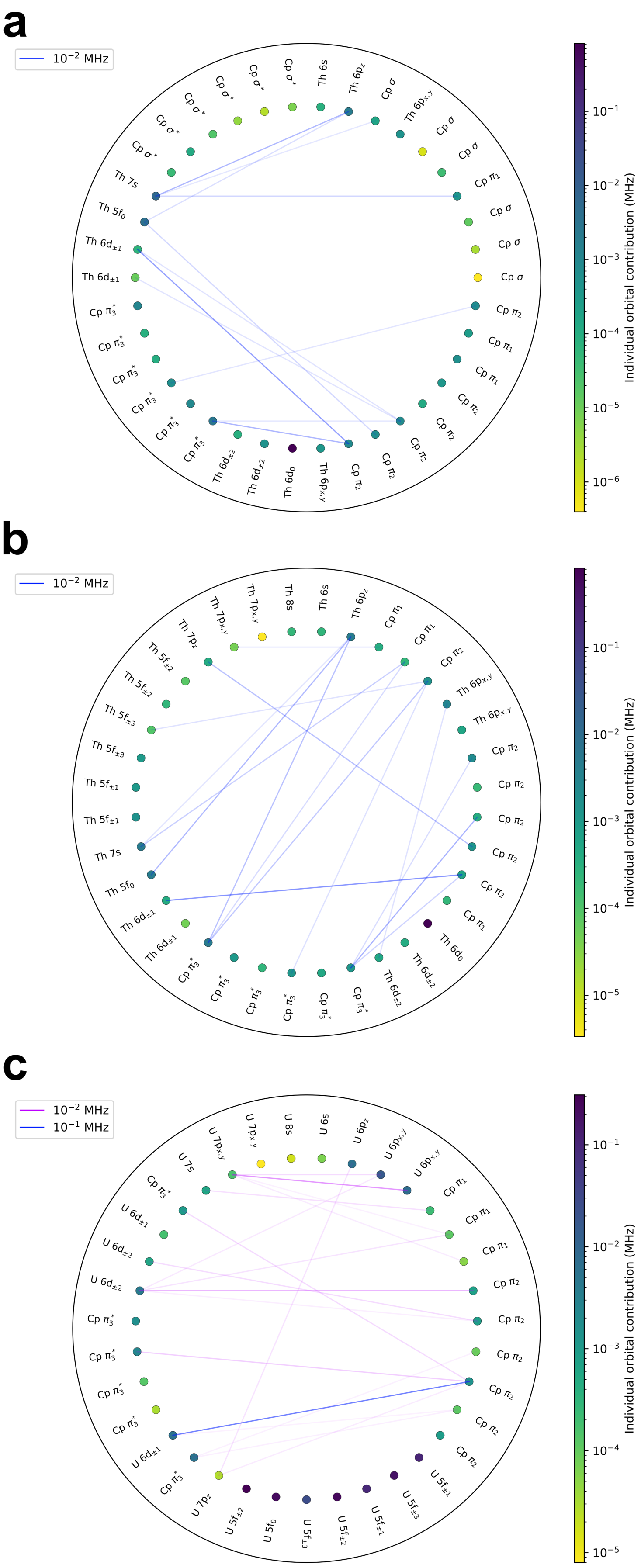

Figure S44: HFC orbital decomposition diagrams for nucleus C4 (as labelled in the XRD structure). Only pairwise contributions $> 10^{-2}$ MHz are shown. **a**: [ThCp$^{tt}_3$], SF state 1 ($S = 1/2$), SS-RASSCF(39,38); **b**: [ThCp$^{tt}_3$], SF state 1 ($S = 1/2$), SS-RASSCF(27,36); **c**: [UCp$^{tt}_3$], SF state 4 ($S = 3/2$), SS-RASCI(29,35)

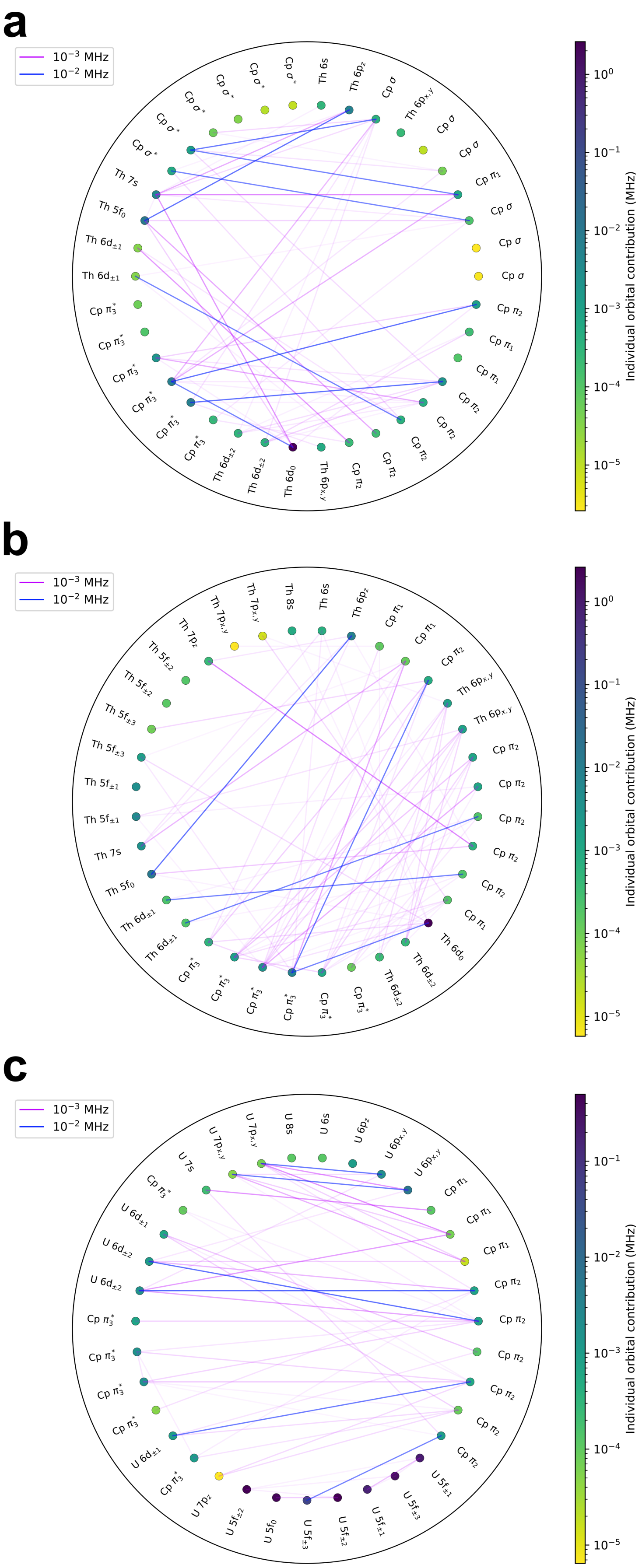

Figure S45: HFC orbital decomposition diagrams for nucleus H1 (as labelled in the XRD structure). Only pairwise contributions $> 10^{-3}$ MHz are shown. **a**: [ThCp$^{tt}_3$], SF state 1 ($S = 1/2$), SS-RASSCF(39,38); **b**: [ThCp$^{tt}_3$], SF state 1 ($S = 1/2$), SS-RASSCF(27,36); **c**: [UCp$^{tt}_3$], SF state 4 ($S = 3/2$), SS-RASCI(29,35)

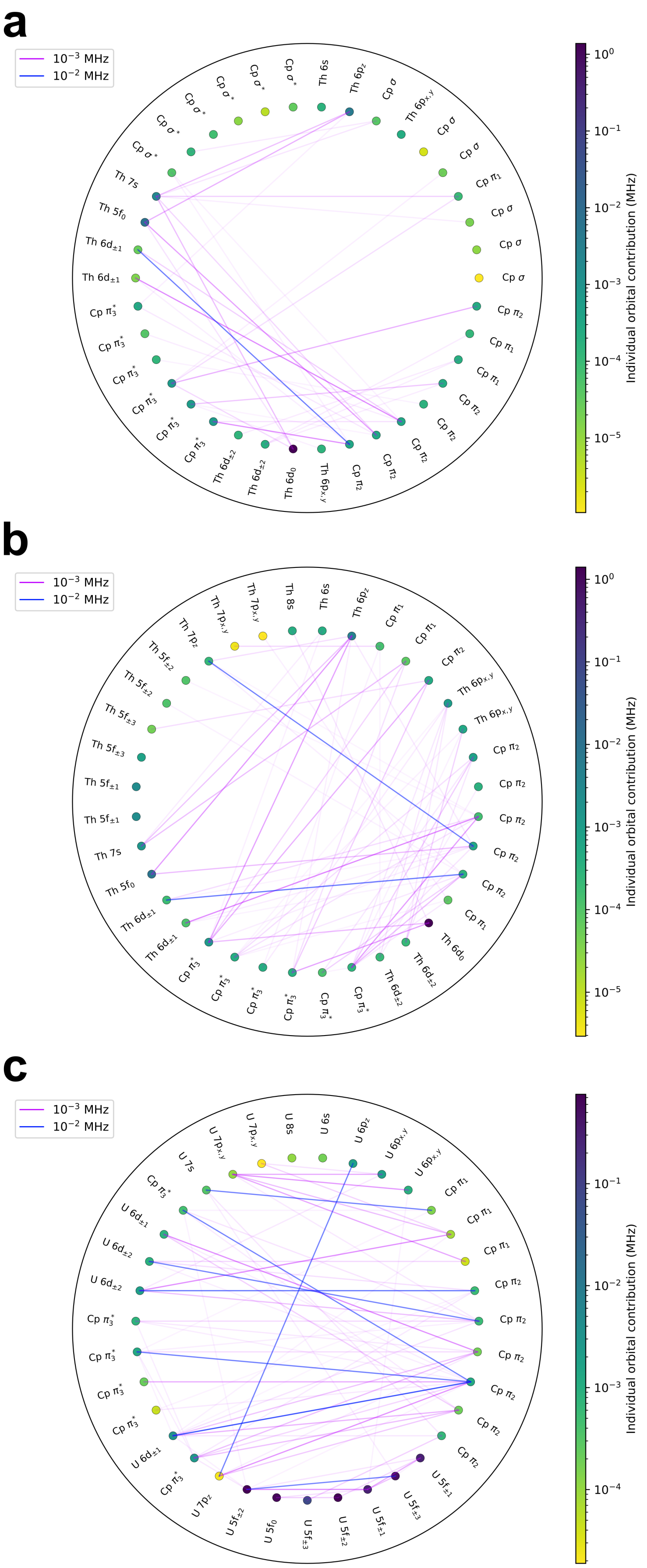

Figure S46: HFC orbital decomposition diagrams for nucleus H2 (as labelled in the XRD structure). Only pairwise contributions $> 10^{-3}$ MHz are shown. **a**: [ThCp$^{tt}_3$], SF state 1 ($S = 1/2$), SS-RASSCF(39,38); **b**: [ThCp$^{tt}_3$], SF state 1 ($S = 1/2$), SS-RASSCF(27,36); **c**: [UCp$^{tt}_3$], SF state 4 ($S = 3/2$), SS-RASCI(29,35)

S12 Paramagnetic Spin-Orbit Contribution to Hyperfine Coupling

Table S29: The approximate average (per atom: C or H) percent contribution of the paramagnetic spin-orbit term to the hyperfine coupling for [ThCp$^{tt}_3$] and [UCp$^{tt}_3$] with various active spaces using the XRD structure.

**Active space**	^{1}H	^{13}C
CASSCF(1,12)-SO	9.4	11.0
SA(3)-RASSCF(19,27)-SO	6.8	10.1
SA(3)-RASSCF(27,36)-SO	5.4	7.8
CASSCF(3,7)-SO	68.0	-
RASSCF(21,30)-SO	68.0	-
RASCI(29,34)-SO	68.0	-
RASCI(29,35)-SO	68.0	-

References

(1) L. Birnoschi and N. F. Chilton, “Hyperion: A new computational tool for relativistic ab initio hyperfine coupling”, *Journal of Chemical Theory and Computation*, 2022, **18**, 4719–4732.

(2) K. G. Dyall, “Interfacing relativistic and nonrelativistic methods. I. Normalized elimination of the small component in the modified Dirac equation”, *The Journal of chemical physics*, 1997, **106**, 9618–9626.

(3) L. Wenjian and P. Daoling, “Exact two-component Hamiltonians revisited”, *Journal of Chemical Physics*, 2009, **131**, 031104.

(4) W. Liu, “Ideas of relativistic quantum chemistry”, *Molecular Physics*, 2010, **108**, 1679–1706.

(5) T. Saue, “Relativistic Hamiltonians for chemistry: A primer”, *ChemPhysChem*, 2011, **12**, 3077–3094.

(6) D. Peng and M. Reiher, “Exact decoupling of the relativistic Fock operator”, *Theoretical Chemistry Accounts*, 2012, **131**, 1–20.

(7) M. Reiher and A. Wolf, *Relativistic quantum chemistry: the fundamental theory of molecular science*, John Wiley & Sons, 2014.

(8) W. Kutzelnigg and W. Liu, “Quasirelativistic theory equivalent to fully relativistic theory”, *The Journal of chemical physics*, 2005, **123**, 241102.

(9) W. Kutzelnigg and W. Liu*, “Quasirelativistic theory I. Theory in terms of a quasi-relativistic operator”, *Molecular Physics*, 2006, **104**, 2225–2240.

(10) W. Liu and D. Peng, “Infinite-order quasirelativistic density functional method based on the exact matrix quasirelativistic theory”, *The Journal of chemical physics*, 2006, **125**, 044102.

(11) M. Iliaš and T. Saue, “An infinite-order two-component relativistic Hamiltonian by a simple one-step transformation”, *The Journal of chemical physics*, 2007, **126**, 064102.

(12) M. Filatov and K. G. Dyall, “On convergence of the normalized elimination of the small component (NESC) method”, *Theoretical Chemistry Accounts*, 2007, **117**, 333–338.

(13) D. Peng, W. Liu, Y. Xiao and L. Cheng, “Making four-and two-component relativistic density functional methods fully equivalent based on the idea of “from atoms to molecule””, *The Journal of chemical physics*, 2007, **127**, 104106.

(14) W. Liu and W. Kutzelnigg, “Quasirelativistic theory. II. Theory at matrix level”, *The Journal of chemical physics*, 2007, **126**, 114107.

(15) J. Sikkema, L. Visscher, T. Saue and M. Iliaš, “The molecular mean-field approach for correlated relativistic calculations”, *The Journal of chemical physics*, 2009, **131**, 124116.

(16) L. F. Chibotaru, in *Advances in Chemical Physics*, John Wiley & Sons, Ltd, 2013, pp. 397–519.

(17) A. Formanuik, A.-M. M. Ariciu, F. Ortu, R. Beekmeyer, A. Kerridge, F. Tuna, E. J. L. McInnes and D. P. Mills, “Actinide covalency measured by pulsed electron paramagnetic resonance spectroscopy”, *Nature Chemistry*, 2017, **9**, 578–583.
